# Visible-light-induced nickel-catalyzed α-hydroxytrifluoroethylation of alkyl carboxylic acids: Access to trifluoromethyl alkyl acyloins

**DOI:** 10.3762/bjoc.19.98

**Published:** 2023-09-11

**Authors:** Feng Chen, Xiu-Hua Xu, Zeng-Hao Chen, Yue Chen, Feng-Ling Qing

**Affiliations:** 1 Key Laboratory of Organofluorine Chemistry, Shanghai Institute of Organic Chemistry, University of Chinese Academy of Sciences, Chinese Academy of Sciences, 345 Lingling Lu, Shanghai 200032, China; 2 Shandong Dongyue Polymer Material Co., Ltd., Zibo 256401, China

**Keywords:** alkyl carboxylic acids, cross coupling, EDA complex, nickel catalysis, trifluoromethyl acyloins

## Abstract

A visible-light-induced nickel-catalyzed cross coupling of alkyl carboxylic acids with *N*-trifluoroethoxyphthalimide is described. Under purple light irradiation, an α-hydroxytrifluoroethyl radical generated from a photoactive electron donor–acceptor complex between Hantzsch ester and *N*-trifluoroethoxyphthalimide was subsequently engaged in a nickel-catalyzed coupling reaction with in situ-activated alkyl carboxylic acids. This convenient protocol does not require photocatalysts and metal reductants, providing a straightforward and efficient access to trifluoromethyl alkyl acyloins in good yields with broad substrate compatibility. The complex bioactive molecules were also compatible with this catalytic system to afford the corresponding products.

## Introduction

**Figure 1:**
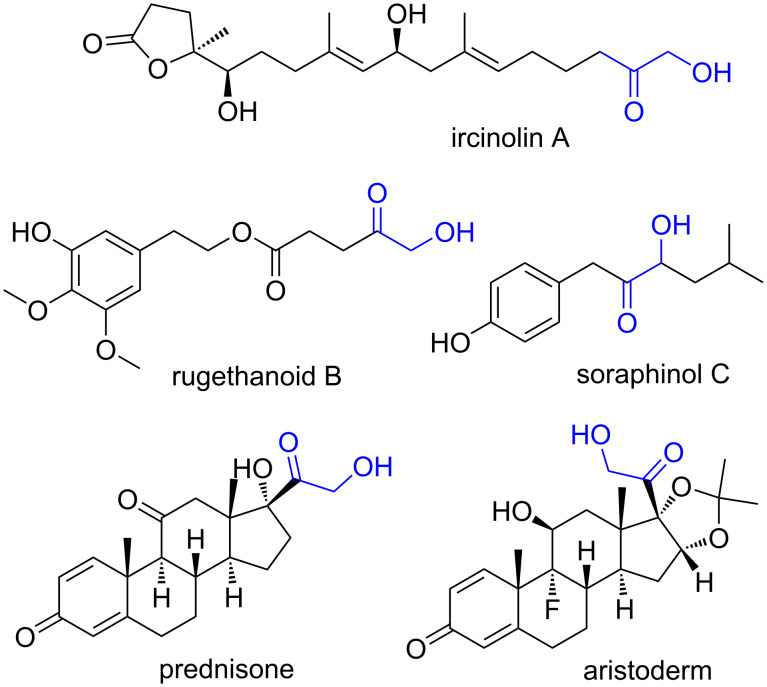
Selected natural products and pharmaceuticals bearing acyloins.

Acyloins (also known as α-hydroxy ketones) are widely found as structural motif in natural products [1–7] and bioactive molecules [8–11] (Figure 1). They can also be used as building blocks in organic synthesis [12–14], involved in numerous transformations to other important functional groups such as dicarbonyls [15], diols [16], amino ketones [17] and so on. Recently, the introduction of a trifluoromethyl group into organic molecules has received great attention due to their wide applicability in medicinal [18–19] and materials [20–21] chemistry. The toxicological experiments showed that trifluoromethyl acyloins can selectively induce apoptosis in human oral cancer cells [22–23] and have therefore attracted much more attention. However, trifluoromethyl acyloins were not widely used due to the challenge associated with their synthesis.

**Scheme 1:**
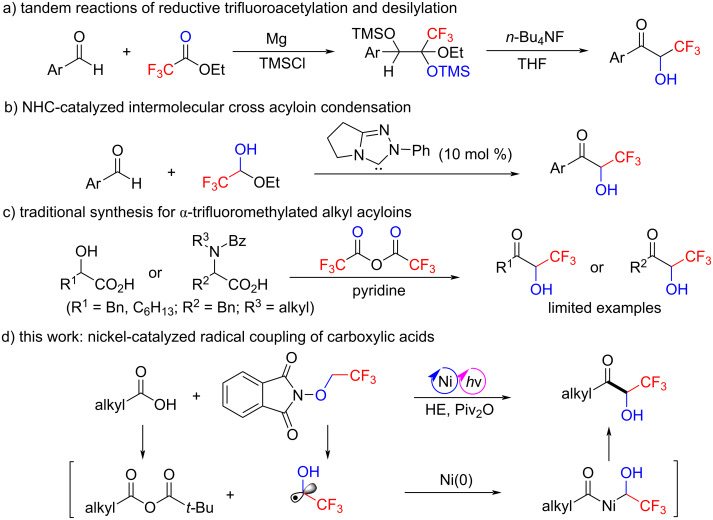
Strategies for the synthesis of α-trifluoromethyl acyloins.

Certain progress has been made in the synthesis of trifluoromethyl aromatic acyloins. Maekawa’s group [24] developed a tandem reaction of the reductive coupling between arylaldehydes and ethyl trifluoroacetate in the presence of magnesium and chlorotrimethylsilane, followed by desilylation to produce the trifluoromethyl aromatic acyloins (Scheme 1a). Anand’s group [25] demonstrated that a NHC-catalyzed selective acyloin condensation between aromatic aldehydes and trifluoroacetaldehyde ethyl hemiacetal afforded the analogous products (Scheme 1b). In comparison, the synthesis of trifluoromethyl aliphatic acyloins is still challenging, and to the best of our knowledge, only Kawase's group [26] reported the preparation of such compounds starting from α-hydroxy acids or α-amino acids in the presence of trifluoroacetic anhydride and pyridine with very limited substrate scope (Scheme 1c). Therefore, the development of a more general and practical method for the synthesis of trifluoromethyl aliphatic acyloins is highly desirable.

Carboxylic acids are widely used in organic reactions because they are chemically stable, less toxic, and commercially available in a large structural variety [27–30]. The nickel-catalyzed direct conversion of carboxylic acids to ketones is an important chemical transformation [31–38]. However, to the best of our knowledge, this protocol has not been used for the synthesis of fluoroalkylated ketones so far. Very recently, we have developed a visible-light-induced nickel-catalyzed coupling of aryl bromides with an α-hydroxytrifluoroethyl radical for the synthesis of trifluoromethyl aryl alcohols [39]. Encouraged by this work, we envisioned that the nickel-catalyzed coupling of carboxylic acids-derived acyl electrophiles with an α-hydroxytrifluoroethyl radical might be feasible. Herein, we disclose a visible-light-induced nickel-catalyzed cross-coupling of alkyl carboxylic acids with *N*-trifluoroethoxyphthalimide to deliver trifluoromethyl aliphatic acyloins under mild conditions (Scheme 1d). Furthermore, this platform bypasses the need for exogenous photocatalysts, providing a direct and robust access to trifluoromethyl aliphatic acyloins.

## Results and Discussion

Initially, we commenced our exploration by choosing 4-phenylbutyric acid (**1a**) as the model substrate to react with *N*-trifluoroethoxyphthalimide (**2**, Table 1). On basis of the previously reported elegant strategies on direct conversion of in situ-activated carboxylic acids for ketone synthesis [27,35,38], we chose dimethyl dicarbonate (DMDC, **A1**) as the activating reagent. To our delight, the reaction of **1a** and **2** in the presence of NiBr_2_(dtbbpy) (10 mol %), Hantzsch ester (HE) and **A1** in DMAc under the irradiation of purple LEDs afforded the desired coupling product **3a** in 50% yield (Table 1, entry 1). Further screening of other activators (Table 1, entries 2–5) indicated that pivalic anhydride (**A3**) was optimal, delivering **3a** in 56% yield. The yield of **3a** was increased to 74% when 3.0 equiv of H_2_O were added to the reaction mixture [40] (Table 1, entry 6), but the addition of more water did not improve the reaction efficiency further (Table 1, entry 7). The structure of nickel catalysts played a significant role in the reaction efficiency. Switching the Ni catalyst to NiCl_2_(dtbbpy), NiBr_2_(bpy), NiBr_2_(phen) or other nickel salts led to comparable or dramatically decreased yields (Table 1, entries 8–12). Other polar non-protonic solvents such as NMP and DMF led to diminished yields (Table 1, entries 13 and 14).

**Table 1:** Optimization of the reaction conditions.^a^

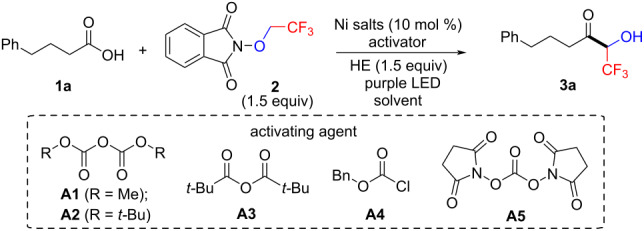				

entry	Ni salt (ligand)	activator	solvent	yield (%)^b^

1	NiBr_2_(dtbbpy)	**A1**	DMAc	50
2	NiBr_2_(dtbbpy)	**A2**	DMAc	0
3	NiBr_2_(dtbbpy)	**A3**	DMAc	56
4	NiBr_2_(dtbbpy)	**A4**	DMAc	0
5	NiBr_2_(dtbbpy)	**A5**	DMAc	0
6^c^	NiBr_2_(dtbbpy)	**A3**	DMAc	74
7^d^	NiBr_2_(dtbbpy)	**A3**	DMAc	68
8^c^	NiCl_2_(dtbbpy)	**A3**	DMAc	37
9^c^	NiBr_2_(bpy)	**A3**	DMAc	67
10^c^	NiBr_2_(phen)	**A3**	DMAc	8
11^c^	NiBr_2_diglyme/4,4-dCO_2_Me-bpy	**A3**	DMAc	61
12^c^	NiBr_2_diglyme/4,4-dMeO-bpy	**A3**	DMAc	65
13^c^	NiBr_2_(dtbbpy)	**A3**	NMP	53
14^c^	NiBr_2_(dtbbpy)	**A3**	DMF	trace

^a^Reaction conditions: **1a** (0.1 mmol), **2** (0.15 mmol), Ni catalyst (0.01 mmol), Hantzsch ester (0.15 mmol), solvent (1.0 mL), activator (0.15 mmol), purple LEDs, 7 h. ^b^Yields determined by ^19^F NMR spectroscopy using trifluoromethoxybenzene as an internal standard. ^c^Adding 3.0 equiv H_2_O. ^d^Adding 10.0 equiv H_2_O.

**Scheme 2:**
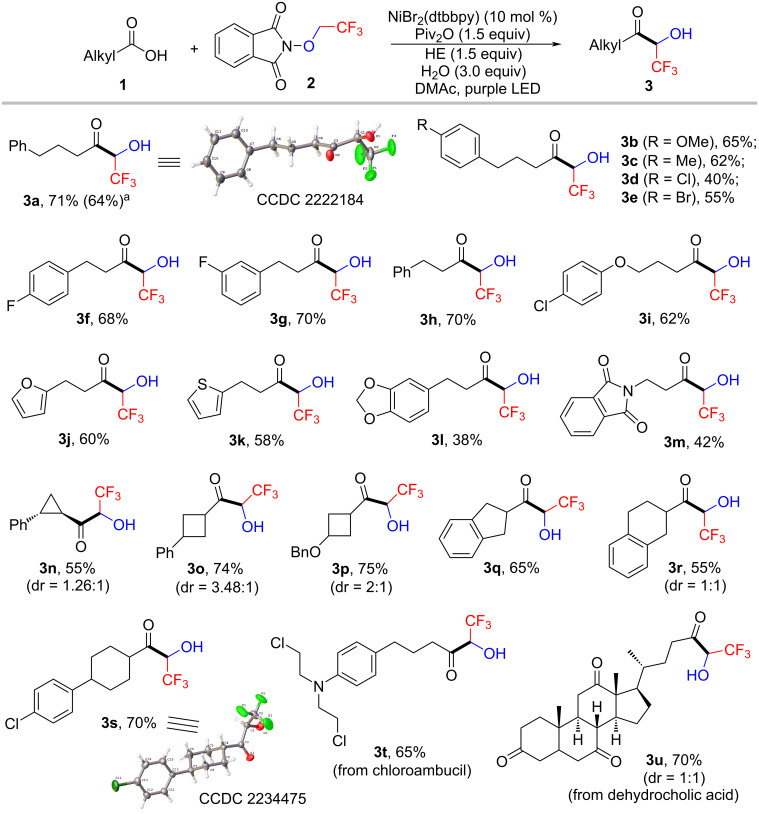
Substrate scope. Standard conditions: a solution of alkyl carboxylic acid **1** (0.4 mmol), **2** (0.6 mmol), NiBr_2_(dtbbpy) (0.04 mmol), Hantzsch ester (0.6 mmol), Piv_2_O (0.6 mmol) and H_2_O (1.2 mmol) in DMAc (4.0 mL) was irradiated by purple LEDs for 7 h. Isolated yields are presented. ^a^The reaction was performed in a 1.0 mmol scale.

Having identified the optimal reaction conditions, we sought to evaluate the substrate scope of this photoinduced nickel-catalyzed cross coupling reaction. As illustrated in Scheme 2, a broad array of aliphatic carboxylic acids reacted smoothly in this protocol, providing the corresponding trifluoromethyl aliphatic acyloins in moderate to excellent yields. This mild reaction showed a good tolerance of a diverse range of functional groups, including methoxy (**3b**), methyl (**3c**), chloro (**3d**,**i**), fluoro (**3f,g**), and ethers (**3i**,**l**,**p**). Notably, aryl bromide (**3e**) was also tolerated in this protocol, probably due to the higher reactivity of the mixed anhydride formed between carboxylic acid and pivalic anhydride than aryl bromide. The halides provided versatile synthetic handles for further transformations. Substrates bearing thiophene (**3k**) furan (**3j**) and other heterocycle (**3l**,**m**) moieties were also applicable to this reaction. This protocol allowed for the coupling of not only primary carboxylic acids but also secondary carboxylic acids (**3n**−**s**). We were pleased to find that cyclic carboxylic acids, including strained 3- and 4-membered rings, participated in this transformation and delivered the corresponding products in good yields. It should be mentioned that aromatic and more sterically hindered tertiary carboxylic acids were unfortunately not compatible with the reaction conditions. The structures of products **3a** and **3s** were unambiguously confirmed by single-crystal X-ray diffraction. Notably, the reaction of **1a** could be easily scaled up to 1.0 mmol scale, affording **3a** in a slightly lower yield. To further demonstrate the amenability toward pharmaceutically active molecules, chloroambucil (**1t**) and dehydrocholic acid (**1u**) were successfully subjected to the reaction conditions, delivering the desired products in moderate yields. However, the analogous reaction with *N*-difluoroethoxyphthalimide as the fluoroalkylating reagent failed to afford the desired product.

**Figure 2:**
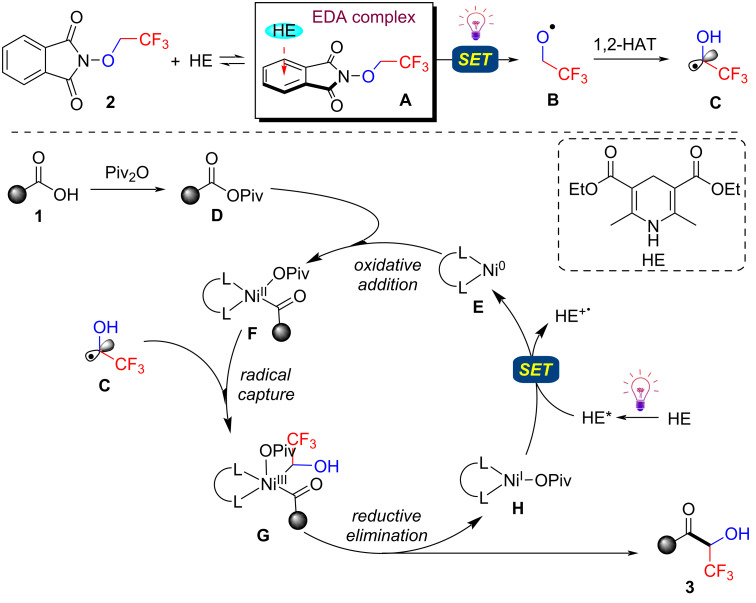
Proposed reaction mechanism.

According to our previous work [39] and literature precedent [27,35,38], a possible mechanism is proposed in Figure 2. The interaction between **2** and HE generates an electron donor–acceptor (EDA) complex **A**, which undergoes a light-induced charge transfer event to give trifluoroethoxyl radical **B**, followed by a 1,2-hydrogen atom transfer (HAT), producing the stable radical **C**. For the nickel cycle, it is initiated by oxidative addition of Ni(0) catalyst **E** to acyl electrophile **D** formed in situ from carboxylic acid **1** with pivalic anhydride as activator to afford Ni(II) intermediate **F**. Subsequently, trapping of the alkyl radical **C** generates high-valent Ni(III) intermediate **G**, which undergoes facile reductive elimination to furnish the final coupling product **3** and Ni(I) intermediate **H**. The single-electron transfer (SET) reduction of intermediate **H** (*E*_red_(Ni^I^/Ni^0^) = −1.17 V vs SCE [41]) by photoexcited HE* (*E*_red_(HE^*^/HE**^·^**^+^) = −2.28 V vs SCE [42]) regenerates the active Ni(0) species **E** and closes the catalytic cycle.

## Conclusion

In conclusion, we have demonstrated a visible-light-induced nickel-catalyzed radical cross coupling of readily available alkyl carboxylic acids with *N*-trifluoroethoxyphthalimide. The present study provides a mild and efficient method for the preparation of trifluoromethyl alkyl acyloins in moderate to high yields and with good functional group compatibility. Further studies on expansion of the reaction scope and development of related enantioselective photoinduced nickel-catalyzed radical cross-coupling reactions are currently underway in our laboratory.

## Experimental

**General procedure for the visible-light-induced nickel-catalyzed cross coupling of alkyl carboxylic acids with *N*-trifluoroethoxyphthalimide:** In the glove box with nitrogen atmosphere, to an 8 mL vial equipped with a magnetic stir bar, NiBr_2_(dtbbpy) (19.6 mg, 0.04 mmol, 10 mol %), alkyl carboxylic acid **1** (0.4 mmol, 1.0 equiv), *N*-trifluoroethoxyphthalimide (**2**, 147.1 mg, 0.6 mmol, 1.5 equiv), Hantzsch ester (152.0 mg, 0.6 mmol, 1.5 equiv), and anhydrous *N*,*N*-dimethylacetamide (4.0 mL) were added. The vial was then re-capped and taken out of the glove box, and Piv_2_O (111.8 mg, 0.6 mmol, 1.5 equiv) and H_2_O (21.6 mg, 1.2 mmol, 3.0 equiv) were added. The vial was sealed and the reaction mixture was then stirred under irradiation by purple LEDs (λ_max_ = 399 nm) for 7 h. After the reaction was complete, the reaction mixture was poured into water and extracted with EtOAc. The combined organic phase was separated and washed with brine, dried over Na_2_SO_4_, and concentrated under vacuum. The resulting residue was purified by silica gel flash column chromatography to give the coupling product **3**.

## Supporting Information

File 1 Experimental procedures, product characterization, and copies of NMR spectra.
