# Supplementary material for: Visible-light-induced nickel-catalyzed α-hydroxytrifluoroethylation of alkyl carboxylic acids: Access to trifluoromethyl alkyl acyloins
Source: Beilstein J Org Chem. 2023 Sep 11;19:1372–8. doi: 10.3762/bjoc.19.98 (PMC10509543; doi:10.3762/bjoc.19.98)

BEILSTEIN JOURNAL OF ORGANIC CHEMISTRY

Supporting Information

for

Visible-light-induced nickel-catalyzed α-hydroxytrifluoroethylation of alkyl carboxylic acids: Access to trifluoromethyl alkyl acyloins

Feng Chen, Xiu-Hua Xu, Zeng-Hao Chen, Yue Chen and Feng-Ling Qing

Beilstein J. Org. Chem. **2023,** *19,* 1372–1378. doi:10.3762/bjoc.19.98

Experimental procedures, product characterization, and copies of NMR spectra

License and Terms: This is a supporting information file under the terms of the Creative Commons Attribution License (https://creativecommons.org/licenses/by/4.0). Please note that the reuse, redistribution and reproduction in particular requires that the author(s) and source are credited and that individual graphics may be subject to special legal provisions.
The license is subject to the *Beilstein Journal of Organic Chemistry* terms and conditions: (https://www.beilstein-journals.org/bjoc/terms)

Table of Contents

1. General information S2

2. Preparation of substrates S3

3. General procedures and substrate scope S3

4. Scale-up reaction S12

5. ORTEP drawing of the X-ray crystallographic structure................................S13

6. References S17

7. Copies of ^{1}H NMR, ^{19}F NMR and ^{13}C NMR spectra of products....................S18

1. General information

^{1}H, ^{19}F NMR spectra and ^{13}C NMR spectra were recorded on an Agilent AM400 spectrometer and Bruker AM400 spectrometer. ^{1}H NMR and ^{13}C NMR chemical shifts were reported in ppm relative to chloroform (^{1}H, δ 7.26; ^{13}C, δ 77.0) or acetone ((^{1}H, δ 2.05; ^{13}C, δ 29.8) and ^{19}F NMR chemical shifts were determined relative to $CFCl_3$ as internal standard. Chemical shifts (δ) were reported in ppm and coupling constants (*J*) are in Hertz (Hz). The following abbreviations were used to explain the multiplicities: s = singlet, d = doublet, t = triplet, q = quartet, m = multiplet. IR spectra were recorded on a Thermo Scientific Nicolet iS5 Fourier transform infrared (FT-IR) spectrometer and were reported in terms of wavenumber of absorption (cm^{-1}). Detection of melting point was conducted on the SGW X-4A microscopic melting point meter. High-resolution mass spectrometry (HRMS) was performed on a Waters Premier GC-TOF MS instrument with electron impact (EI) mode, on a JEOL-AccuTOF-GCv4G-GCT MS instrument with field ionization (FI) mode or on a Thermo Scientific Q Exactive HF Orbitrap-FTMS instrument with electrospray ionization (ESI) mode.

All photochemical reactions were performed in 4 mL screw cap scintillation vials (unless noted otherwise) under an inert atmosphere at room temperature and irradiated with two 20 W purple LEDs (maximum emission wavelength = 399 nm).

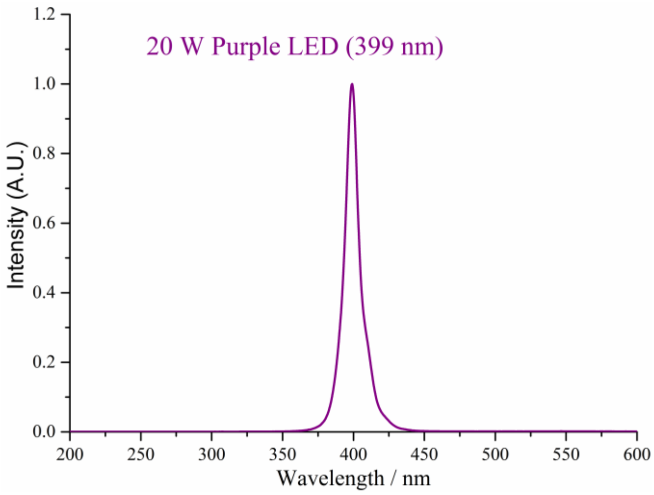

Figure S1: The emission spectra of the purple LEDs.

2. Preparation of substrates

Carboxylic acid substrates and the nickel catalysts ($NiBr_2$(dtbbpy), *CAS:* 1894189-67-3; $NiBr_2$(bpy), *CAS:* 46389-47-3; $NiBr_2$(Phen), *CAS:* 48165-50-0; $NiCl_2$(dtbbpy), *CAS:* 1034901-50-2) were obtained commercially and used as received.

And the *N*-alkoxyphthalimide reagent **2** (2-(2,2,2-trifluoroethoxy)isoindoline-1,3-dione)[1] were synthesized according to the literature procedures.

3. General procedures and substrate scope

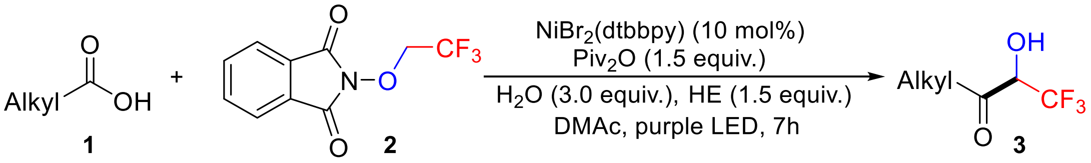

In the glove box with nitrogen atmosphere, to a 8 mL vial equipped with a magnetic stir bar, $NiBr_2$(dtbbpy) (19.6 mg, 0.04 mmol, 10 mol %), alkyl carboxylic acid 1(0.4 mmol, 1.0 equiv), **2** (147.1 mg, 0.6 mmol, 1.5 equiv), Hantzsch ester (152.0 mg, 0.6 mmol, 1.5 equiv), and 4.0 mL of anhydrous *N,N*-dimethylacetamide were added. The vial was then re-capped and taken out of the glove box, and Piv_2O (111.8 mg, 0.6 mmol, 1.5 equiv), H_2O (21.6 mg, 1.2 mmol, 3.0 equiv) were added. The vial was sealed with parafilm and the reaction mixture was then stirred under irradiation by purple LEDs (λ_{max} = 399 nm) for 7 h. After the reaction was complete, the reaction mixture was poured into water and extracted with EtOAc. The combined organic phase was separated and washed with brine, dried over Na_2SO_4, and concentrated under vacuum. The resulting residue was purified by silica gel flash column chromatography to give the product.

1,1,1-Trifluoro-2-hydroxy-6-phenylhexan-3-one (3a)

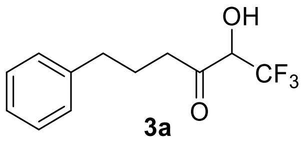

3a

The residue was purified by silica gel column chromatography (hexane/acetone = 15:1) to afford **3a** (69.9 mg, 71%) as a white solid. **M.P.** 90.7-91.3 °C. **^{1}H NMR** (400 MHz, $CDCl_3$) δ 7.33-7.28 (m, 2H), 7.24-7.20 (m, 1H), 7.19-7.15 (m, 2H), 4.49-4.41 (m, 1H), 4.02 (s, 1H), 2.79-2.56 (m, 4H), 2.09-1.99 (m, 2H). **^{19}F NMR** (376 MHz, $CDCl_3$) δ -74.14 (d, *J* = 7.6 Hz, 3F). **^{13}C NMR** (101 MHz, $CDCl_3$) δ 202.7, 140.7, 128.6, 128.4, 126.3, 122.4 (q, *J* = 283.8 Hz), 74.8 (q, *J* = 31.7 Hz), 38.8 (q, *J* = 2.2 Hz), 34.7, 24.7. **IR (thin film)**: *v* 3405, 2955, 1725, 1467, 1270, 1142, 1118, 764, 705 cm^{-1}. **MS** (EI): *m/z* 246.1 [M^+]; **HRMS** (EI-TOF) *m/z*: [M^+] Calcd. for $C_{12}H_{13}F_3O_2$ 246.0862; Found: 246.0866.

1,1,1-Trifluoro-2-hydroxy-6-(4-methoxyphenyl)hexan-3-one (3b)

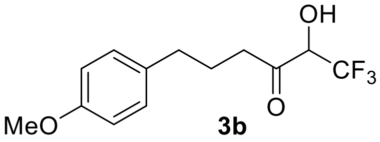

3b

The residue was purified by silica gel column chromatography (hexane/acetone = 15:1) to afford **3b** (71.8 mg, 65%) as a white solid. **M.P.** 65.5-66.7 °C. **^{1}H NMR** (400 MHz, $CDCl_3$) δ 7.07 (d, *J* = 8.5 Hz, 2H), 6.84 (d, *J* = 8.6 Hz, 2H), 4.44 (q, *J* = 7.6 Hz, 1H), 4.01 (s, 1H), 3.79 (s, 3H), 2.75-2.54 (m, 4H), 2.06-1.94 (m, 2H). **^{19}F NMR** (376 MHz, $CDCl_3$) δ -74.14 (d, *J* = 7.5 Hz, 3F). **^{13}C NMR** (101 MHz, $CDCl_3$) δ 202.7, 158.0, 132.7, 129.3, 122.4 (q, *J* = 283.1 Hz), 113.9, 74.8 (q, *J* = 31.7 Hz), 55.2, 38.7 (q, *J* = 2.4 Hz), 33.8, 24.9. **IR (thin film)**: *v* 3438, 2941, 1725, 1614, 1515, 1342, 1249, 1146, 1006, 676, 557 cm^{-1}. **MS** (EI): *m/z* 276.1 [M^+]; **HRMS** (EI-TOF) *m/z*: [M^+] Calcd. for $C_{13}H_{15}F_3O_3$ 276.0968; Found: 276.0967.

1,1,1-Trifluoro-2-hydroxy-6-(*p*-tolyl)hexan-3-one (3c)

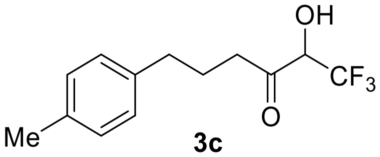

3c

The residue was purified by silica gel column chromatography (hexane/acetone = 15:1) to afford **3c** (64.5 mg, 62%) as a white solid. **M.P.** 60.7-61.5 °C. **^{1}H NMR** (400 MHz, $CDCl_3$) δ 7.14-7.04 (m, 4H), 4.45 (q, *J* = 7.6 Hz, 1H), 3.86 (s, 1H), 2.78-2.56 (m, 4H), 2.33 (s, 3H), 2.06-1.96 (m, 2H). **^{19}F NMR** (376 MHz, $CDCl_3$) δ -74.14 (d, *J* = 7.5 Hz, 3F). **^{13}C NMR** (101 MHz, $CDCl_3$) δ 202.7, 137.6, 135.7, 129.2, 128.2, 122.4 (q, *J* = 283.1 Hz), 74.8 (q, *J* = 31.7 Hz), 38.8 (q, *J* = 2.4 Hz), 34.2, 24.8, 21.0. **IR (thin film)**: *v* 3416, 2925, 1725, 1516, 1458, 1346, 1249, 1080, 805, 676 cm^{-1}. **MS** (EI): *m/z* 260.1 [M^+]; **HRMS** (EI-TOF) *m/z*: [M^+] Calcd. for $C_{13}H_{15}F_3O_2$ 260.1019; Found: 260.1023.

6-(4-Chlorophenyl)-1,1,1-trifluoro-2-hydroxyhexan-3-one (3d)

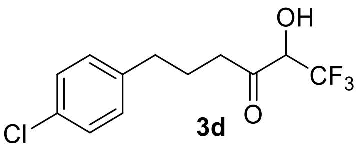

3d

The residue was purified by silica gel column chromatography (hexane/acetone = 15:1) to afford **3d** (44.9 mg, 40%) as a white solid. **M.P.** 75.4-77.0 °C. **^{1}H NMR** (400 MHz, $CDCl_3$) δ 7.25 (d, *J* = 8.3 Hz, 2H), 7.08 (d, *J* = 8.3 Hz, 2H), 4.45 (q, *J* = 7.5 Hz, 1H), 3.84 (s, 1H), 2.75-2.53 (m, 4H), 2.06-1.91 (m, 2H). **^{19}F NMR** (376 MHz, $CDCl_3$) δ -74.14 (d, *J* = 7.5 Hz, 3F). **^{13}C NMR** (101 MHz, $CDCl_3$) δ 202.5, 139.2, 132.0, 129.7, 128.6, 122.4 (q, *J* = 283.3 Hz), 74.8 (q, *J* = 31.5 Hz), 38.7 (q, *J* = 2.3 Hz), 34.0, 24.5. **IR (thin film)**: *v* 3416, 2951, 1727, 1493, 1460, 1407, 1269, 1142, 1004, 806, 527 cm^{-}

[1]. **MS** (EI): *m/z* 280.0 [M^+]; **HRMS** (EI-TOF) *m/z*: [M^+] Calcd. for $C_{12}H_{12}ClF_3O_2$ 280.0472; Found: 280.0476.

6-(4-Bromophenyl)-1,1,1-trifluoro-2-hydroxyhexan-3-one (3e)

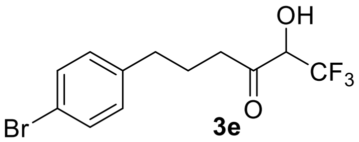

3e

The residue was purified by silica gel column chromatography (hexane/acetone = 15:1) to afford **3e** (71.5 mg, 55%) as a white solid. **M.P.** 91.2-92.7 °C. **^{1}H NMR** (400 MHz, $CDCl_3$) δ 7.41 (d, *J* = 8.3 Hz, 2H), 7.04 (d, *J* = 8.3 Hz, 2H), 4.46 (q, *J* = 7.5 Hz, 1H), 4.00 (s, 1H), 2.78-2.55 (m, 4H), 2.05-1.94 (m, 2H). **^{19}F NMR** (376 MHz, $CDCl_3$) δ -74.12 (d, *J* = 7.5 Hz, 3F). **^{13}C NMR** (101 MHz, $CDCl_3$) δ 202.5, 139.7, 131.6, 130.1, 122.4 (q, *J* = 283.2 Hz), 120.0, 74.8 (q, *J* = 31.7 Hz), 38.7 (q, *J* = 2.4 Hz), 34.1, 24.4. **IR (thin film)**: *v* 3422, 2950, 1724, 1489, 1341, 1259, 1144, 1010, 836, 677 cm^{-1}. **MS** (EI): *m/z* 324.0 [M^+]; **HRMS** (EI-TOF) *m/z*: [M^+] Calcd. for $C_{12}H_{12}BrF_3O_2$ 323.9967; Found: 323.9963.

1,1,1-Trifluoro-5-(4-fluorophenyl)-2-hydroxypentan-3-one (3f)

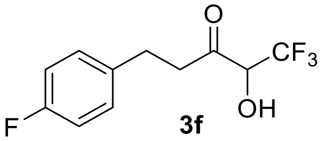

3f

The residue was purified by silica gel column chromatography (hexane/acetone = 15:1) to afford **3f** (68.1 mg, 68%) as a sticky yellowish oil. **^{1}H NMR** (400 MHz, $CDCl_3$) δ 7.16-7.12 (m, 2H), 7.02-6.95 (m, 2H), 4.45 (q, *J* = 7.5 Hz, 1H), 4.02 (s, 1H), 3.08-2.87 (m, 4H). **^{19}F NMR** (376 MHz, $CDCl_3$) δ -74.09 (d, *J* = 7.6 Hz, 3F), -116.41 – -116.51 (m, 1F). **^{13}C NMR** (101 MHz, $CDCl_3$) δ 201.9, 161.6 (d, *J* = 244.8 Hz), 135.2, 129.7 (d, *J* = 8.0 Hz), 122.4 (q, *J* = 283.3 Hz), 115.5 (d, *J* = 21.3 Hz), 75.0 (q, *J* = 31.6 Hz), 41.4, 28.4. **IR (thin film)**: *v* 3454, 2929, 1731, 1603, 1512, 1224, 1119, 825, 538 cm^{-1}. **MS** (EI): *m/z* 250.1 [M^+]; **HRMS** (EI-TOF) *m/z*: [M^+] Calcd. for $C_{11}H_{10}F_4O_2$ 250.0611; Found: 250.0609.

1,1,1-Trifluoro-5-(3-fluorophenyl)-2-hydroxypentan-3-one (3g)

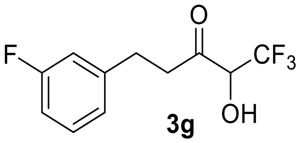

3g

The residue was purified by silica gel column chromatography (hexane/acetone = 15:1) to afford **3g** (70.1 mg, 70%) as a sticky yellowish oil. **^{1}H NMR** (400 MHz, $CDCl_3$) δ 7.29-7.23 (m, 1H), 6.98-6.87 (m, 3H), 4.53-4.41 (m, 1H), 3.99 (d, *J* = 6.3 Hz, 1H), 3.09-2.91 (m, 4H). **^{19}F NMR** (376 MHz, $CDCl_3$) δ -74.08 (d, *J* = 7.5 Hz, 3F), -113.00

– -113.08 (m, 1F). **^{13}C NMR** (101 MHz, $CDCl_3$) δ 201.7, 162.9 (d, *J* = 246.1 Hz), 142.0 (d, *J* = 7.3 Hz), 130.2 (d, *J* = 8.3 Hz), 123.9 (d, *J* = 3.0 Hz), 122.3 (q, *J* = 283.2 Hz), 115.2 (d, *J* = 21.1 Hz), 113.5 (d, *J* = 20.9 Hz), 75.0 (q, *J* = 31.7 Hz), 40.9 (q, *J* = 2.3 Hz), 28.8. **IR (thin film)**: *v* 3448, 2926, 2855, 1730, 1618, 1491, 1190, 1119, 782, 520 cm^{-1}. **MS** (EI): *m/z* 250.1 [M^+]; **HRMS** (EI-TOF) *m/z*: [M^+] Calcd. for $C_{11}H_{10}F_4O_2$ 250.0611; Found: 250.0614.

1,1,1-Trifluoro-2-hydroxy-5-phenylpentan-3-one (3h)

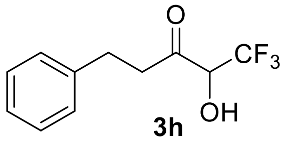

3h

The residue was purified by silica gel column chromatography (hexane/acetone = 15:1) to afford **3h** (65.0 mg, 70%) as a sticky yellowish oil. **^{1}H NMR** (400 MHz, $CDCl_3$) δ 7.34-7.29 (m, 2H), 7.26-7.16 (m, 3H), 4.51-4.39 (m, 1H), 4.02 (d, *J* = 6.2 Hz, 1H), 3.09-2.92 (m, 4H). **^{19}F NMR** (376 MHz, $CDCl_3$) δ -74.04 (d, *J* = 7.5 Hz, 3F). **^{13}C NMR** (101 MHz, $CDCl_3$) δ 202.1, 139.5, 128.7, 128.2, 126.6, 122.4 (q, *J* = 283.2 Hz), 75.0 (q, *J* = 31.7 Hz), 41.3 (q, *J* = 2.4 Hz), 29.3. **IR (thin film)**: *v* 3448, 3030, 2928, 1729, 1497, 1250, 1189, 1119, 751, 700 cm^{-1}. **MS** (EI): *m/z* 232.1 [M^+]; **HRMS** (EI-TOF) *m/z*: [M^+] Calcd. for $C_{11}H_{11}F_3O_2$ 232.0706; Found: 232.0709.

6-(4-Chlorophenoxy)-1,1,1-trifluoro-2-hydroxyhexan-3-one (3i)

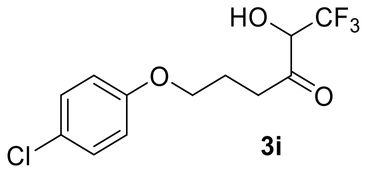

3i

The residue was purified by silica gel column chromatography (hexane/acetone = 15:1–10:1) to afford **3i** (73.6 mg, 62%) as a sticky yellowish oil. **^{1}H NMR** (400 MHz, $CDCl_3$) δ 7.25-7.20 (m, 2H), 6.82-6.76 (m, 2H), 4.62-4.54 (m, 1H), 4.04 (d, *J* = 6.3 Hz, 1H), 4.00-3.93 (m, 2H), 2.96-2.81 (m, 2H), 2.24-2.16 (m, 2H). **^{19}F NMR** (376 MHz, $CDCl_3$) δ -74.16 (d, *J* = 7.8 Hz, 3F). **^{13}C NMR** (101 MHz, $CDCl_3$) δ 202.4, 157.0, 129.4, 125.9, 122.4 (q, *J* = 283.3 Hz), 115.6, 74.8 (q, *J* = 31.6 Hz), 66.5, 36.4 (q, *J* = 2.4 Hz), 23.3. **IR (thin film)**: *v* 3454, 2919, 1729, 1597, 1494, 1244, 1093, 826, 663 cm^{-1}. **MS** (EI): *m/z* 296.0 [M^+]; **HRMS** (EI-TOF) *m/z*: [M^+] Calcd. for $C_{12}H_{12}ClF_3O_3$ 296.0422; Found: 296.0421.

1,1,1-Trifluoro-5-(furan-2-yl)-2-hydroxypentan-3-one (3j)

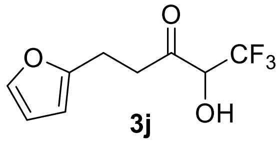

3j

The residue was purified by silica gel column chromatography (hexane/acetone = 15:1) to afford **3j** (53.3 mg, 60%) as a sticky yellowish oil. **^{1}H NMR** (400 MHz, $CDCl_3$) δ 7.33-7.27 (m, 1H), 6.29-6.27 (m, 1H), 6.05-6.02 (m, 1H), 4.57-4.45 (m, 1H), 4.02 (d, *J* = 6.2 Hz, 1H), 3.09-2.97 (m, 4H). **^{19}F NMR** (376 MHz, $CDCl_3$) δ -74.16 (d, *J* = 7.5 Hz, 3F). **^{13}C NMR** (101 MHz, $CDCl_3$) δ 201.7, 152.9, 141.5, 122.4 (q, *J* = 283.2 Hz), 110.3, 105.8, 74.9 (q, *J* = 31.7 Hz), 38.1 (q, *J* = 2.4 Hz), 21.8. **IR (thin film)**: *ν* 3406, 2959, 2926, 1731, 1508, 1407, 1266, 1000, 741, 598 cm^{-1}. **MS** (EI): *m/z* 222.1 [M^+]; **HRMS** (EI-TOF) *m/z*: [M^+] Calcd. for $C_9H_9F_3O_3$ 222.0498; Found: 222.0501.

1,1,1-Trifluoro-2-hydroxy-5-(thiophen-2-yl)pentan-3-one (3k)

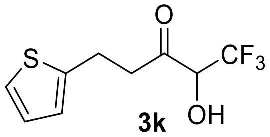

3k

The residue was purified by silica gel column chromatography (hexane/acetone = 15:1) to afford **3k** (55.3 mg, 58%) as a sticky yellowish oil. **^{1}H NMR** (400 MHz, $CDCl_3$) δ 7.17-7.13 (m, 1H), 6.96-6.90 (m, 1H), 6.86-6.78 (m, 1H), 4.54-4.42 (m, 1H), 4.02 (d, *J* = 5.5 Hz, 1H), 3.27-3.21 (m, 2H), 3.15-2.96 (m, 2H). **^{19}F NMR** (376 MHz, $CDCl_3$) δ -74.07 (d, *J* = 7.5 Hz, 3F). **^{13}C NMR** (101 MHz, $CDCl_3$) δ 201.6, 141.9, 127.0, 125.1, 123.8, 122.3 (q, *J* = 283.3 Hz), 75.1 (q, *J* = 31.7 Hz), 41.5 (q, *J* = 2.3 Hz), 23.4. **IR (thin film)**: *ν* 3455, 2926, 2856, 1731, 1441, 1250, 1116, 996, 700 cm^{-1}. **MS** (EI): *m/z* 238.0 [M^+]; **HRMS** (EI-TOF) *m/z*: [M^+] Calcd. for $C_9H_9F_3O_2S$ 238.0270; Found: 238.0271.

5-(Benzo[*d*][1,3]dioxol-5-yl)-1,1,1-trifluoro-2-hydroxypentan-3-one (3l)

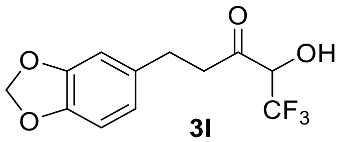

3l

The residue was purified by silica gel column chromatography (hexane/acetone = 15:1) to afford **3l** (42.0 mg, 38%) as a sticky yellowish oil. **^{1}H NMR** (400 MHz, $CDCl_3$) δ 6.75-6.61 (m, 3H), 5.93 (s, 2H), 4.50-4.40 (m, 1H), 4.04 (d, *J* = 6.2 Hz, 1H), 3.03-2.87 (m, 4H). **^{19}F NMR** (376 MHz, $CDCl_3$) δ -74.04 (d, *J* = 7.5 Hz, 3F). **^{13}C NMR** (101 MHz, $CDCl_3$) δ 202.1, 147.8, 146.2, 133.3, 122.4 (q, *J* = 283.3 Hz), 121.1, 108.7, 108.4, 100.9, 75.0 (q, *J* = 31.6 Hz), 41.6 (q, *J* = 2.2 Hz), 29.0. **IR (thin film)**: *ν* 3444, 2905, 1729, 1505, 1493, 1247, 1189, 1117, 1040, 931, 810 cm^{-1}. **MS** (EI): *m/z* 276.1 [M^+]; **HRMS** (EI-TOF) *m/z*: [M^+] Calcd. for $C_{12}H_{11}F_3O_4$ 276.0604; Found: 276.0606.

2-(5,5,5-Trifluoro-4-hydroxy-3-oxopentyl)isoindoline-1,3-dione (3m)

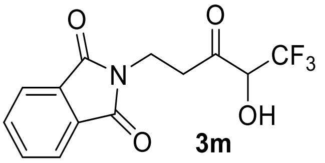

3m

The residue was purified by silica gel column chromatography (hexane/acetone = 15:1) to afford **3m** (51.8 mg, 42%) as a white solid. **M.P.** 148.4-149.0 ºC. **^{1}H NMR** (400 MHz, $(CD_3)_2CO$) δ 7.87-7.82 (m, 4H), 6.19 (d, *J* = 6.7 Hz, 1H), 4.79 (q, *J* = 7.9 Hz, 1H), 3.95 (t, *J* = 7.2 Hz, 2H), 3.29-3.14 (m, 2H). **^{19}F NMR** (376 MHz, $(CD_3)_2CO$) δ -75.44 (d, *J* = 8.0 Hz, 3F). **^{13}C NMR** (101 MHz, $(CD_3)_2CO$) δ 203.3, 168.5, 135.0, 133.1, 124.3 (q, *J* = 282.8 Hz), 123.7, 75.2 (q, *J* = 29.6 Hz), 38.2 (q, *J* = 1.9 Hz), 33.2. **IR (thin film)**: *v* 3434, 2924, 1770, 1709, 1244, 1180, 992, 719, 529 cm^{-1}. **MS** (FI): *m/z* 301.1 [M$^+$]; **HRMS** (FI-TOF) *m/z*: [M$^+$] Calcd. for $C_{13}H_{10}F_3NO_4$ 301.0556; Found: 301.0548.

3,3,3-Trifluoro-2-hydroxy-1-((1*R*,2*R*)-2-phenylcyclopropyl)propan-1-one (3n)

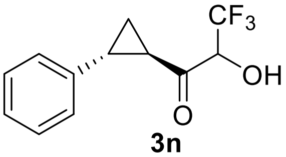

3n

The residue was purified by silica gel column chromatography (hexane/acetone = 15:1) to afford **3n** (53.7 mg, 55%) as a sticky yellowish oil. Product was isolated as a 1:0.8 mixture of two isomers. **^{1}H NMR** (400 MHz, $CDCl_3$) δ 7.36-7.23 (m, 3H), 7.17-7.11 (m, 2H), 4.77-4.67 (m, 1H), 4.18-4.10 (m, 1H), 2.86-2.73 (m, 1H), 2.47-2.39 (m, 1H), 2.00-1.89 (m, 1H), 1.77-1.67 (m, 1H). **^{19}F NMR** (376 MHz, $CDCl_3$) δ -73.83 (d, *J* = 7.5 Hz, 1.67F), -73.98 (d, *J* = 7.4 Hz, 1.33F). **^{13}C NMR** (101 MHz, $CDCl_3$) δ 201.1, 201.0, 138.5, 138.3, 128.7, 128.6, 127.2, 126.6, 126.1, 122.6 (q, *J* = 283.5 Hz), 122.5 (q, *J* = 283.3 Hz),75.2 (q, *J* = 31.5 Hz), 32.8, 32.1, 30.3 (q, *J* = 3.2 Hz), 29.8 (q, *J* = 2.8 Hz), 21.3, 20.0. **IR (thin film)**: *v* 3447, 3032, 2929, 1704, 1458, 1398, 1247, 1127, 866, 727 cm^{-1}. **MS** (EI): *m/z* 244.1 [M$^+$]; **HRMS** (EI-TOF) *m/z*: [M$^+$] Calcd. for $C_{12}H_{11}F_3O_2$ 244.0706; Found: 244.0705.

3,3,3-Trifluoro-2-hydroxy-1-(3-phenylcyclobutyl)propan-1-one (3o)

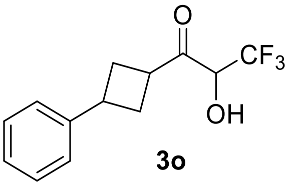

3o

The residue was purified by silica gel column chromatography (hexane/acetone = 15:1) to afford **3o** (76.4 mg, 74%) as a sticky yellowish oil. Product was isolated as a 7/2 mixture of two isomers. **^{1}H NMR** (400 MHz, $CDCl_3$) δ 7.38-7.31 (m, 2H), 7.27-7.21

(m, 3H), 4.65-4.52 (m, 1H), 4.16-4.07 (m, 1H), 3.78-3.52 (m, 2H), 2.85-2.63 (m, 2H), 2.57-2.43 (m, 2H). **^{19}F NMR** (376 MHz, $CDCl_3$) δ -73.99 (d, *J* = 7.6 Hz, 0.67F), -74.04 (d, *J* = 7.6 Hz, 2.33F). **^{13}C NMR** (101 MHz, $CDCl_3$) δ 204.7, 203.5, 144.3, 143.7, 128.5, 128.4, 126.5, 126.4, 126.3, 126.2, 122.51 (q, *J* = 283.0 Hz), 122.48 (q, *J* = 283.1 Hz), 73.7 (q, *J* = 31.8 Hz), 73.6 (q, *J* = 31.8 Hz), 39.0 (q, *J* = 2.2 Hz), 38.3 (q, *J* = 2.3 Hz), 36.0, 35.2, 32.7, 32.5, 31.6, 31.0. **IR (thin film)**: *ν* 3450, 2988, 2945, 1718, 1603, 1348, 1248, 1156, 755 cm^{-1}. **MS** (EI): *m/z* 258.1 [M^+]; **HRMS** (EI-TOF) *m/z*: [M^+] Calcd. for $C_{13}H_{13}F_3O_2$ 258.0862; Found: 258.0860.

1-(3-(Benzyloxy)cyclobutyl)-3,3,3-trifluoro-2-hydroxypropan-1-one (3p)

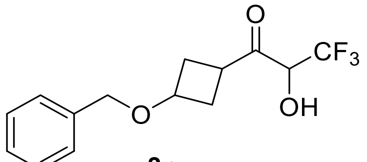

3p

The residue was purified by silica gel column chromatography (hexane/acetone = 15:1) to afford **3p** (86.5 mg, 75%) as a sticky yellowish oil. Product was isolated as a 2/1 mixture of two isomers. **^{1}H NMR** (400 MHz, $CDCl_3$) δ 7.39-7.27 (m, 5H), 4.57-4.47 (m, 1H), 4.44-4.43 (m, 2H), 4.31-3.99 (m, 2H), 3.62-2.98 (m, 1H), 2.61-2.45 (m, 2H), 2.43-2.23 (m, 2H). **^{19}F NMR** (376 MHz, $CDCl_3$) δ -73.96 (d, *J* = 7.8 Hz, 1F), -74.10 (d, *J* = 7.6 Hz, 2F). **^{13}C NMR** (101 MHz, $CDCl_3$) δ 205.0, 202.9, 137.73, 137.66, 128.43, 128.42, 127.82, 127.80, 122.4 (q, *J* = 283.1 Hz), 73.94 (q, *J* = 31.8 Hz), 73.79 (q, *J* = 31.7 Hz), 70.6, 70.4, 70.2, 67.9, 36.2 (q, *J* = 2.3 Hz), 33.90, 33.86, 33.6 (q, *J* = 2.2 Hz), 33.0, 32.6. **IR (thin film)**: *ν* 3441, 3032, 2945, 1722, 1496, 1455, 1357, 1248, 1127, 740, 699 cm^{-1}. **MS** (FI): *m/z* 288.1 [M^+]; **HRMS** (FI-TOF) *m/z*: [M^+] Calcd. for $C_{14}H_{15}F_3O_3$ 288.0968; Found: 288.0965.

1-(2,3-Dihydro-1*H*-inden-2-yl)-3,3,3-trifluoro-2-hydroxypropan-1-one (3q)

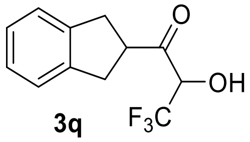

3q

The residue was purified by silica gel column chromatography (hexane/acetone = 15:1) to afford **3q** (63.5 mg, 65%) as a white solid. **M.P.** 60.9-61.7 °C. **^{1}H NMR** (400 MHz, $CDCl_3$) δ 7.26-7.18 (m, 4H), 4.73 (q, *J* = 7.7 Hz, 1H), 4.07 (s, 1H), 3.85-3.76 (m, 1H), 3.44-3.34 (m, 2H), 3.20-3.12 (m, 2H). **^{19}F NMR** (376 MHz, $CDCl_3$) δ -73.78 (d, *J* = 7.5 Hz, 3F). **^{13}C NMR** (101 MHz, $CDCl_3$) δ 204.5, 140.9, 139.7, 127.2, 126.9, 124.5, 124.3, 122.5 (q, *J* = 283.4 Hz), 74.1 (q, *J* = 31.5 Hz), 48.0 (q, *J* = 2.1 Hz), 36.7, 34.8. **IR (thin film)**: *ν* 3408, 2930, 2854, 1723, 1488, 1326, 1261, 1124, 1002, 758, 528 cm^{-1}. **MS** (EI): *m/z* 244.1 [M^+]; **HRMS** (EI-TOF) *m/z*: [M^+] Calcd. for $C_{12}H_{11}F_3O_2$ 244.0706; Found: 244.0703.

3,3,3-Trifluoro-2-hydroxy-1-(1,2,3,4-tetrahydronaphthalen-2-yl)propan-1-one (3r)

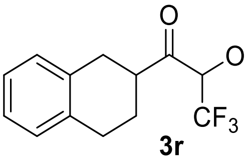

3r

The residue was purified by silica gel column chromatography (hexane/acetone = 15:1) to afford **3r** (56.8 mg, 55%) as a sticky yellowish oil. Product was isolated as a 1:1 mixture of two isomers. **^{1}H NMR** (400 MHz, $CDCl_3$) δ 7.20-7.08 (m, 4H), 4.84-4.71 (m, 1H), 4.12-4.08 (m, 1H), 3.24-2.84 (m, 5H), 2.25-2.12 (m, 1H), 1.97-1.77 (m, 1H). **^{19}F NMR** (376 MHz, $CDCl_3$) δ -73.74 (d, *J* = 7.5 Hz, 1.5F), -73.77 (d, *J* = 7.6 Hz, 1.5F). **^{13}C NMR** (101 MHz, $CDCl_3$) δ 205.7, 205.6, 135.5, 134.7, 134.1, 133.5, 129.1, 129.04, 128.85, 128.81, 126.5, 126.21, 126.20, 126.1, 122.55 (q, *J* = 283.3 Hz), 122.52 (q, *J* = 283.6 Hz), 73.74 (q, *J* = 31.5 Hz), 73.24 (q, *J* = 31.4 Hz), 44.4-44.2 (m), 32.4, 30.0, 28.6, 28.0, 26.5, 24.4. **IR (thin film)**: *v* 3454, 3063, 2932, 1722, 1497, 1454, 1247, 1180, 747, 434 cm^{-1}. **MS** (EI): *m/z* 258.1 [M^+]; **HRMS** (EI-TOF) *m/z*: [M^+] Calcd. for $C_{13}H_{13}F_3O_2$ 258.0862; Found: 258.0868.

1-(4-(4-Chlorophenyl)cyclohexyl)-3,3,3-trifluoro-2-hydroxypropan-1-one (3s)

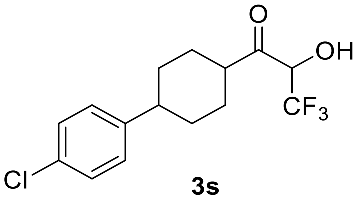

3s

The residue was purified by silica gel column chromatography (hexane/acetone = 15:1) to afford **3s** (89.8 mg, 70%) as a white solid. **M.P.** 127.7-129.0 °C. **^{1}H NMR** (400 MHz, $CDCl_3$) δ 7.28-7.23 (m, 2H), 7.14-7.09 (m, 2H), 4.72-4.61 (m, 1H), 4.04-3.99 (m, 1H), 2.84-2.76 (m, 1H), 2.57-2.48 (m, 1H), 2.13-1.94 (m, 4H), 1.77-1.40 (m, 4H). **^{19}F NMR** (376 MHz, $CDCl_3$) δ -73.72 (d, *J* = 7.5 Hz, 3F). **^{13}C NMR** (101 MHz, $CDCl_3$) δ 206.0, 144.6, 131.9, 128.5, 128.0, 122.5 (q, *J* = 283.5 Hz), 73.3 (q, *J* = 31.3 Hz), 47.0 (q, *J* = 2.1 Hz), 42.7, 33.3, 32.4, 29.8, 27.3. **IR (thin film)**: *v* 3368, 2942, 2864, 1724, 1492, 1451, 1330, 1262, 822, 529 cm^{-1}. **MS** (EI): *m/z* 320.1 [M^+]; **HRMS** (EI-TOF) *m/z*: [M^+] Calcd. for $C_{15}H_{16}ClF_3O_2$ 320.0785; Found: 320.0783.

6-(4-(Bis(2-chloroethyl)amino)phenyl)-1,1,1-trifluoro-2-hydroxyhexan-3-one (3t)

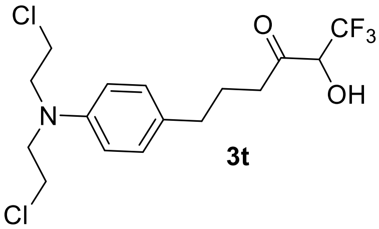

3t

The residue was purified by silica gel column chromatography (hexane/acetone = 15:1–10:1) to afford **3t** (100.4 mg, 65%) as a sticky yellowish oil. **^{1}H NMR** (400 MHz, $CDCl_3$) δ 7.06 (d, *J* = 8.6 Hz, 2H), 6.64 (d, *J* = 8.7 Hz, 2H), 4.52-4.40 (m, 1H), 4.03 (d, *J* = 6.3 Hz, 1H), 3.75-3.68 (m, 4H), 3.67-3.60 (m, 4H), 2.79-2.49 (m, 4H), 2.05-1.93 (m, 2H). **^{19}F NMR** (376 MHz, $CDCl_3$) δ -74.08 (d, *J* = 7.9 Hz, 3F). **^{13}C NMR** (101 MHz, $CDCl_3$) δ 202.7, 144.5, 129.8, 129.6, 122.4 (q, *J* = 283.3 Hz), 112.2, 74.8 (q, *J* = 31.6 Hz), 53.5, 40.5, 38.8 (q, *J* = 2.3 Hz), 33.5, 24.9. **IR (thin film)**: *v* 3451, 2930, 1727, 1615, 1519, 1355, 1248, 1181, 1015, 742 cm^{-1}. **MS** (ESI): *m/z* 386.1 $[M+H]^+$; **HRMS** (ESI-TOF) *m/z*: $[M+H]^+$ Calcd. for $C_{16}H_{21}Cl_2F_3NO_2$ 386.0896; Found: 386.0895.

(8*R*,9*S*,10*S*,13*R*,14*S*,17*R*)-10,13-Dimethyl-17-((2*R*)-7,7,7-trifluoro-6-hydroxy-5-oxoheptan-2-yl)dodecahydro-3*H*-cyclopenta[*a*]phenanthrene-3,7,12(2*H*,4*H*)-trione (3u)

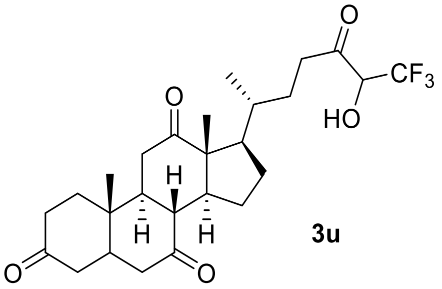

3u

The residue was purified by silica gel column chromatography (hexane/acetone = 10:1–5:1) to afford **3u** (135.7 mg, 70%) as a white solid. Product was isolated as a 1:1 mixture of two isomers. **M.P.** 207.9-208.5 °C. **^{1}H NMR** (400 MHz, $CDCl_3$) δ 4.56-4.44 (m, 1H), 4.19-4.12 (m, 1H), 2.95-2.77 (m, 3H), 2.74-2.53 (m, 2H), 2.38-2.07 (m, 8H), 2.03-1.78 (m, 6H), 1.66-1.53 (m, 1H), 1.48-1.40 (m, 1H), 1.38 (s, 3H), 1.32-1.18 (m, 3H), 1.04 (s, 3H), 0.86-0.77 (m, 3H). **^{19}F NMR** (376 MHz, $CDCl_3$) δ -74.08 (d, *J* = 7.6 Hz, 1.5F), -74.13 (d, *J* = 7.5 Hz, 1.5F). **^{13}C NMR** (101 MHz, $CDCl_3$) δ 211.92, 211.89, 209.1, 208.6, 203.3, 203.2, 122.4 (q, *J* = 283.3 Hz), 74.85 (q, *J* = 31.4 Hz), 74.58 (q, *J* = 31.3 Hz), 56.8, 51.70, 51.67, 48.9, 46.7, 45.5, 45.4, 45.3, 44.9, 42.7, 38.5, 37.0-36.7 (m), 36.7-36.4 (m), 36.4, 35.9, 35.3, 35.16, 35.15, 28.7, 28.6, 27.6, 27.5, 25.0, 21.8, 18.6, 18.5, 11.73, 11.71. **IR (thin film)**: *v* 3446, 2967, 1712, 1468, 1273, 1173, 1125, 912, 733 cm^{-1}. **MS** (ESI): *m/z* 485.3 $[M+H]^+$; **HRMS** (ESI-TOF) *m/z*: $[M+H]^+$ Calcd. for $C_{26}H_{36}F_3O_5$ 485.2509; Found: 485.2507.

4. Scale-up reaction

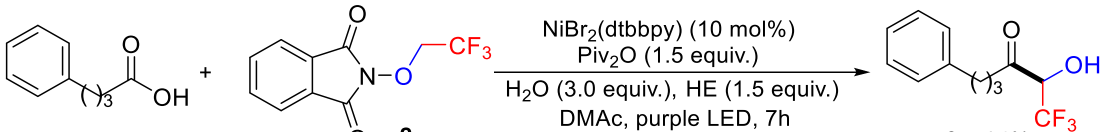

1a (1.0 mmol) **2** **3a**, 64%

In the glove box with nitrogen atmosphere, to a 25 mL vial equipped with a magnetic stir bar, NiBr2(dtbbpy) (48.9 mg, 0.1 mmol, 10 mol %), 4-phenylbutyric acid **1a** (1.0 mmol, 1.0 equiv, 164.2 mg), **2** (367.5 mg, 1.5 mmol, 1.5 equiv), Hantzsch ester (379.9 mg, 1.5 mmol, 1.5 equiv), and 10.0 mL of anhydrous *N,N*-dimethylacetamide were added, the vial was then re-capped and taken out of the glove box, and Piv_2O (279.3 mg, 1.5 mmol, 1.5 equiv), H_2O (54.0 mg, 3.0 mmol, 3.0 equiv) were added. The vial was sealed with parafilm and the reaction mixture was then stirred under irradiation by purple LEDs (λ_{max} = 399 nm) for 7 h. After the reaction was complete, the reaction mixture was poured into water and extracted with EtOAc. The combined organic phase was separated and washed with brine, dried over Na_2SO_4, and concentrated under vacuum. The resulting residue was purified by silica gel flash column chromatography (hexane/acetone = 15:1) to afford **3a** (157.4 mg, 64%) as a white solid.

5. ORTEP Drawing of the X-ray crystallographic structure

5.1 1,1,1-Trifluoro-2-hydroxy-6-phenylhexan-3-one (3a)

The crystals were obtained from a solution of chloroform and hexane upon slow volatilization.

The crystal structure has been deposited at the Cambridge Crystallographic Date Center and allocated the deposition numbers CCDC 2222184. The thermal ellipsoids are shown at the 30% probability level. This data can be obtained free of charge from the Cambridge Crystallographic Date Center via www.ccdc.cam.ac.uk/data_request/cif

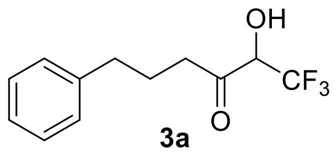

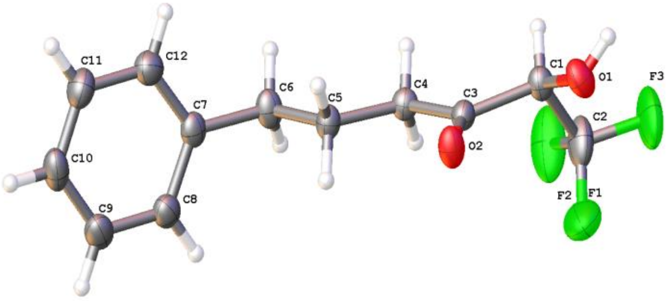

Crystal data and structure refinement for 3a.

Identification code	mj22514_0m	
Empirical formula	C12 H13 F3 O2	
Formula weight	246.22	
Temperature	224.00 K	
Wavelength	1.34139 Å	
Crystal system	Orthorhombic	
Space group	$P2_12_12_1$	
Unit cell dimensions	a = 5.5450(2) Å	a= 90°.
	b = 9.2825(3) Å	b= 90°.
	c = 22.9476(6) Å	g = 90°.
Volume	1181.15(7) $Å^3$	
Z	4	
Density (calculated)	1.385 Mg/ m^3	
Absorption coefficient	0.688 mm^{-1}	
F(000)	512	
Crystal size	0.07 x 0.07 x 0.05 mm^3	
Theta range for data collection	4.470 to 54.923°.	
Index ranges	-6<=h<=6, -11<=k<=7, -28<=l<=19	
Reflections collected	6899	
Independent reflections	2226 [R(int) = 0.0549]	
Completeness to theta = 53.594°	99.4 %	
Absorption correction	Semi-empirical from equivalents	
Max. and min. transmission	0.7508 and 0.6091	
Refinement method	Full-matrix least-squares on F^2	
Data / restraints / parameters	2226 / 0 / 155	
Goodness-of-fit on F^2	1.063	
Final R indices [I>2sigma(I)]	R1 = 0.0528, wR2 = 0.1368	
R indices (all data)	R1 = 0.0737, wR2 = 0.1492	
Absolute structure parameter	0.5(2)	
Extinction coefficient	n/a	
Largest diff. peak and hole	0.215 and -0.198 e.$Å^{-3}$	

5.2 1-(4-(4-Chlorophenyl)cyclohexyl)-3,3,3-trifluoro-2-hydroxypropan-1-one (3s)

The crystals were obtained from a solution of chloroform and hexane upon slow volatilization.

The crystal structure has been deposited at the Cambridge Crystallographic Date Center and allocated the deposition numbers CCDC 2234475. The thermal ellipsoids are shown at the 30% probability level. This data can be obtained free of charge from the Cambridge Crystallographic Date Center via www.ccdc.cam.ac.uk/data_request/cif

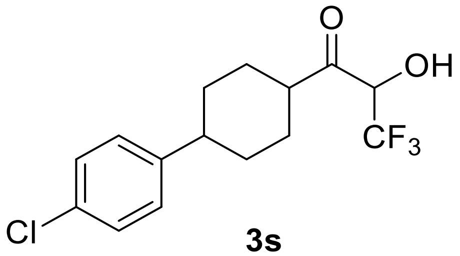

3s

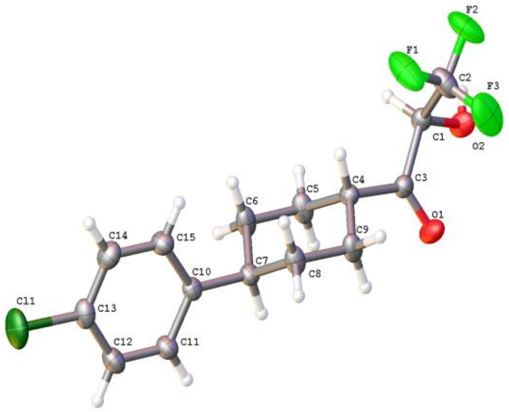

Crystal data and structure refinement for 3s.

Identification code	mj23005_0m	
Empirical formula	C15 H16 Cl F3 O2	
Formula weight	320.73	
Temperature	227.00 K	
Wavelength	1.34139 Å	
Crystal system	Monoclinic	
Space group	P 1 21/c 1	
Unit cell dimensions	a = 13.6886(4) Å	a= 90°.
	b = 5.39160(10) Å	b= 102.613(2)°.
	c = 20.7714(5) Å	g = 90°.
Volume	1496.01(6) $Å^3$	
Z	4	
Density (calculated)	1.424 Mg/ m^3	
Absorption coefficient	1.703 mm^{-1}	
F(000)	664	
Crystal size	0.07 x 0.07 x 0.05 mm^3	
Theta range for data collection	3.794 to 54.929°.	
Index ranges	-15<=h<=16, -6<=k<=6, -25<=l<=22	
Reflections collected	11365	
Independent reflections	2819 [R(int) = 0.0653]	
Completeness to theta = 53.594°	98.9 %	
Absorption correction	Semi-empirical from equivalents	
Max. and min. transmission	0.7508 and 0.4109	
Refinement method	Full-matrix least-squares on F^2	
Data / restraints / parameters	2819 / 0 / 191	
Goodness-of-fit on F^2	1.071	
Final R indices [I>2sigma(I)]	R1 = 0.0580, wR2 = 0.1589	
R indices (all data)	R1 = 0.0777, wR2 = 0.1747	
Extinction coefficient	n/a	
Largest diff. peak and hole	0.305 and -0.297 e.$Å^{-3}$	

6. References

1. Shu, C.; Noble, A.; Aggarwal, V. K. *Nature* **2020**, *586*, 714-719.

7. Copies of ^{1}H NMR, ^{19}F NMR and ^{13}C NMR spectra of products

^{1}H NMR (400 MHz, CDCl$_3$)

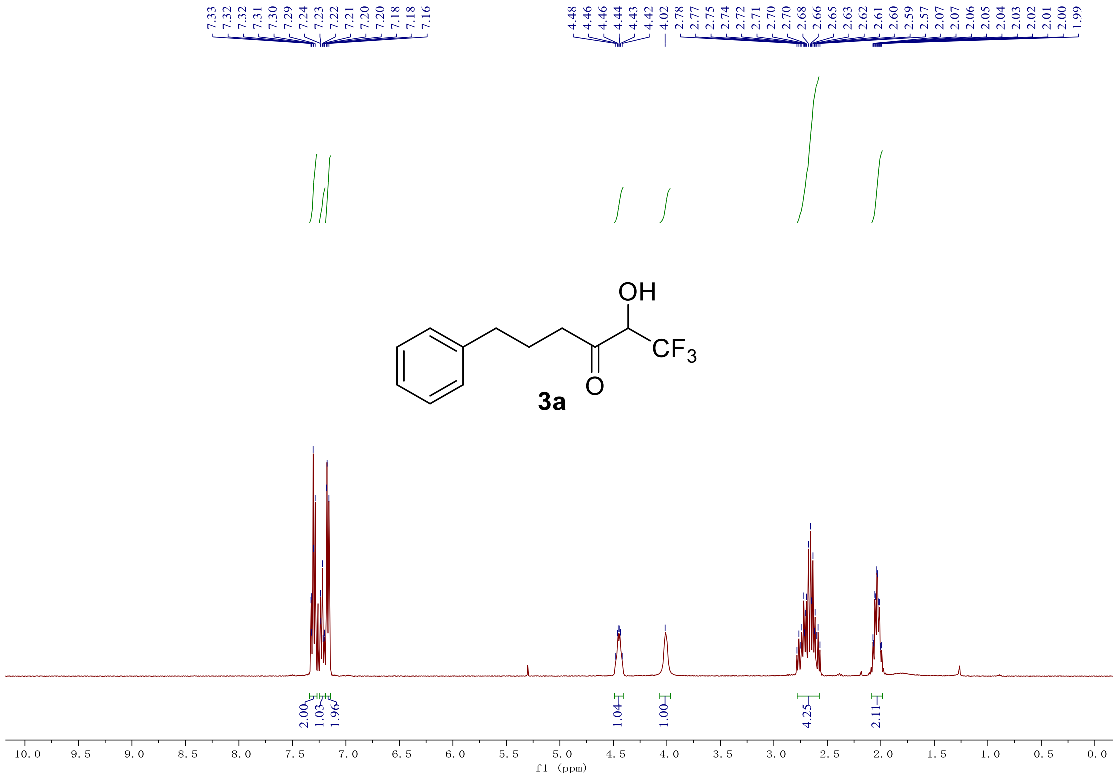

^{19}F NMR (376 MHz, CDCl$_3$)

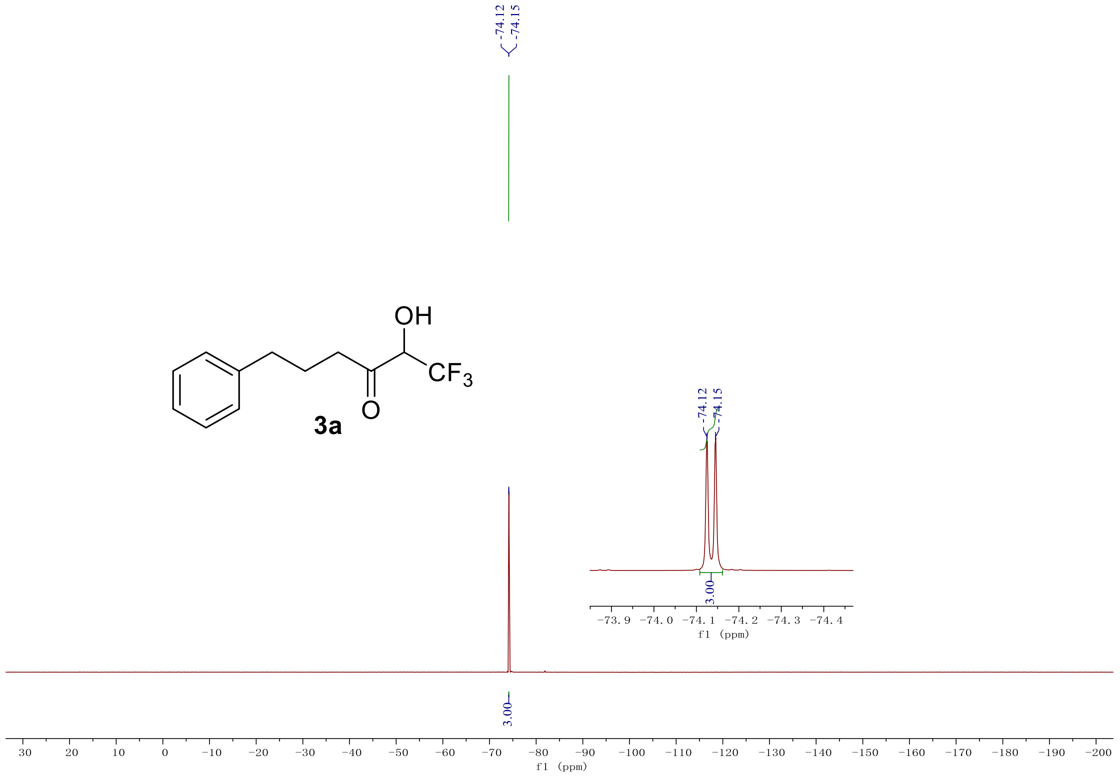

^{13}C NMR (101 MHz, CDCl$_3$)

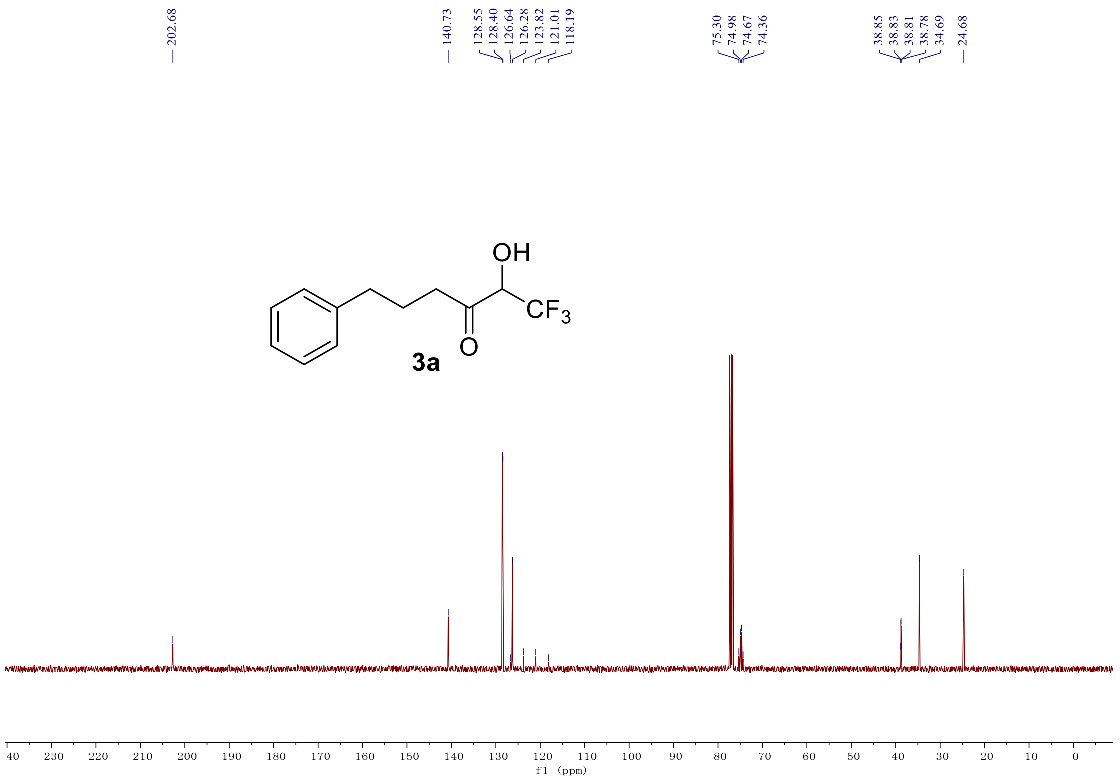

^{1}H NMR (400 MHz, CDCl$_3$)

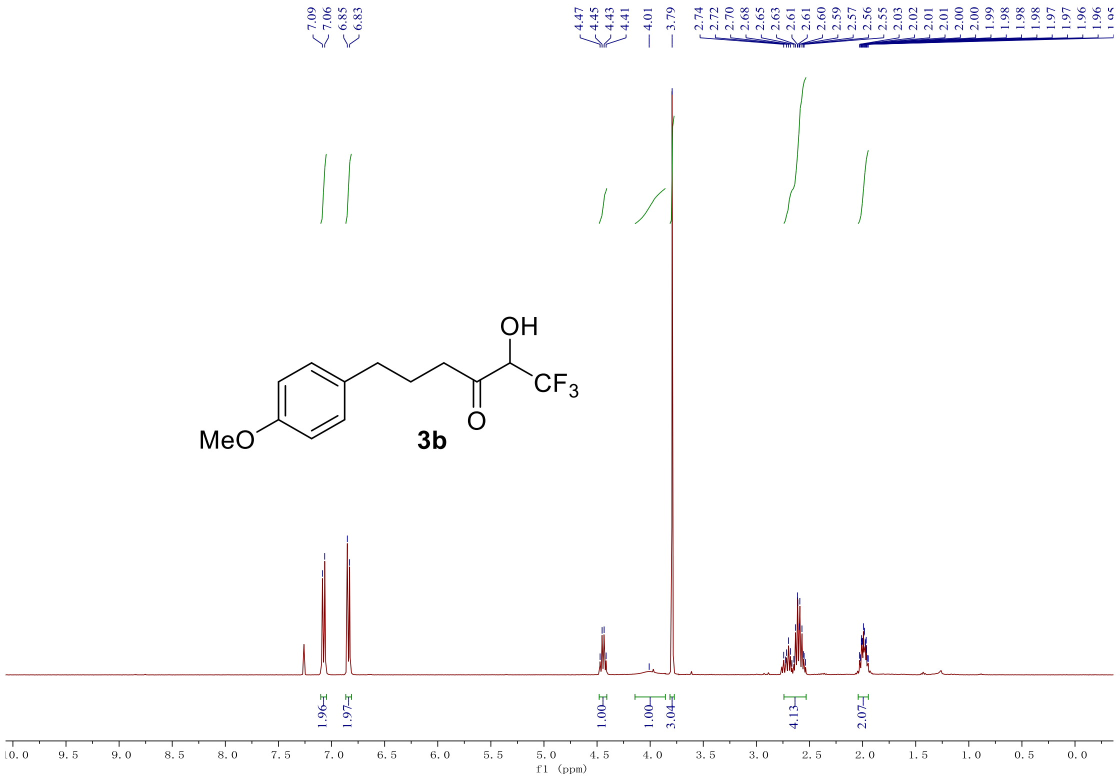

^{19}F NMR (376 MHz, $CDCl_3$)

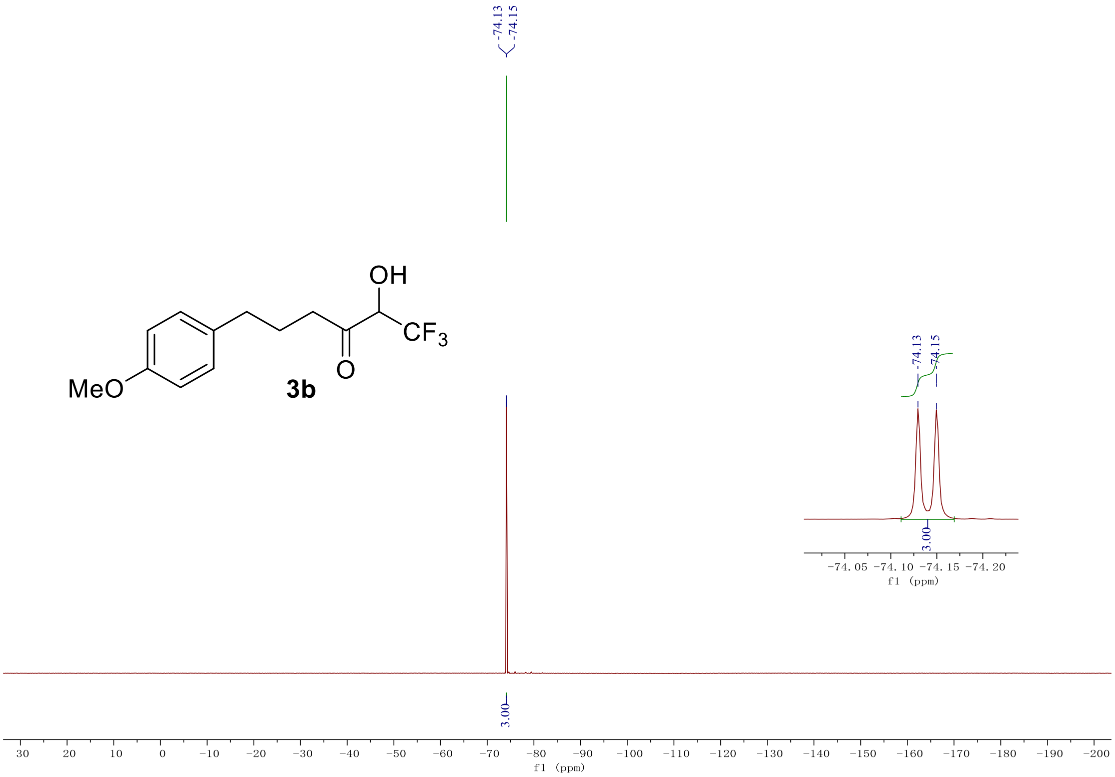

^{13}C NMR (101 MHz, $CDCl_3$)

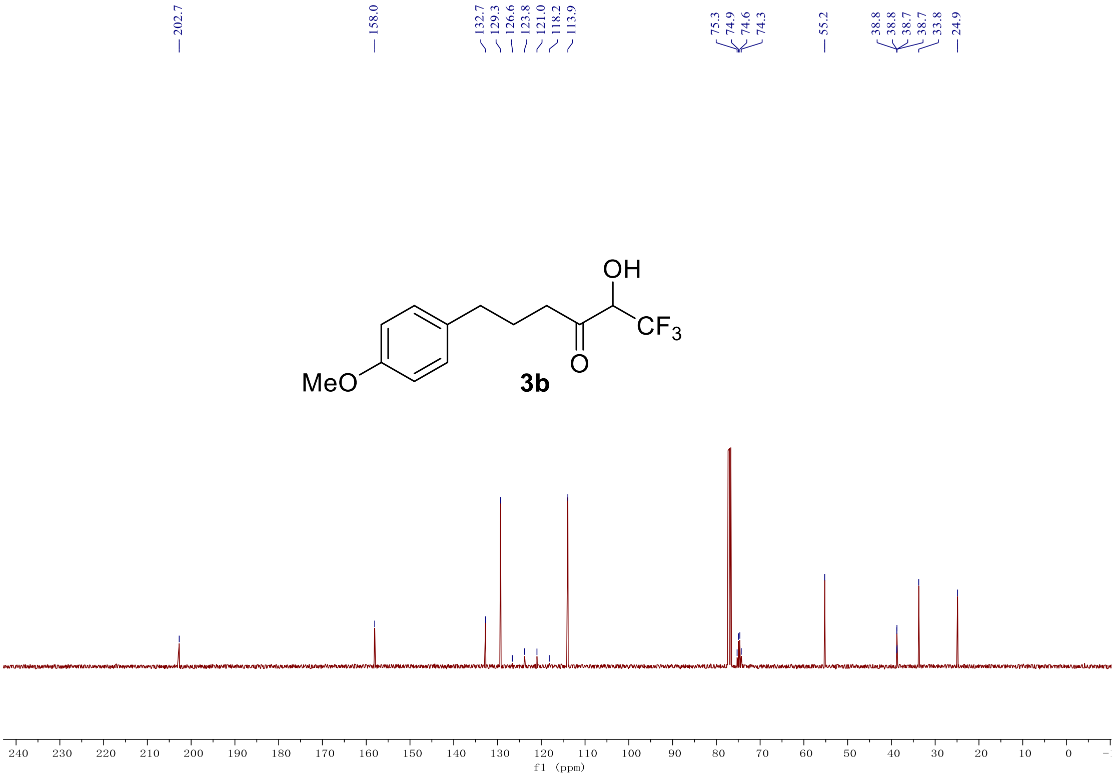

^{1}H NMR (400 MHz, $CDCl_3$)

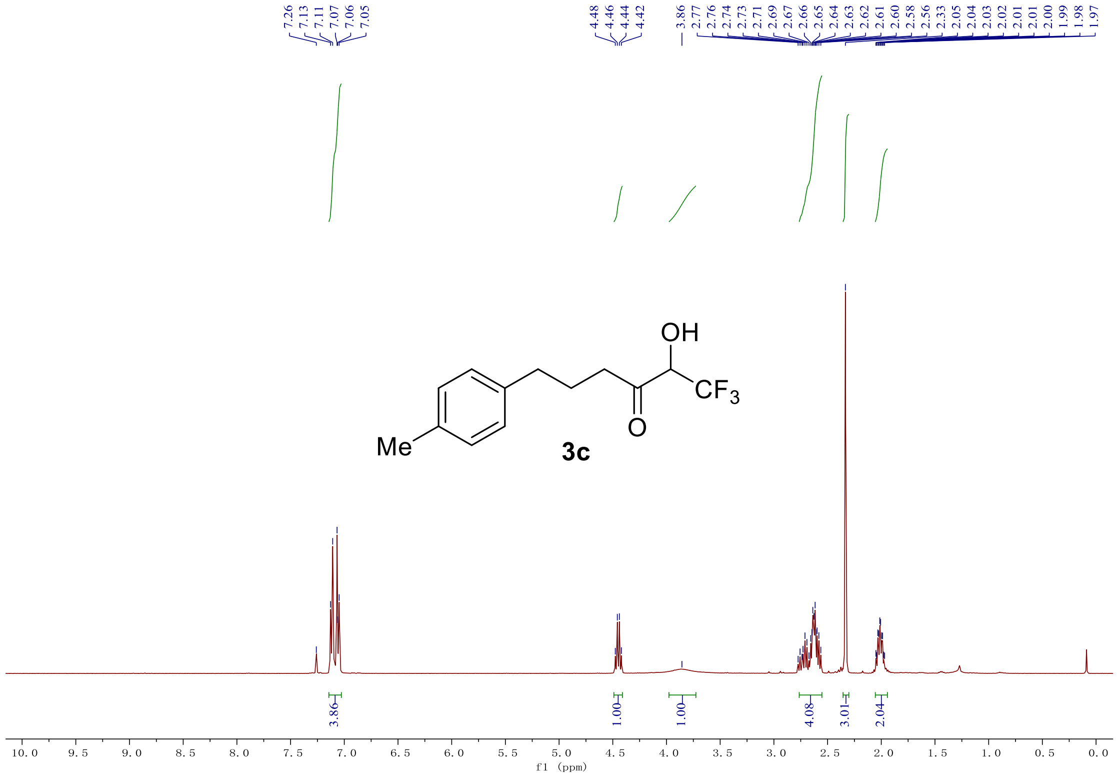

^{19}F NMR (376 MHz, $CDCl_3$)

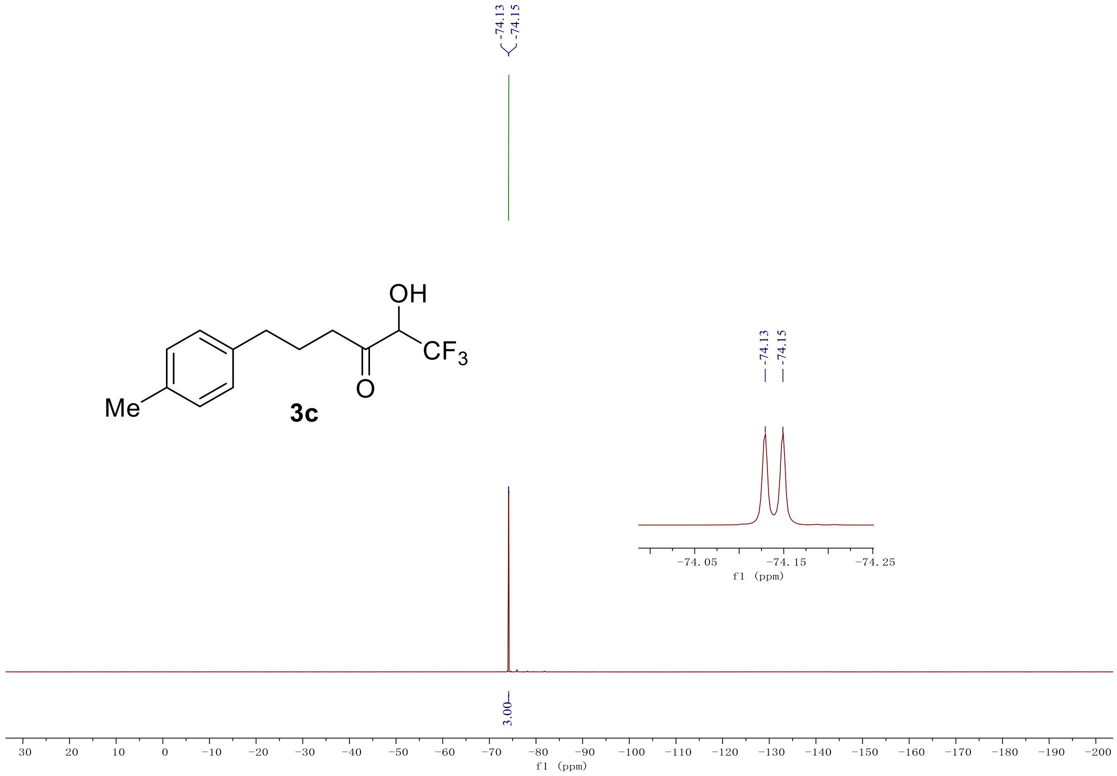

^{13}C NMR (101 MHz, $CDCl_3$)

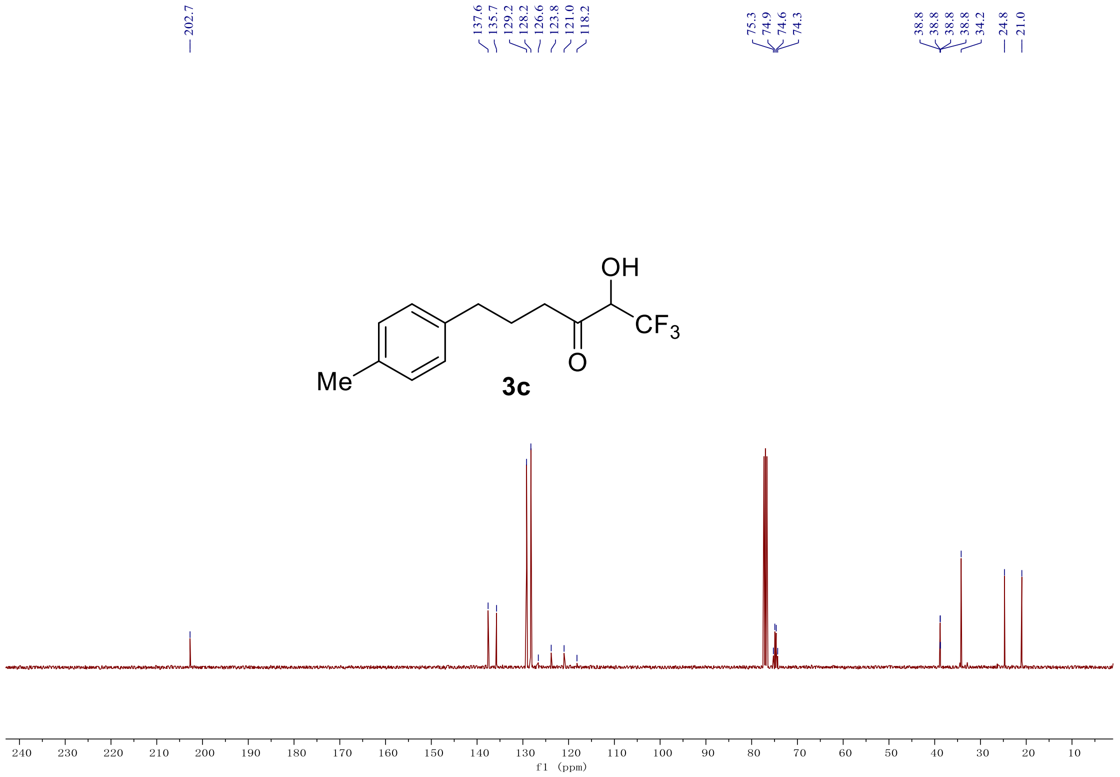

^{1}H NMR (400 MHz, $CDCl_3$)

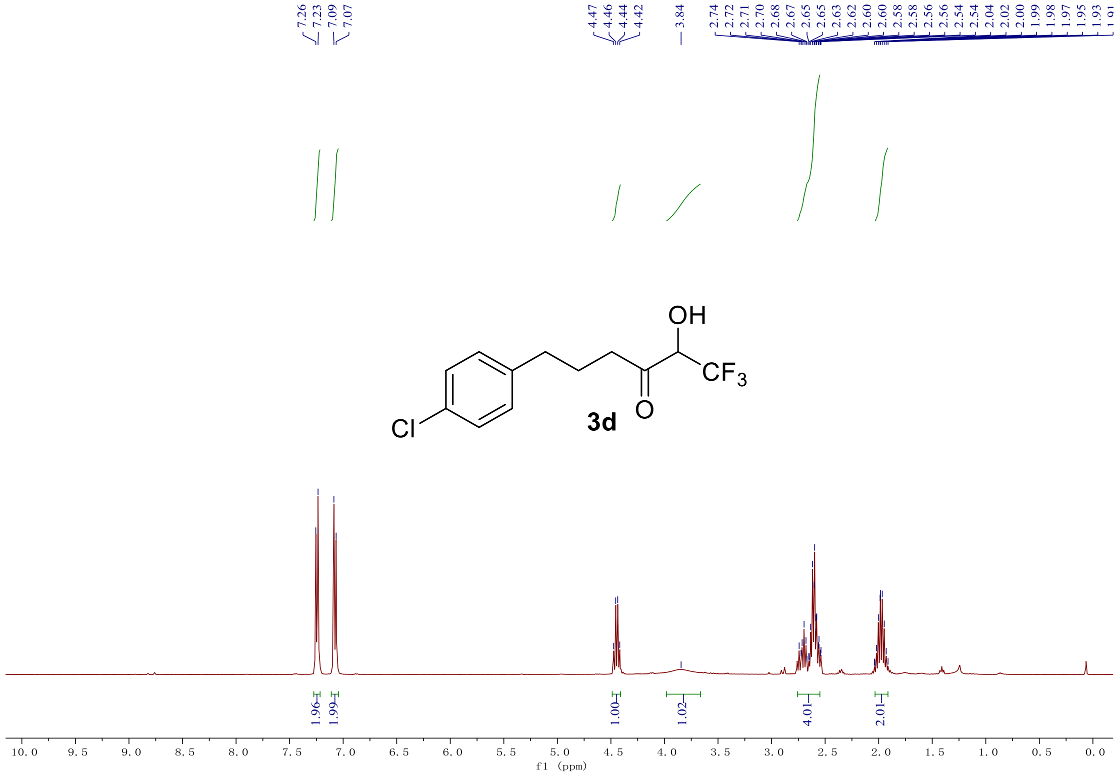

^{19}F NMR (376 MHz, $CDCl_3$)

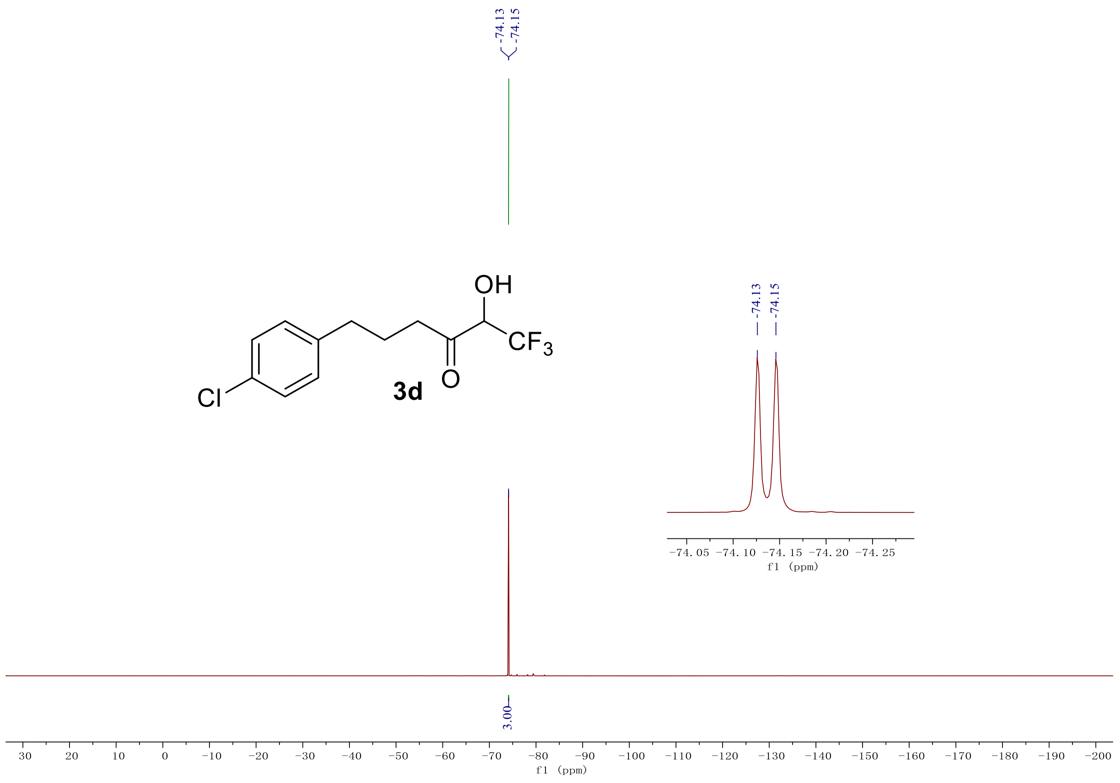

^{13}C NMR (101 MHz, $CDCl_3$)

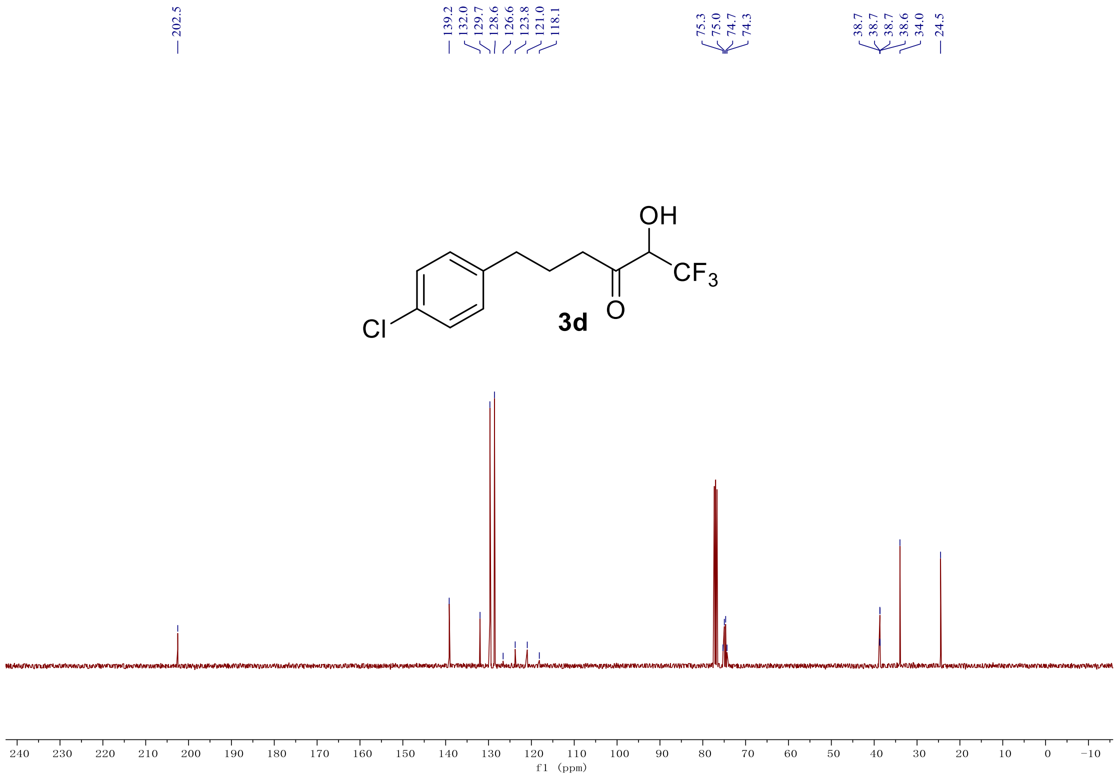

^{1}H NMR (400 MHz, $CDCl_3$)

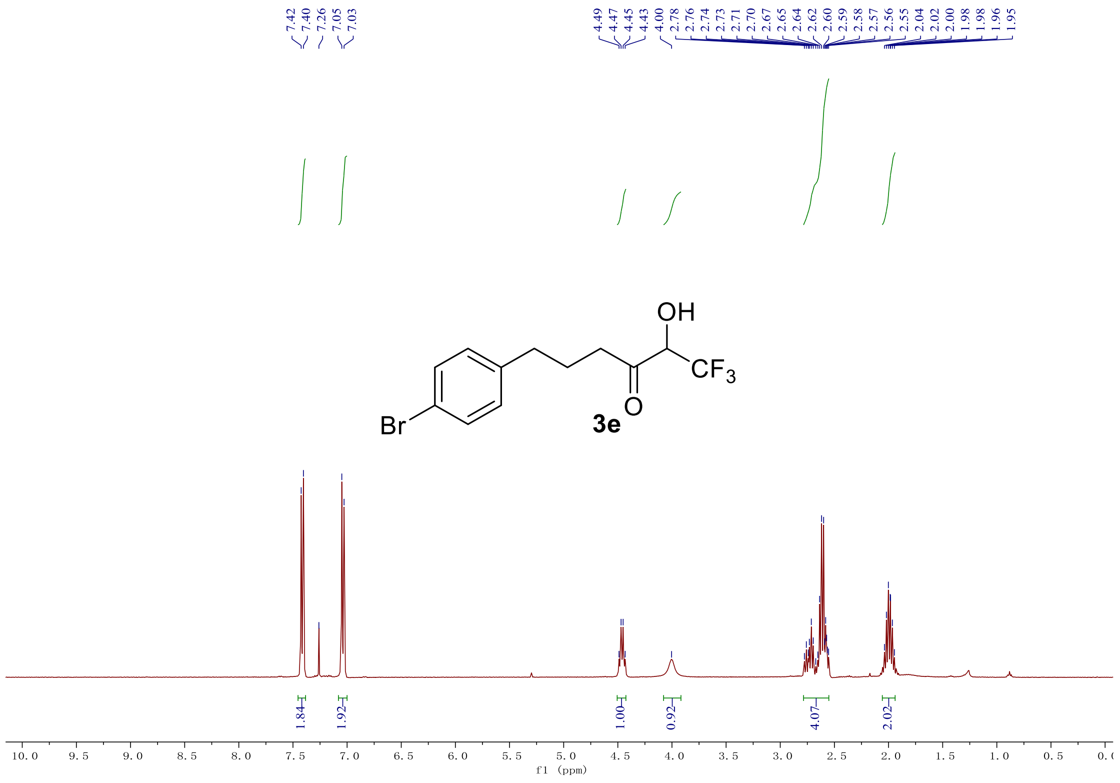

^{19}F NMR (376 MHz, $CDCl_3$)

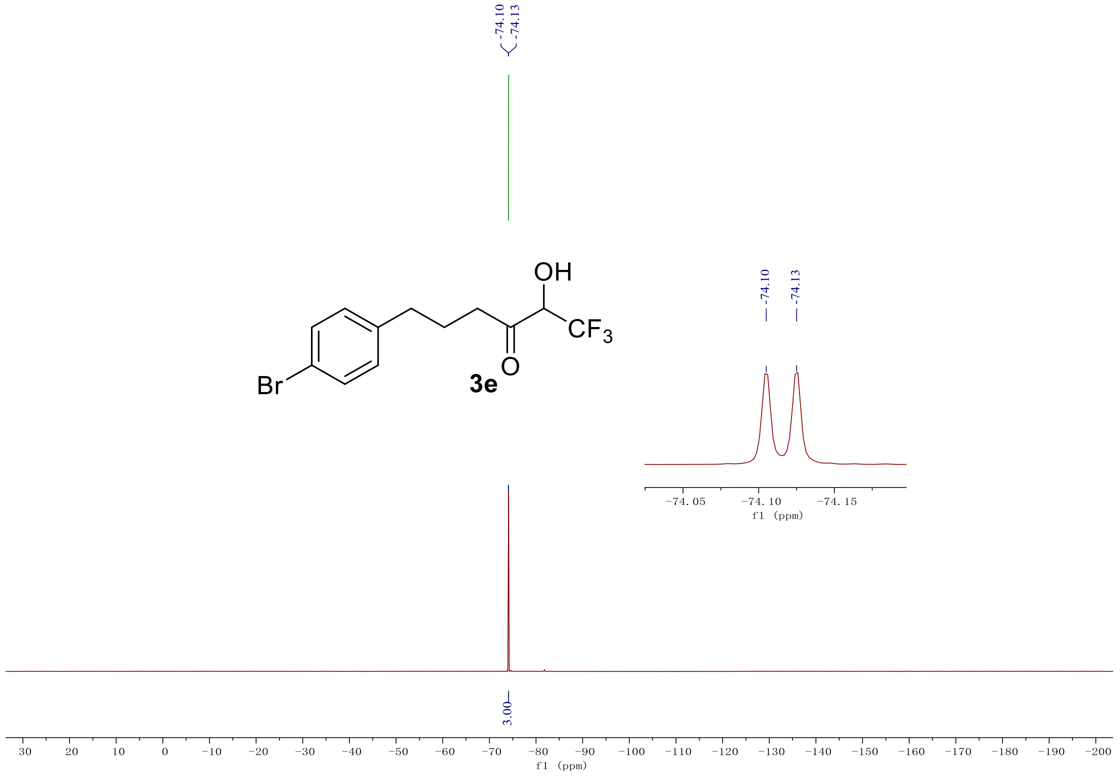

^{13}C NMR (101 MHz, CDCl$_3$)

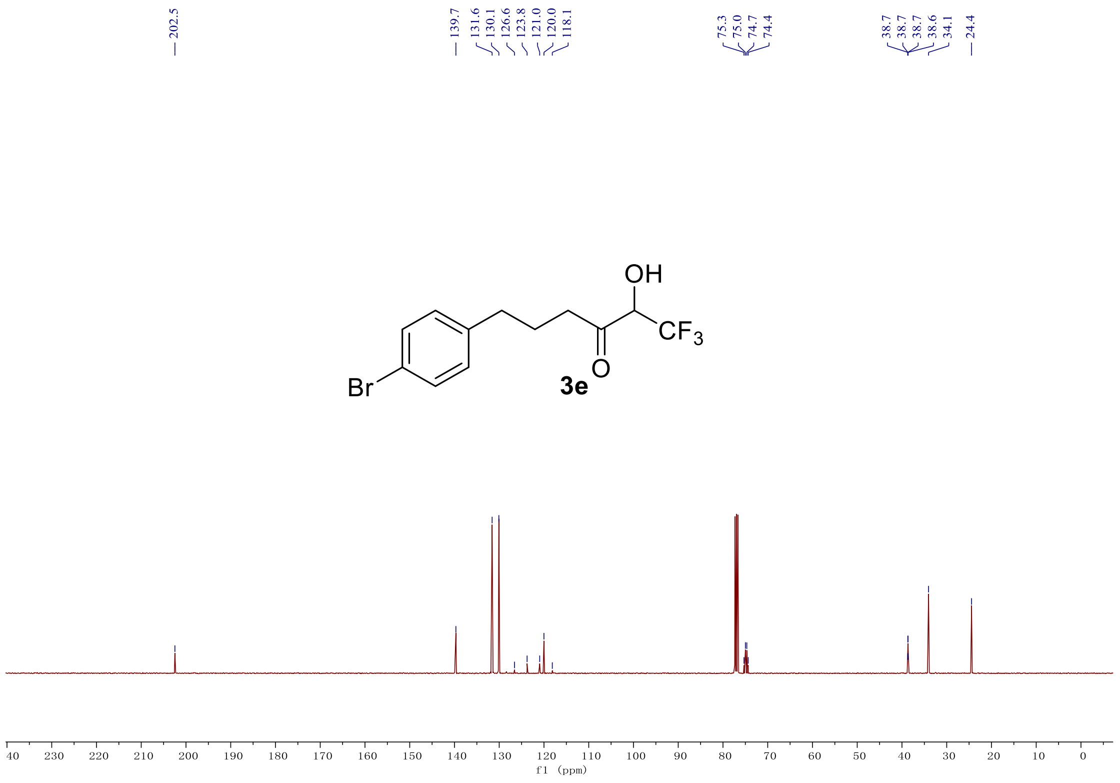

^{1}H NMR (400 MHz, CDCl$_3$)

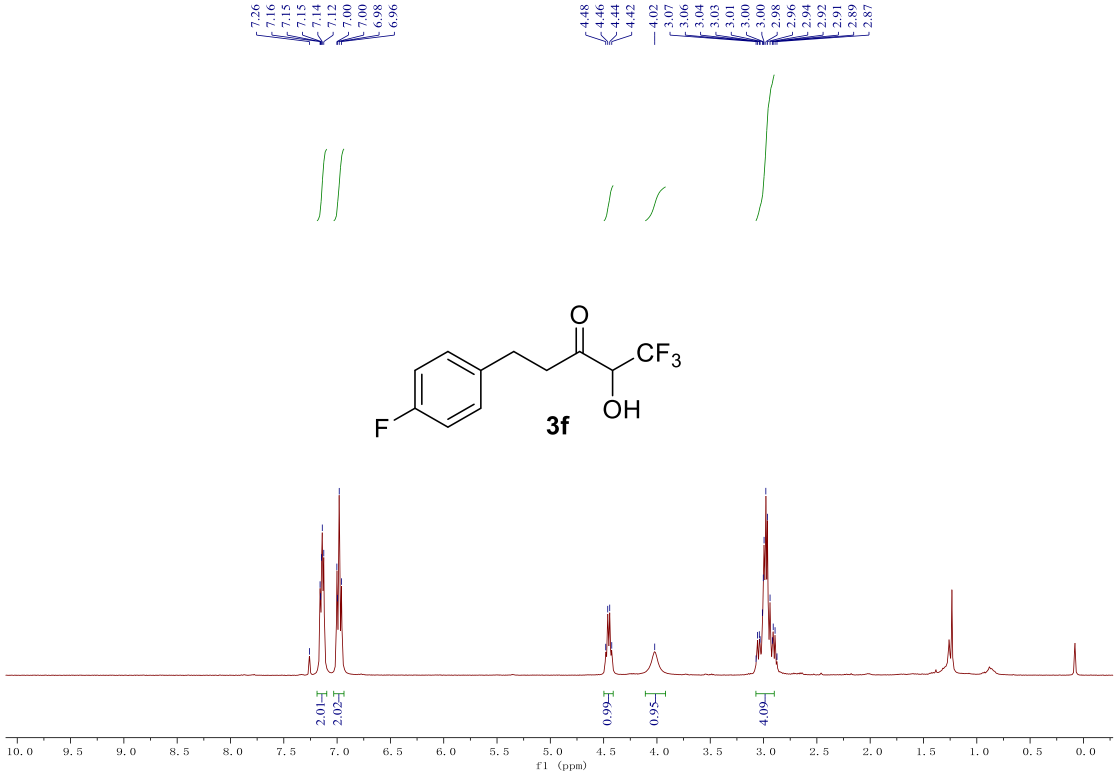

^{19}F NMR (376 MHz, $CDCl_3$)

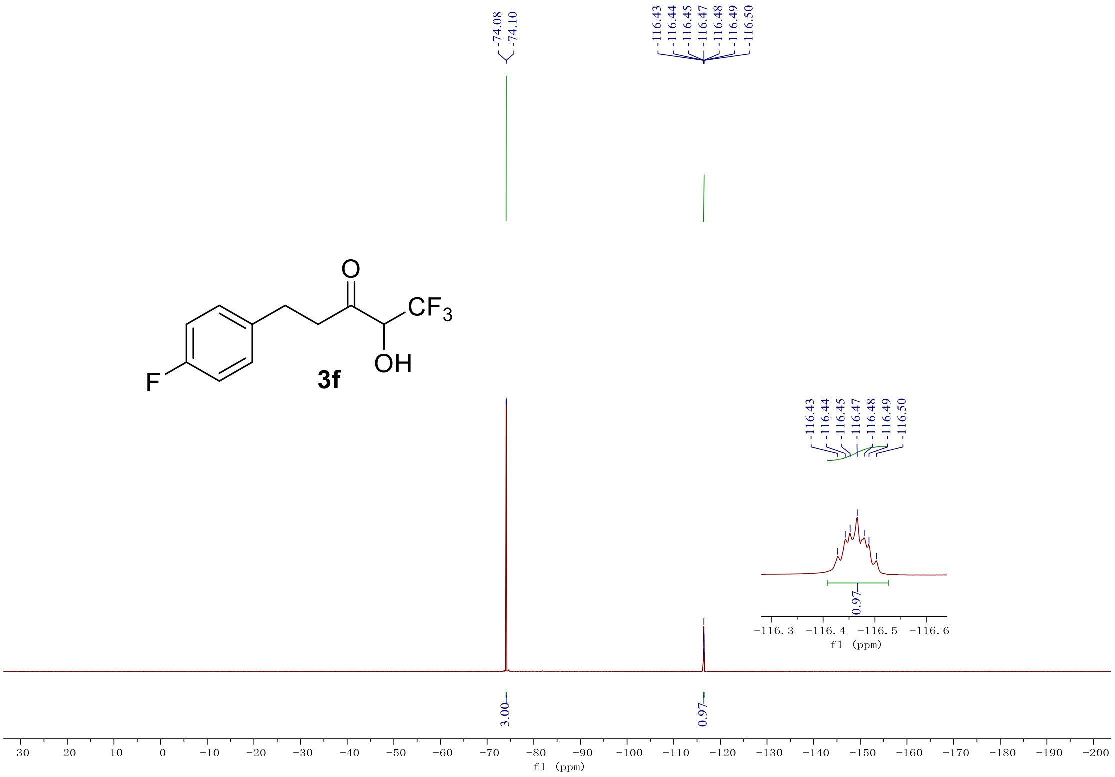

^{13}C NMR (101 MHz, $CDCl_3$)

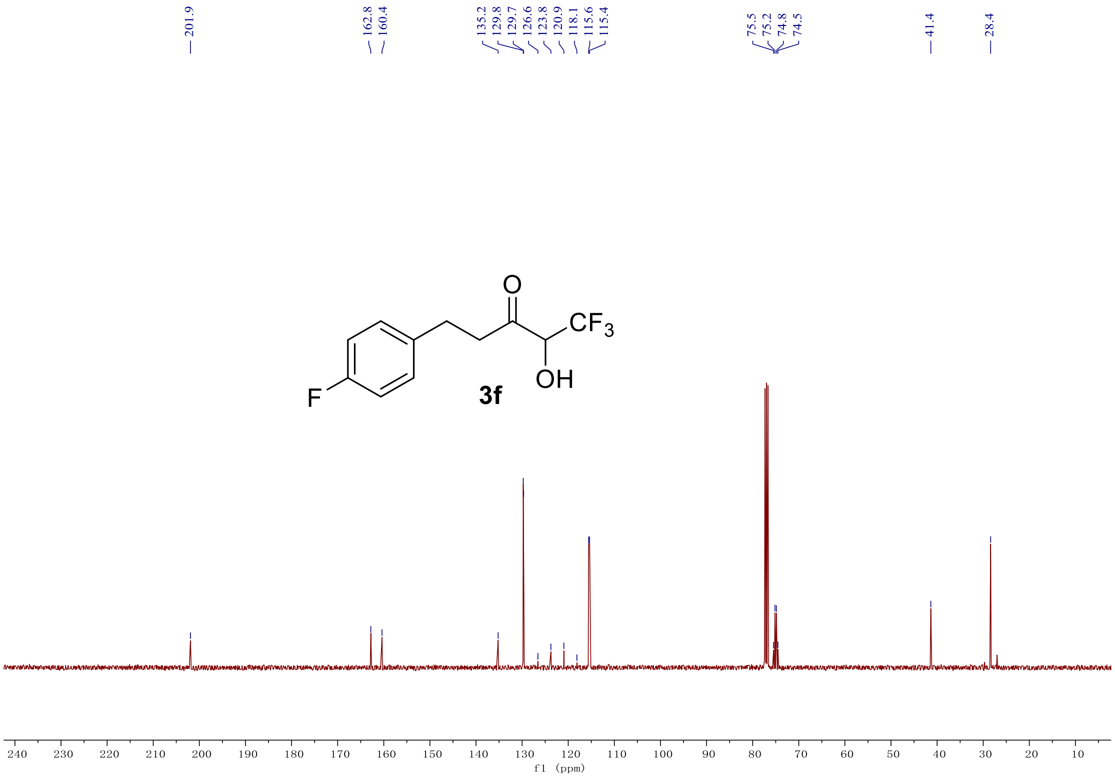

^1H NMR (400 MHz, $CDCl_3$)

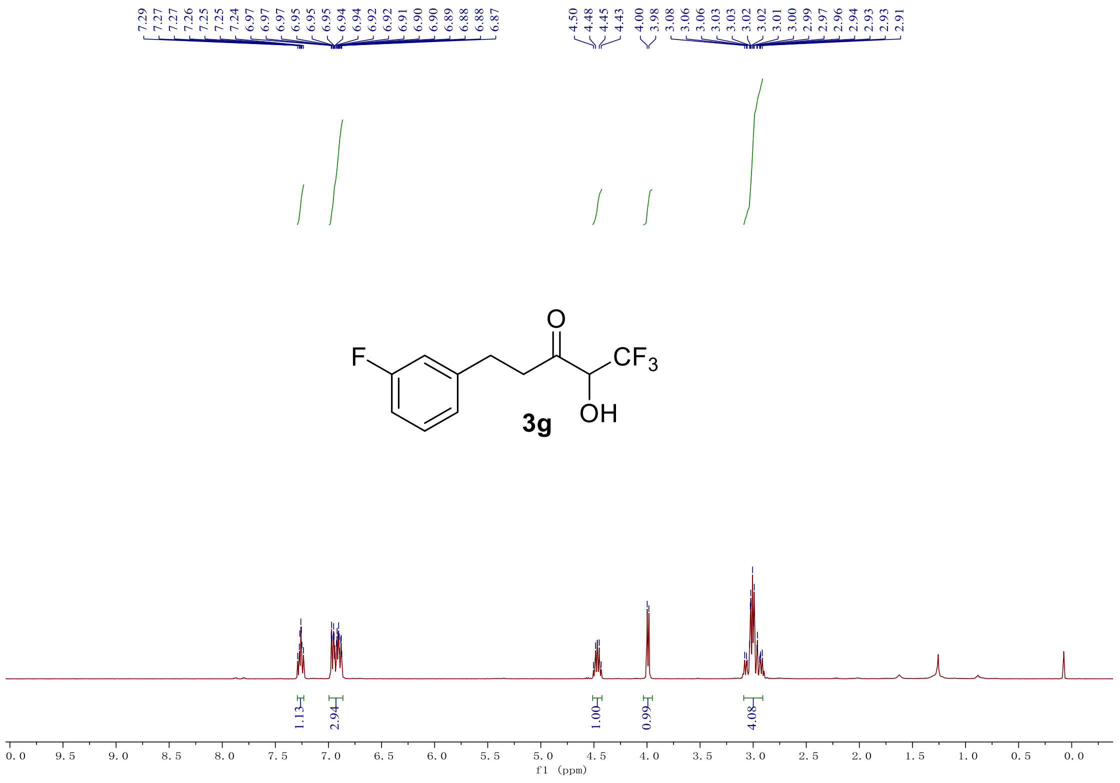

^{19}F NMR (376 MHz, $CDCl_3$)

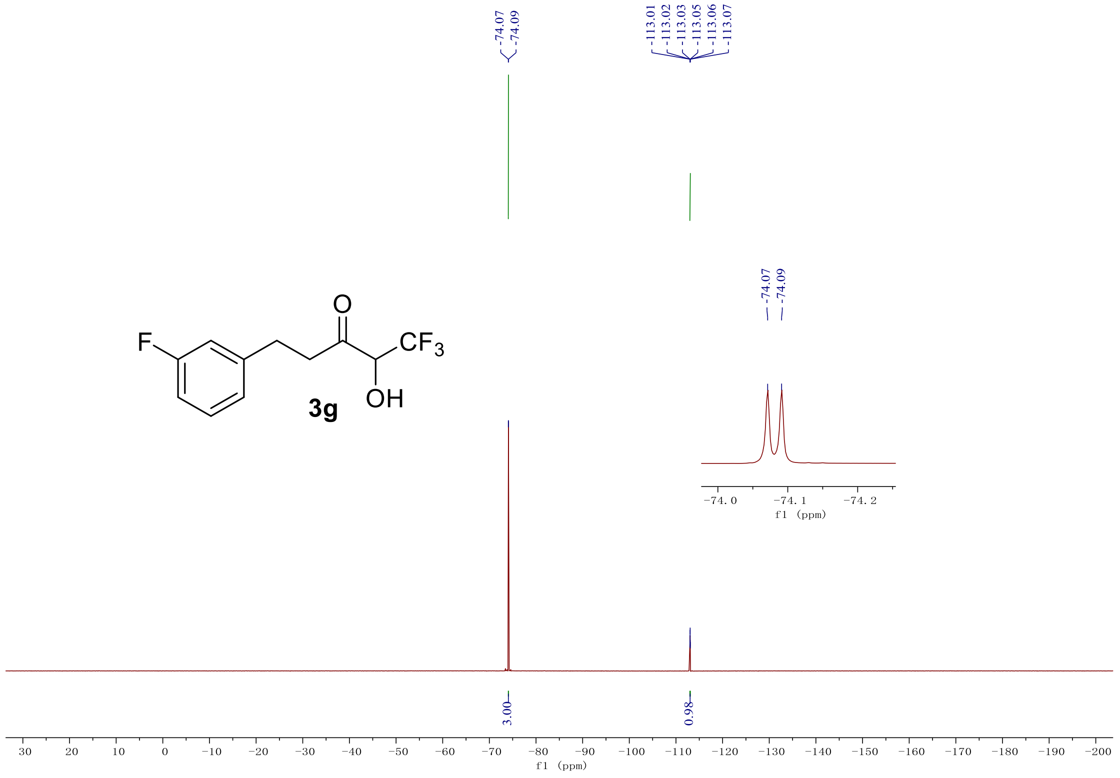

^{13}C NMR (101 MHz, $CDCl_3$)

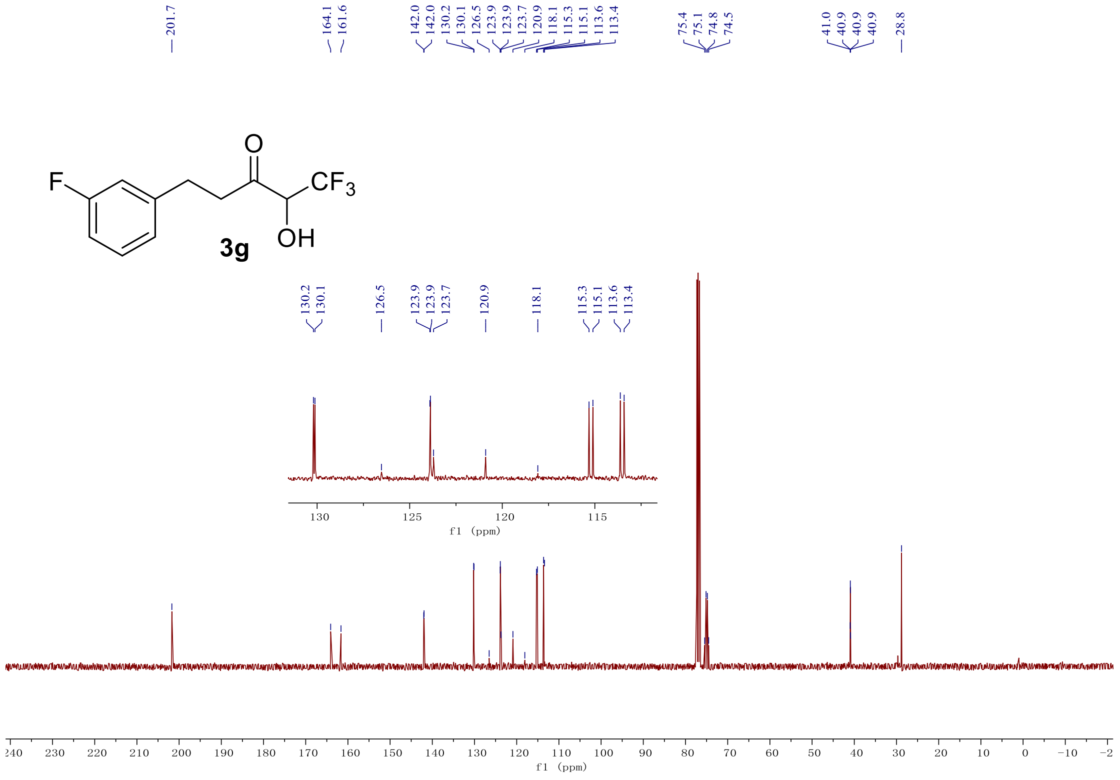

^{1}H NMR (400 MHz, $CDCl_3$)

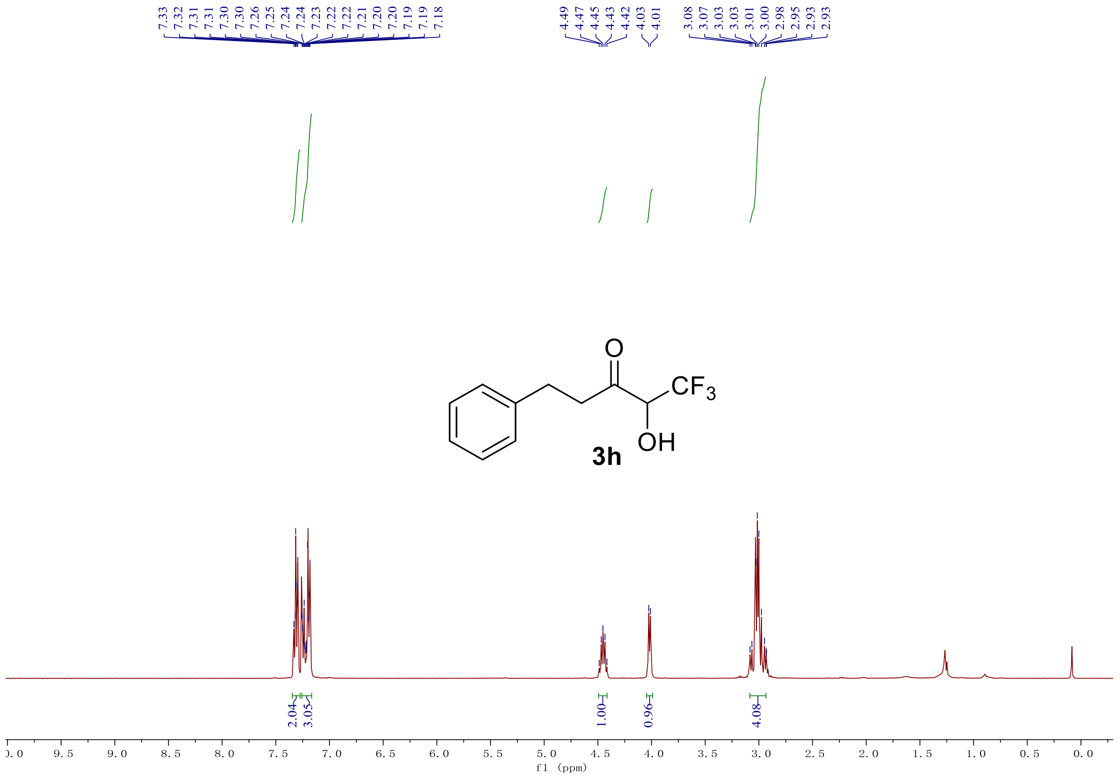

^{19}F NMR (376 MHz, $CDCl_3$)

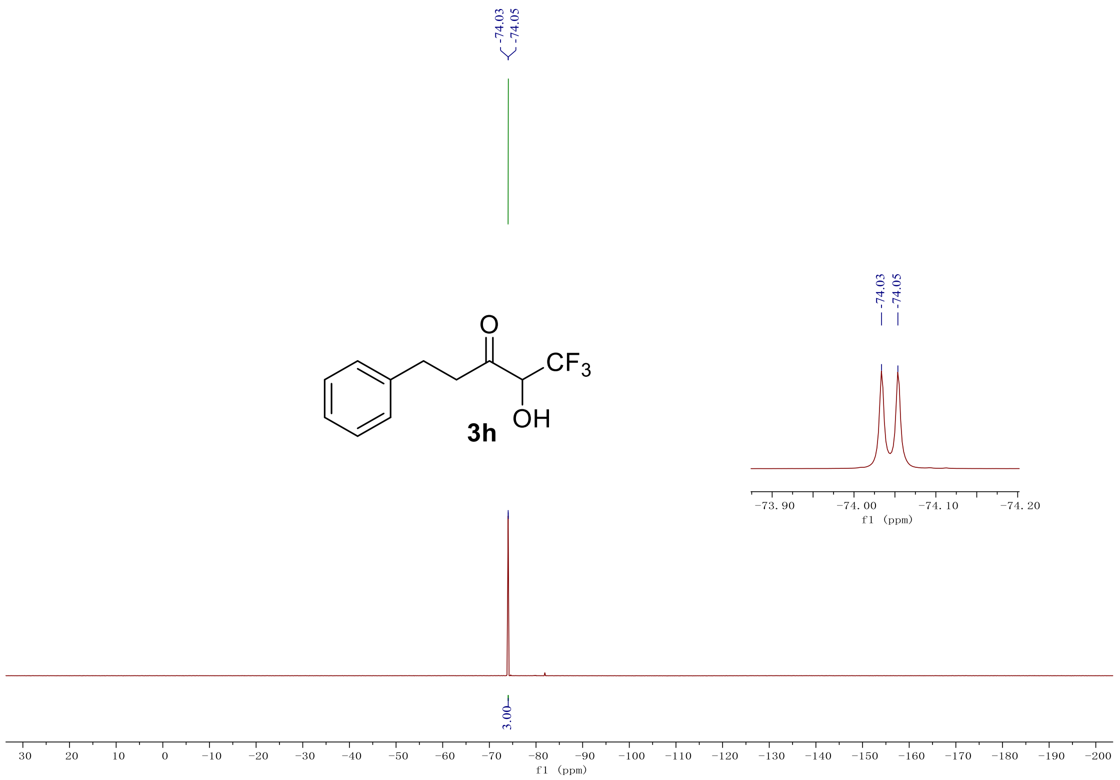

^{13}C NMR (101 MHz, $CDCl_3$)

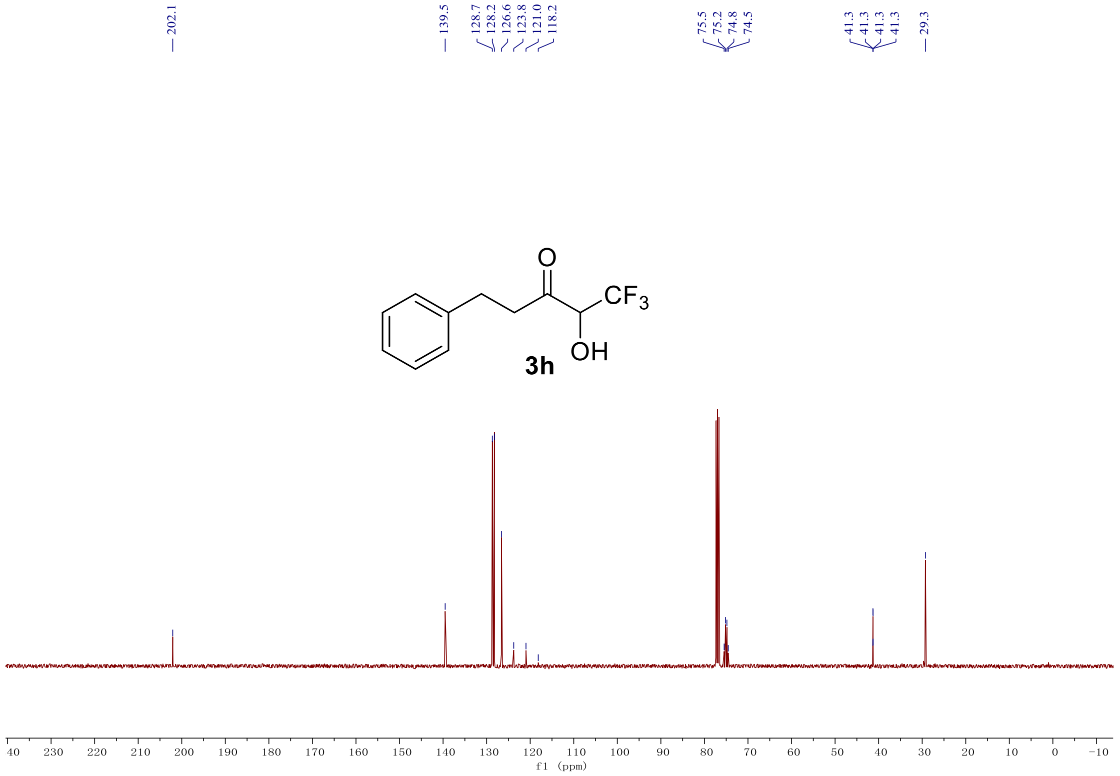

^{1}H NMR (400 MHz, $CDCl_3$)

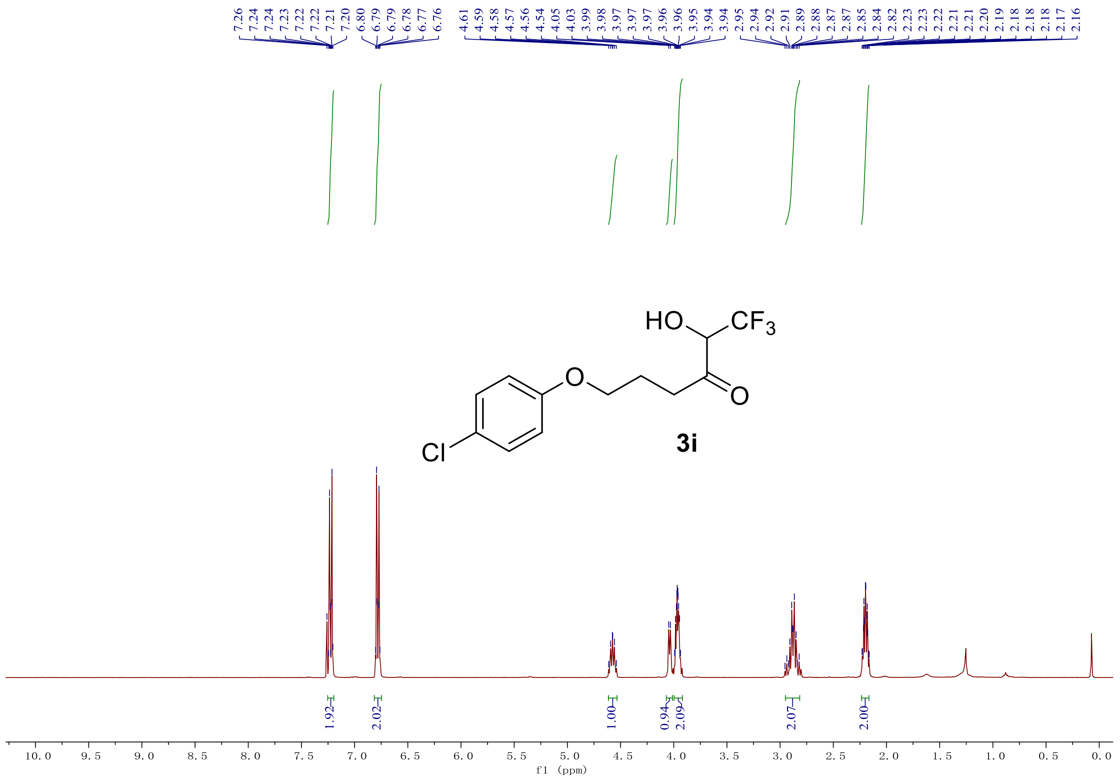

^{19}F NMR (376 MHz, $CDCl_3$)

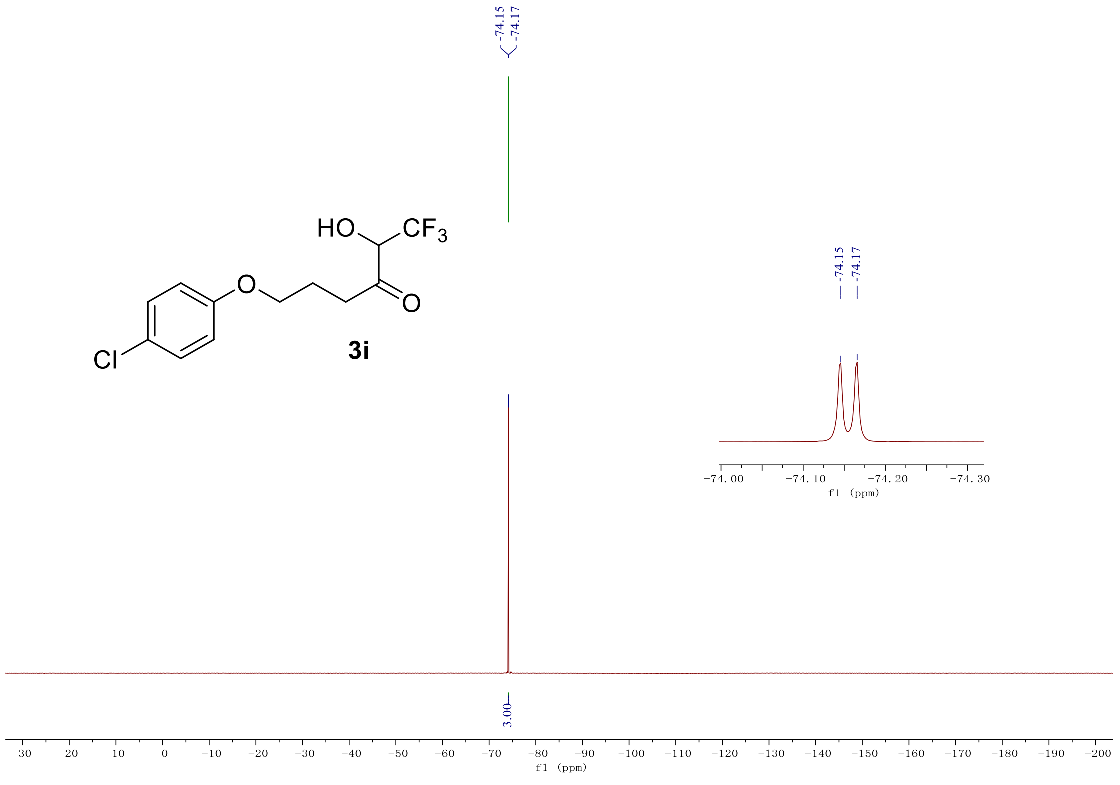

^{13}C NMR (101 MHz, $CDCl_3$)

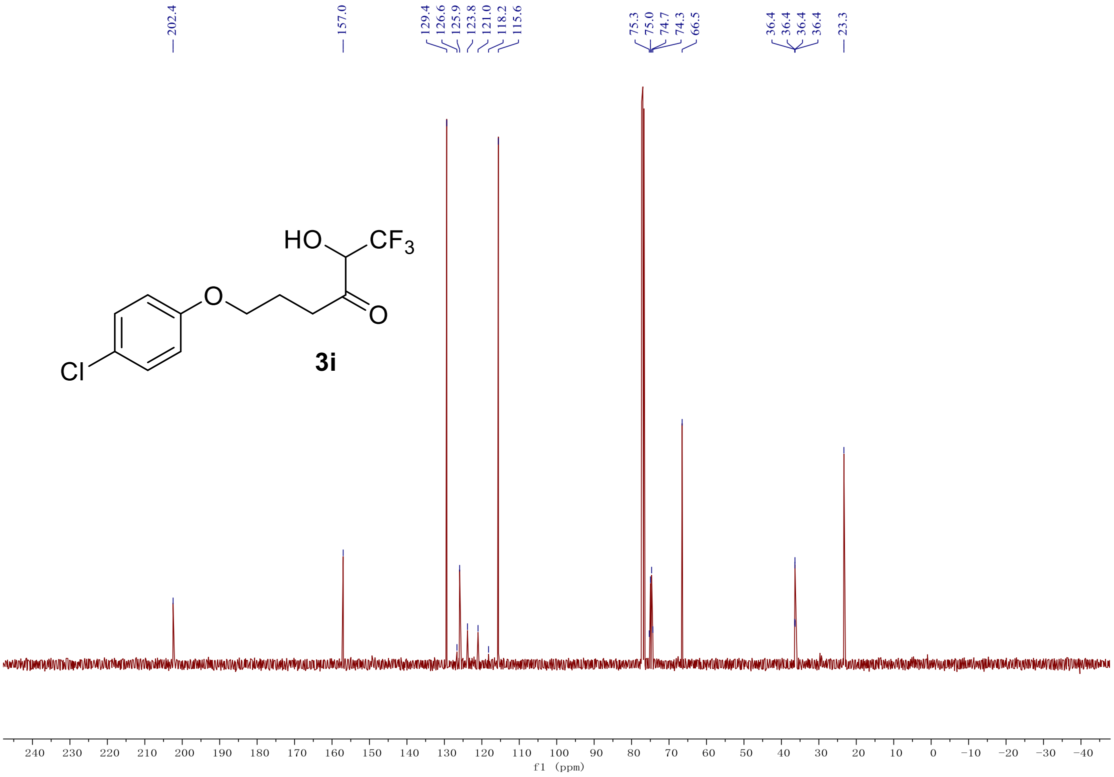

^{1}H NMR (400 MHz, $CDCl_3$)

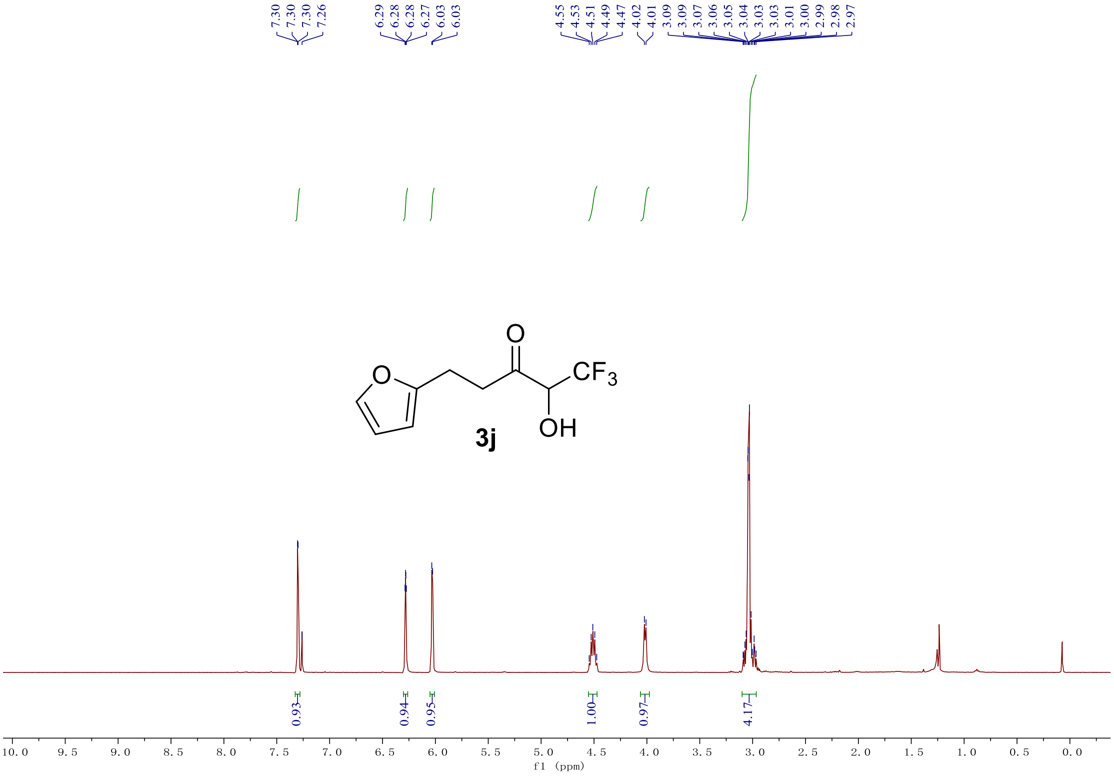

^{19}F NMR (376 MHz, $CDCl_3$)

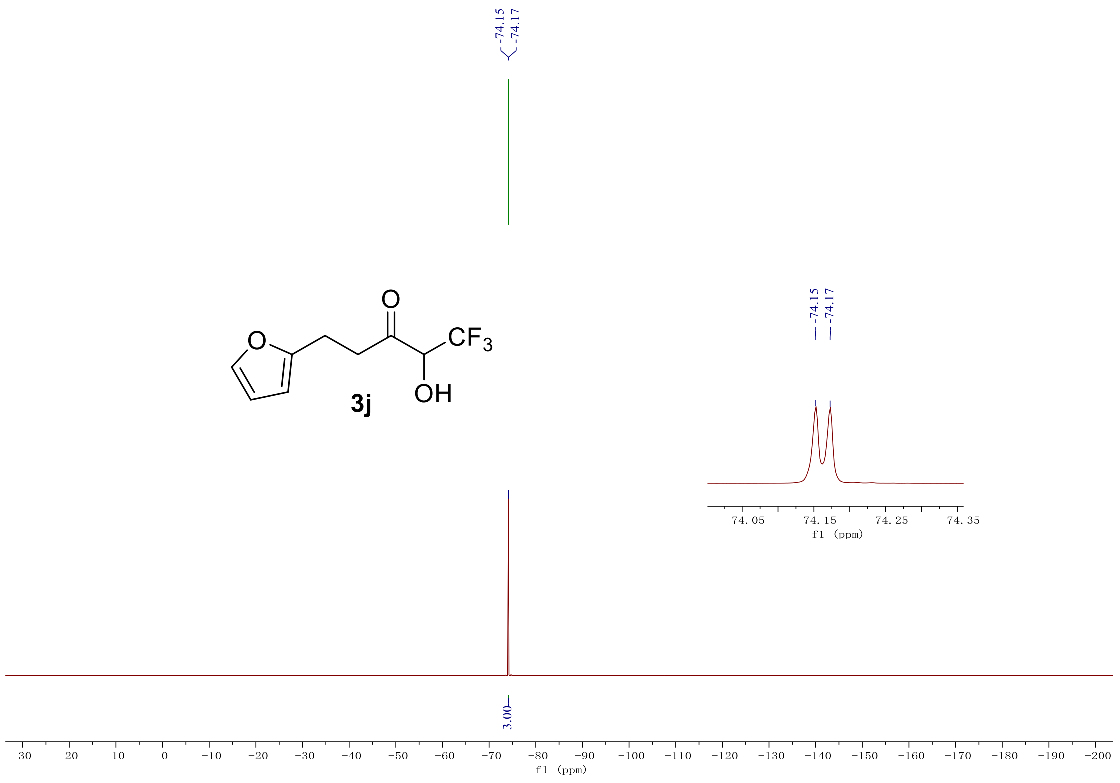

^{13}C NMR (101 MHz, $CDCl_3$)

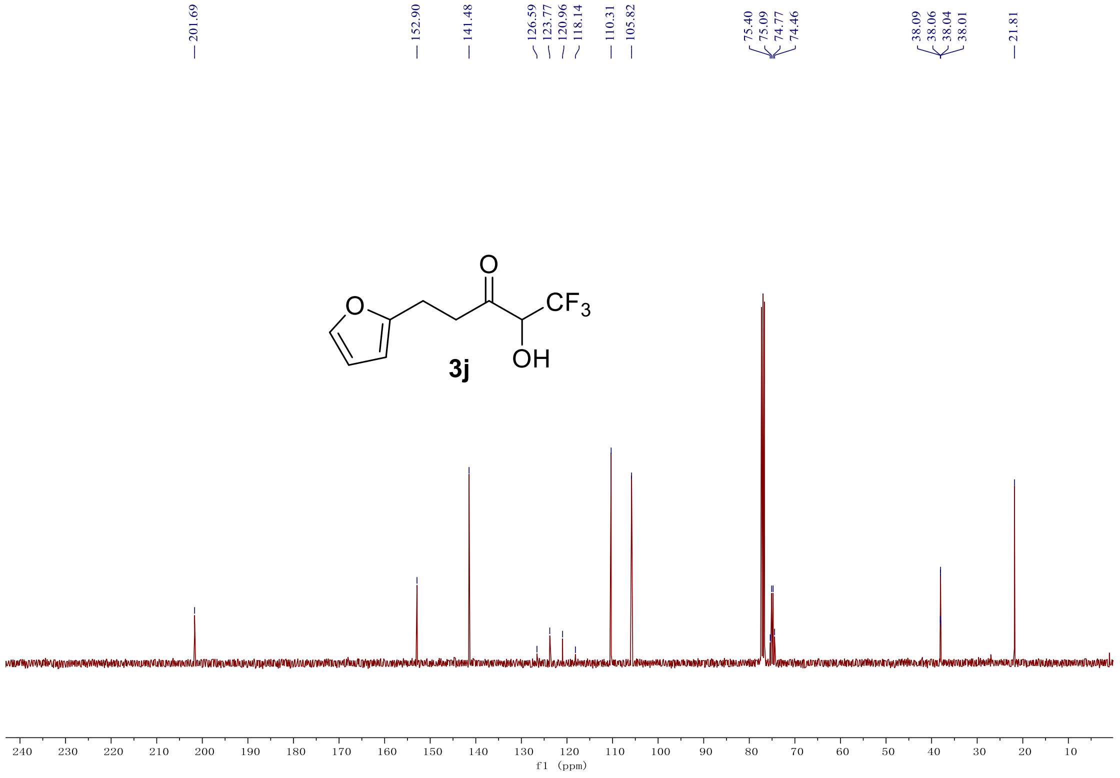

^{1}H NMR (400 MHz, $CDCl_3$)

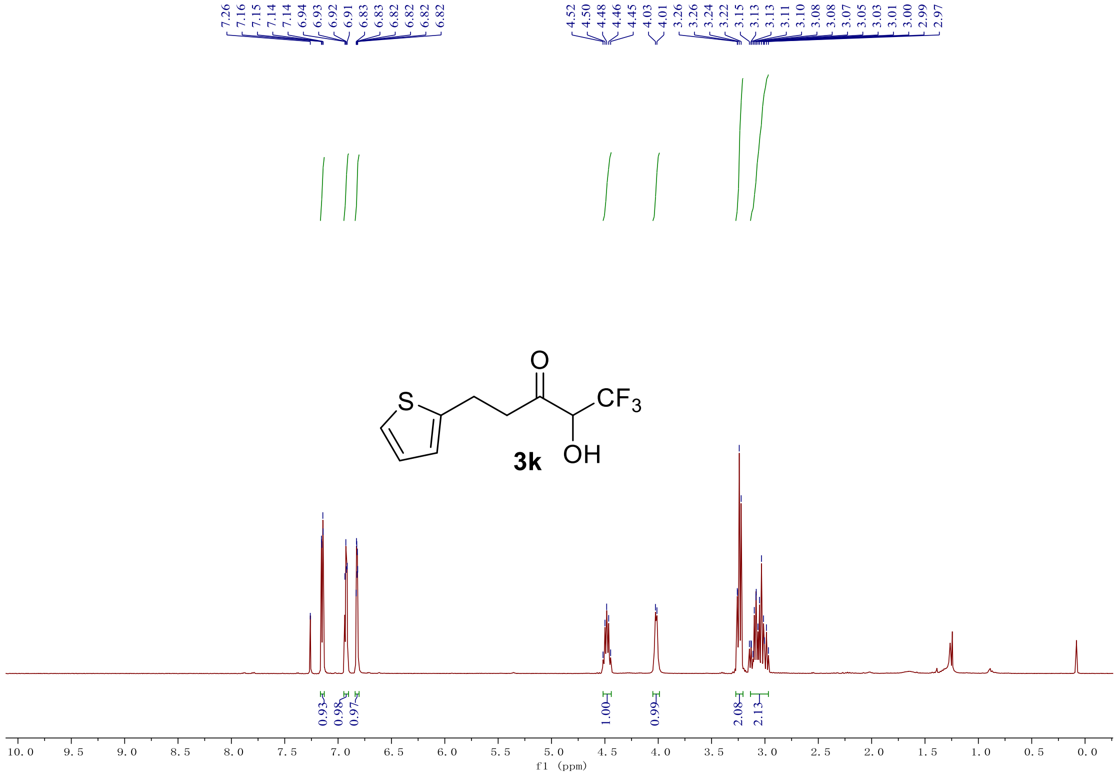

^{19}F NMR (376 MHz, $CDCl_3$)

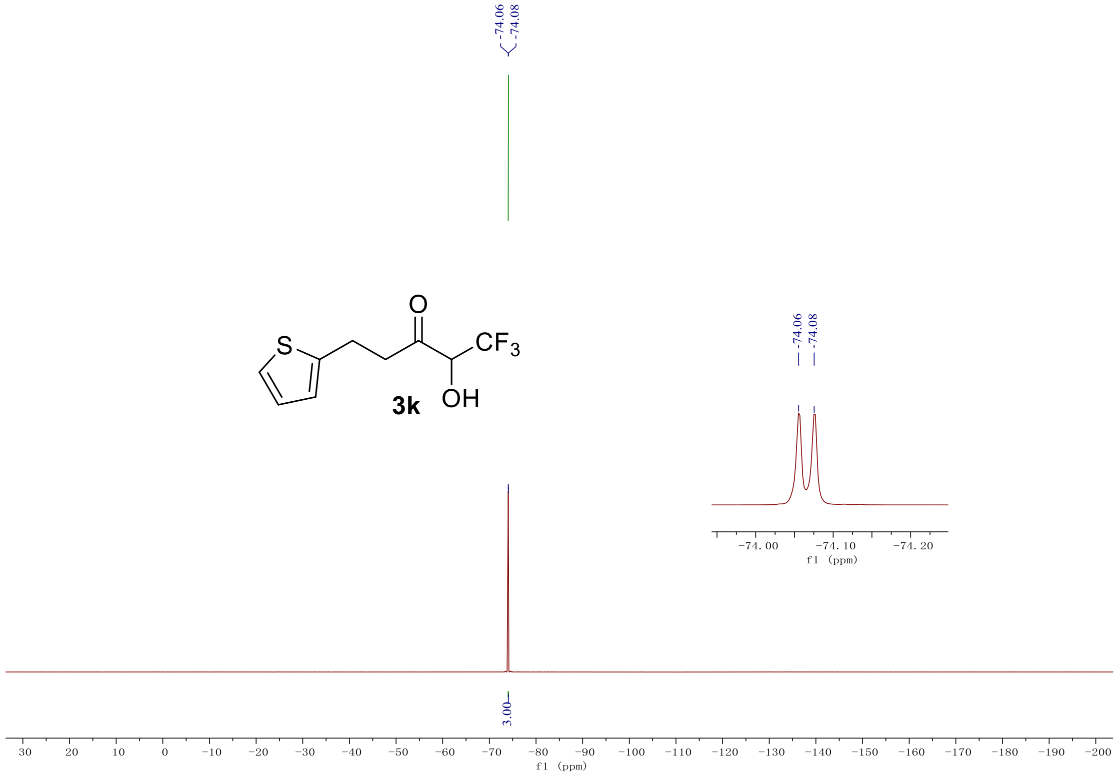

^{13}C NMR (101 MHz, $CDCl_3$)

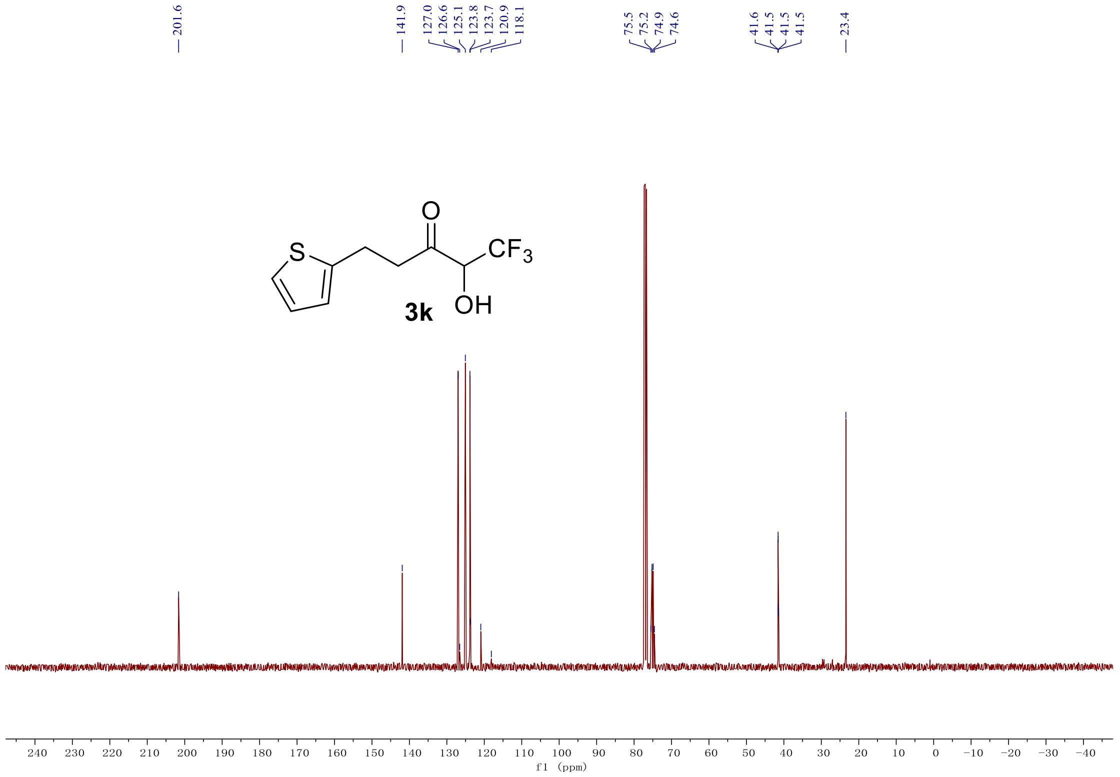

^{1}H NMR (400 MHz, $CDCl_3$)

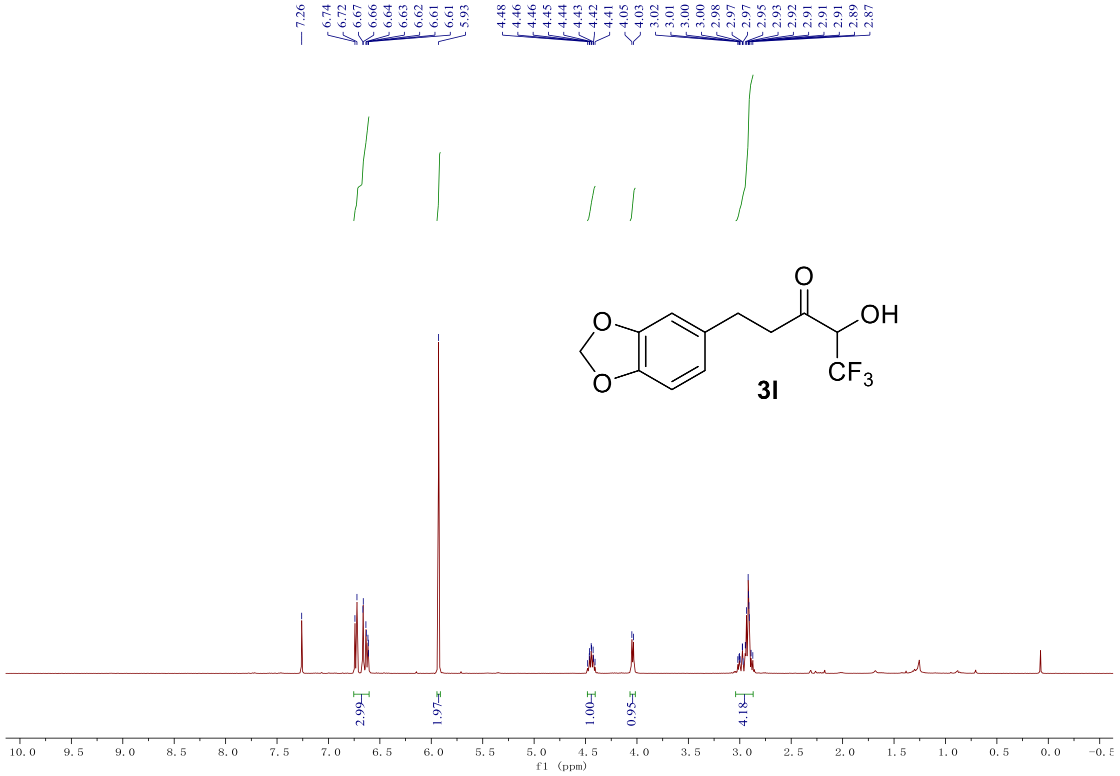

^{19}F NMR (376 MHz, $CDCl_3$)

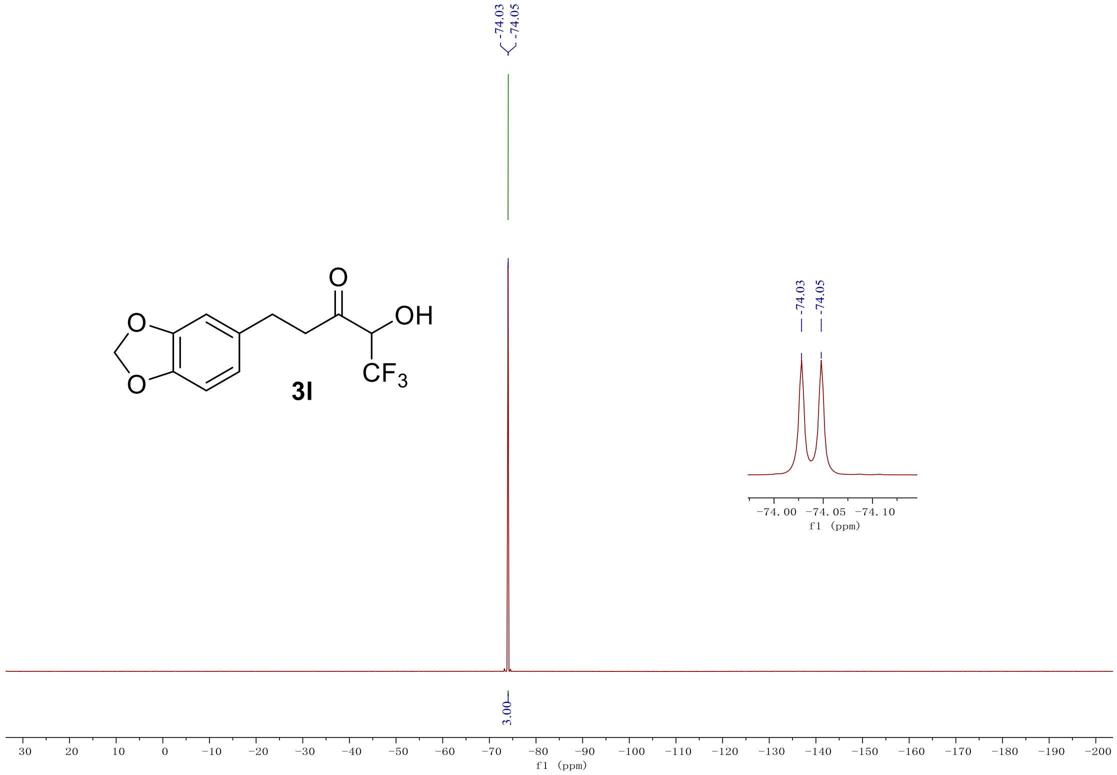

^{13}C NMR (101 MHz, $CDCl_3$)

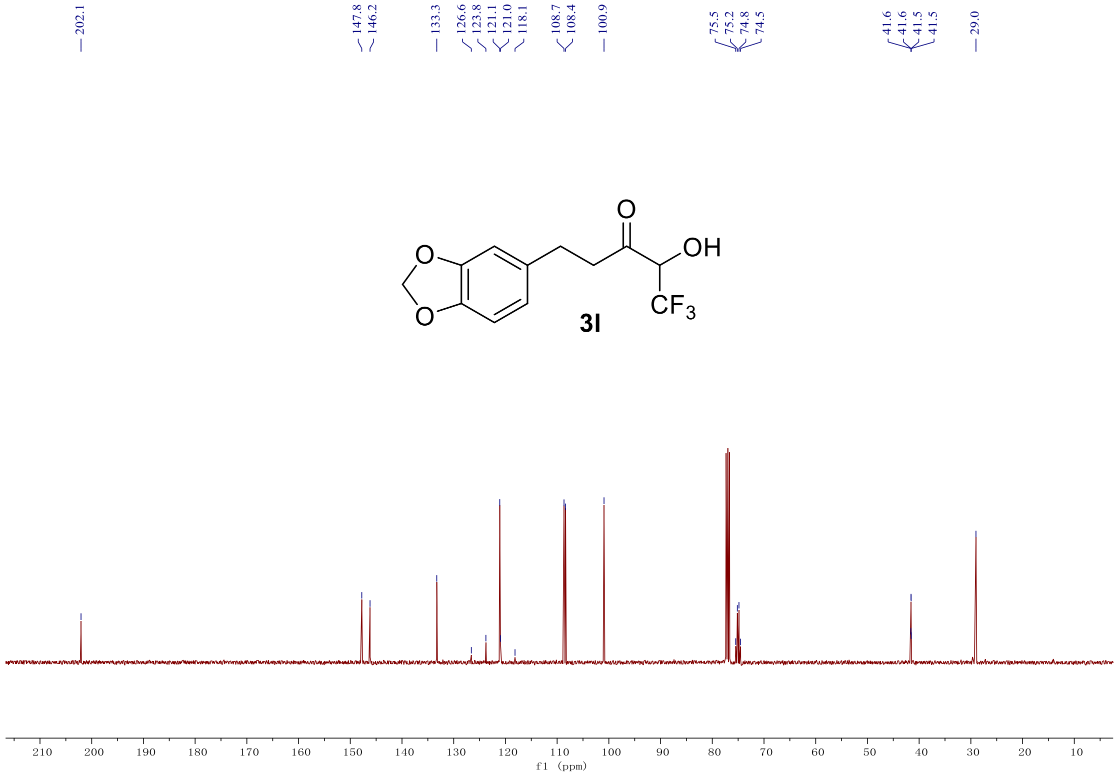

^{1}H NMR (400 MHz, $(CD_3)_2CO$)

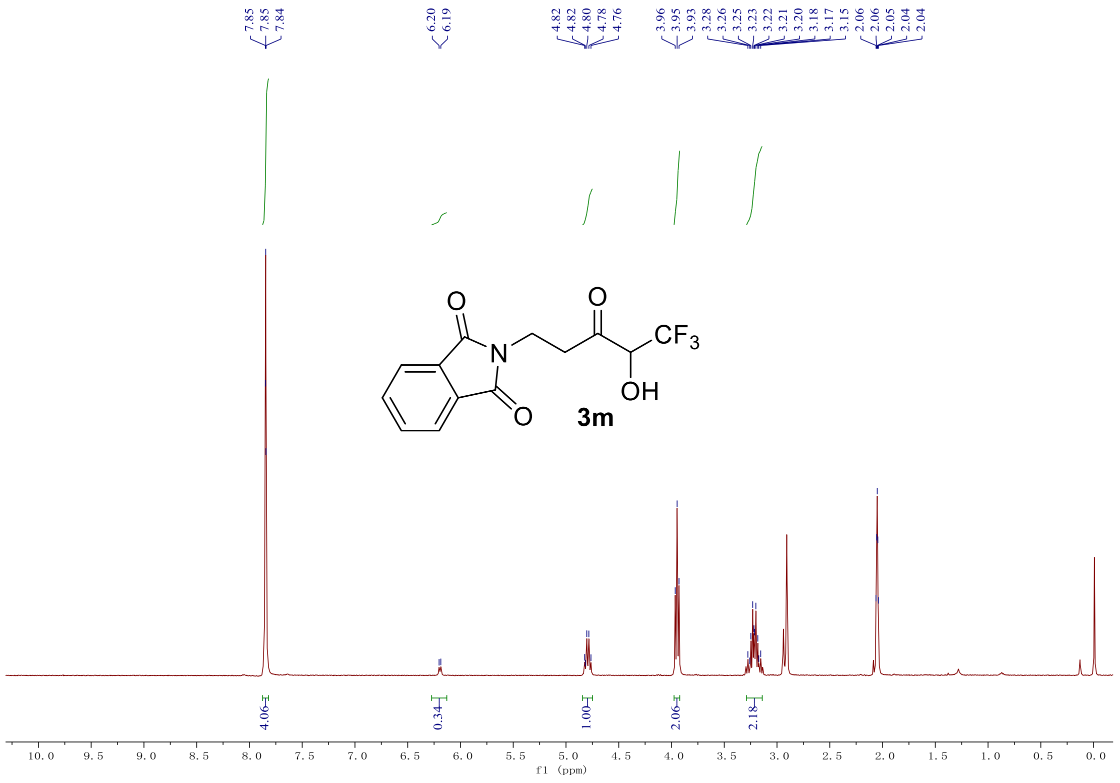

^{19}F NMR (376 MHz, $(CD_3)_2CO$)

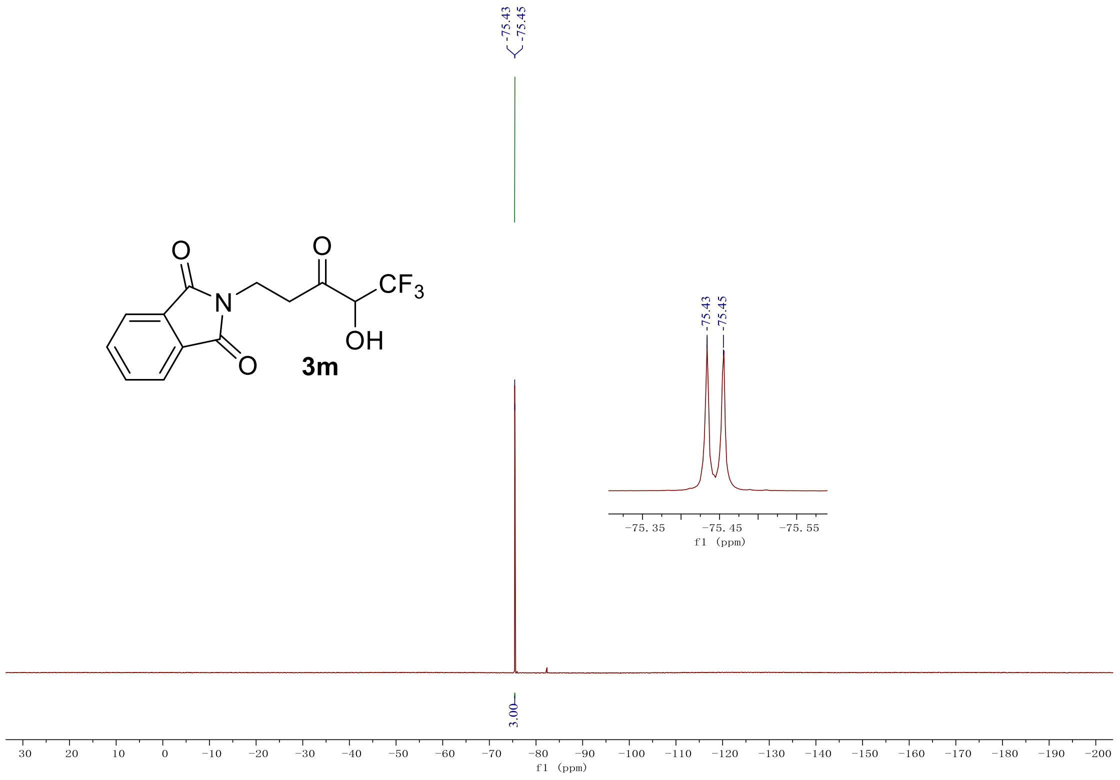

^{13}C NMR (101 MHz, $(CD_3)_2CO$)

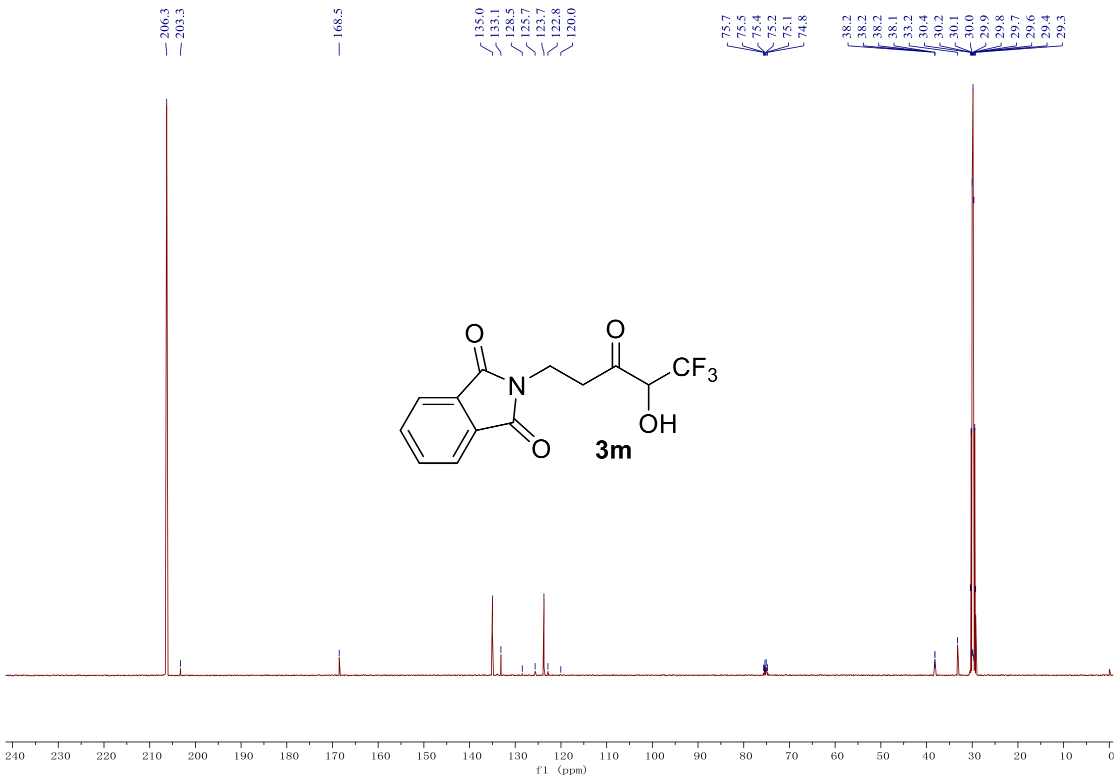

^{1}H NMR (400 MHz, $CDCl_3$)

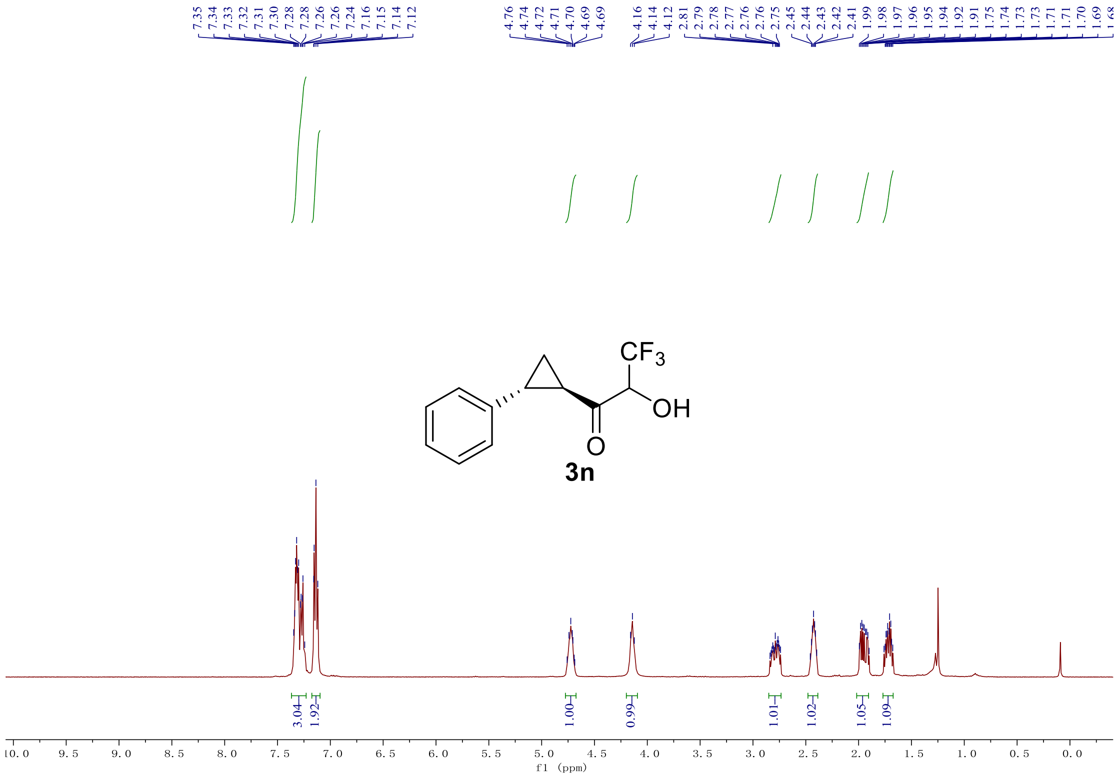

^{19}F NMR (376 MHz, CDCl$_3$)

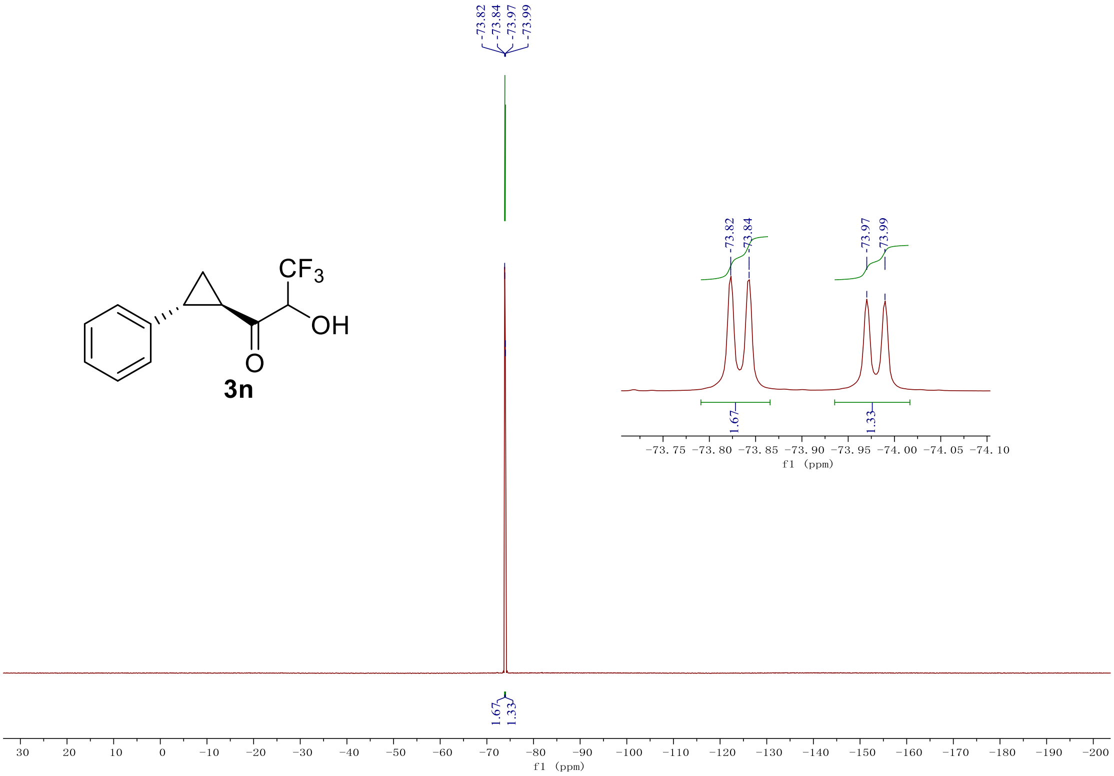

^{13}C NMR (101 MHz, CDCl$_3$)

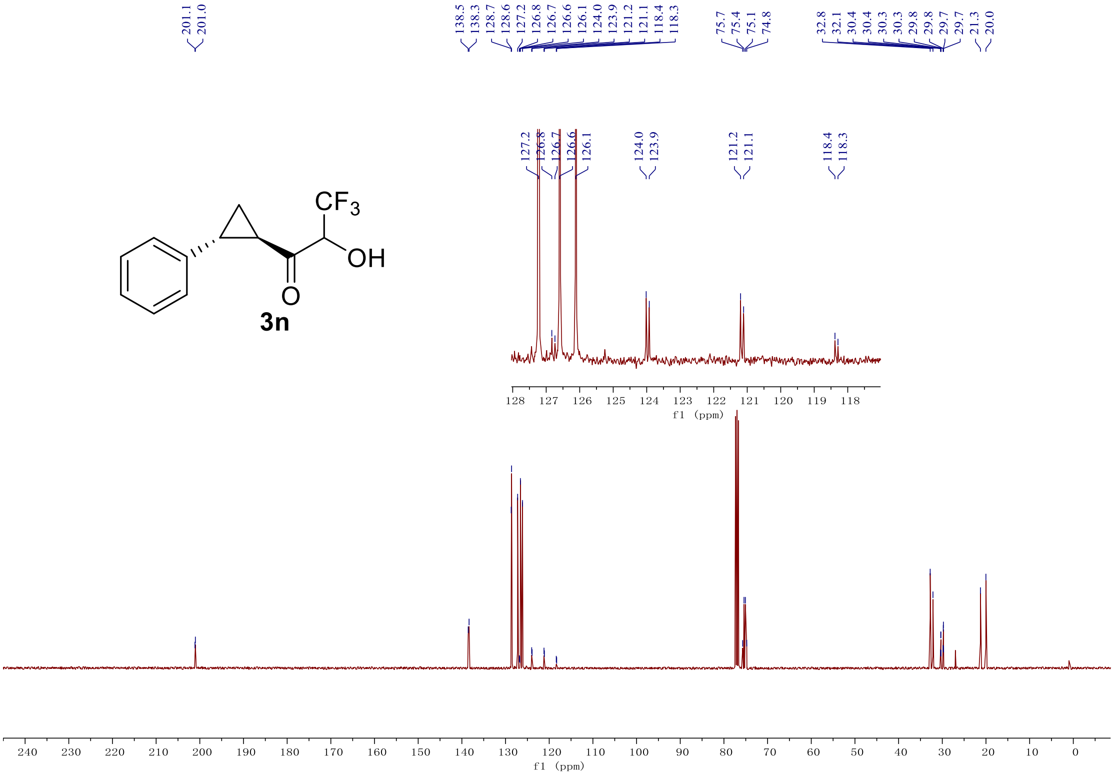

^{1}H NMR (400 MHz, $CDCl_3$)

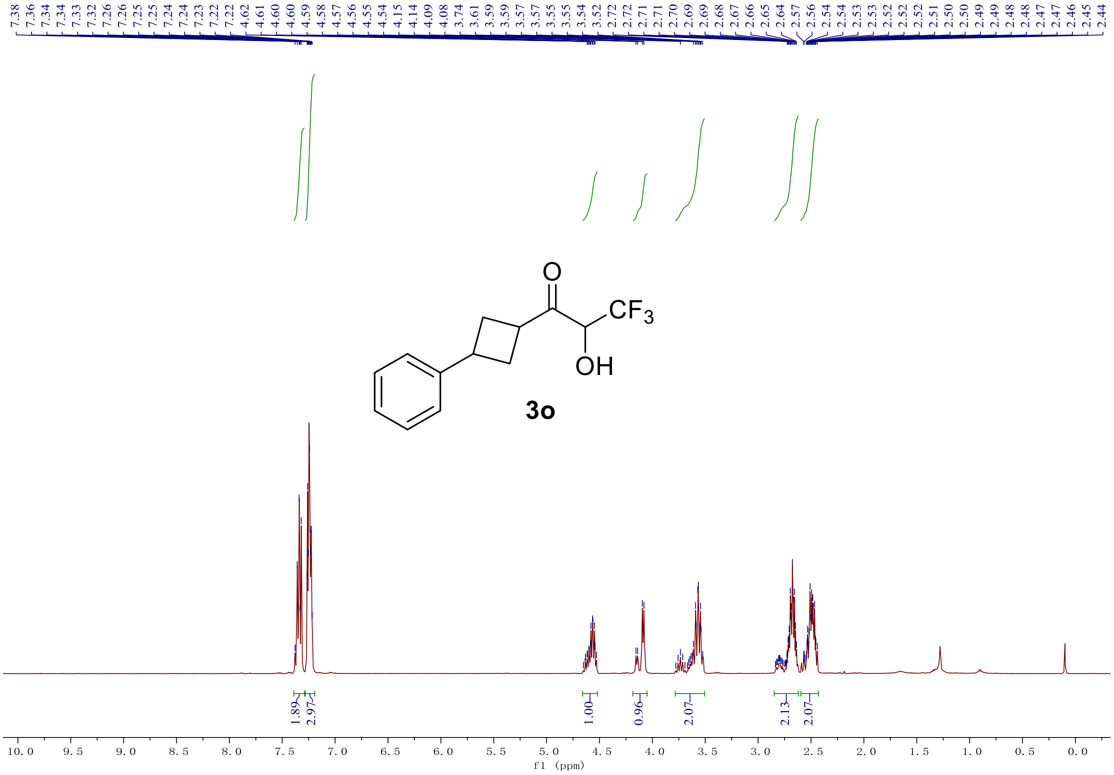

^{19}F NMR (376 MHz, $CDCl_3$)

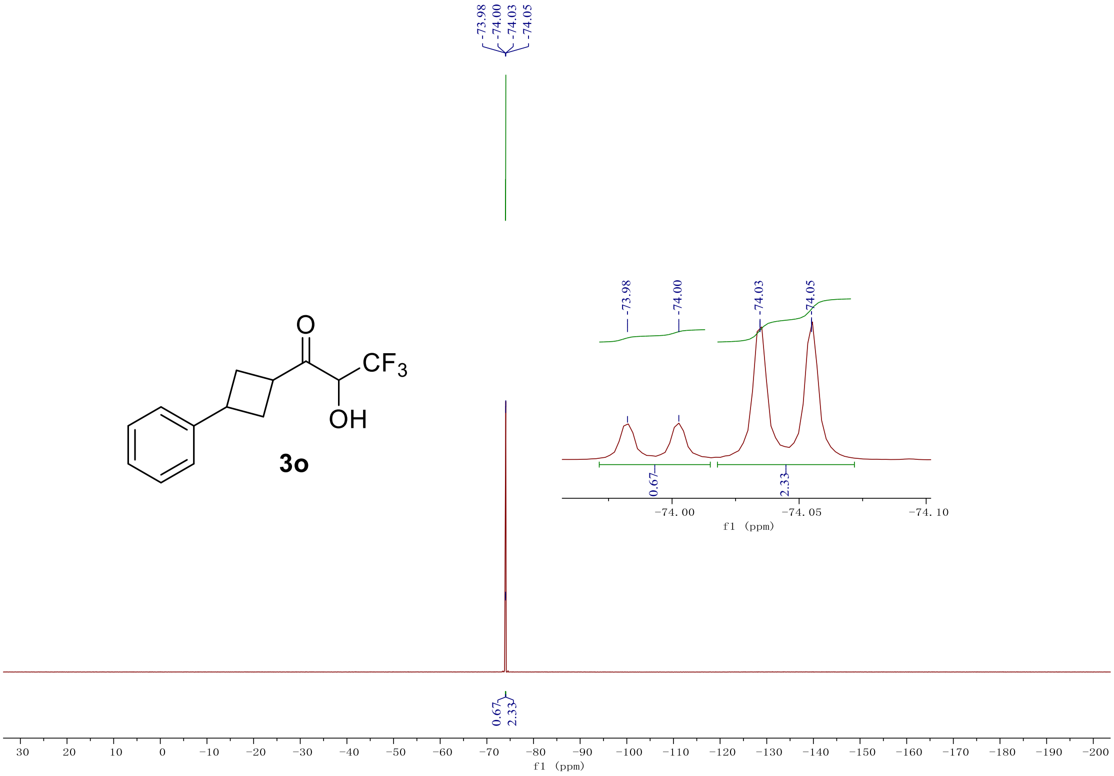

^{13}C NMR (101 MHz, $CDCl_3$)

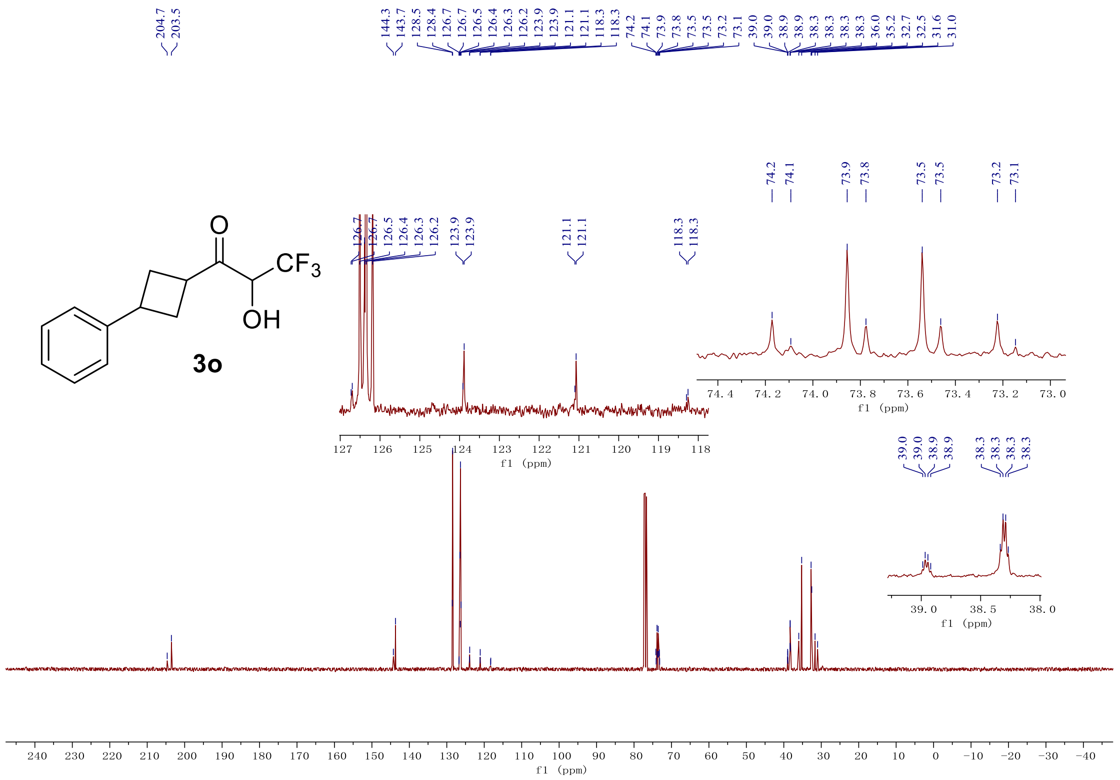

^{1}H NMR (400 MHz, $CDCl_3$)

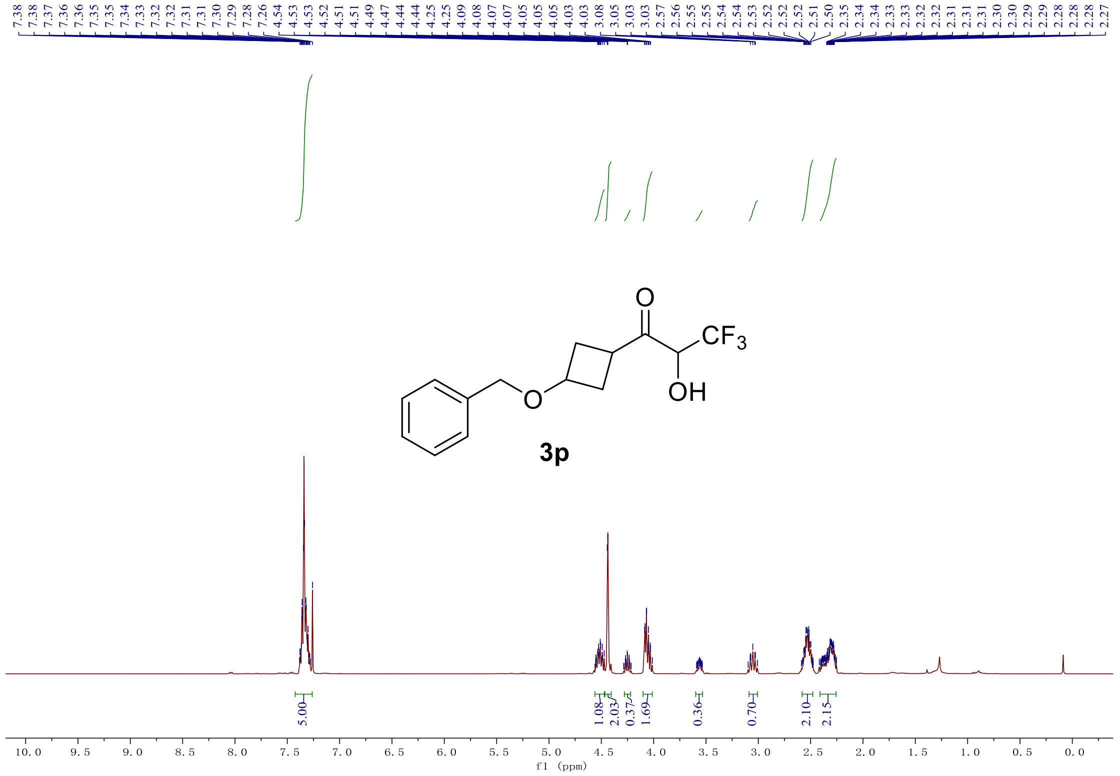

^{19}F NMR (376 MHz, $CDCl_3$)

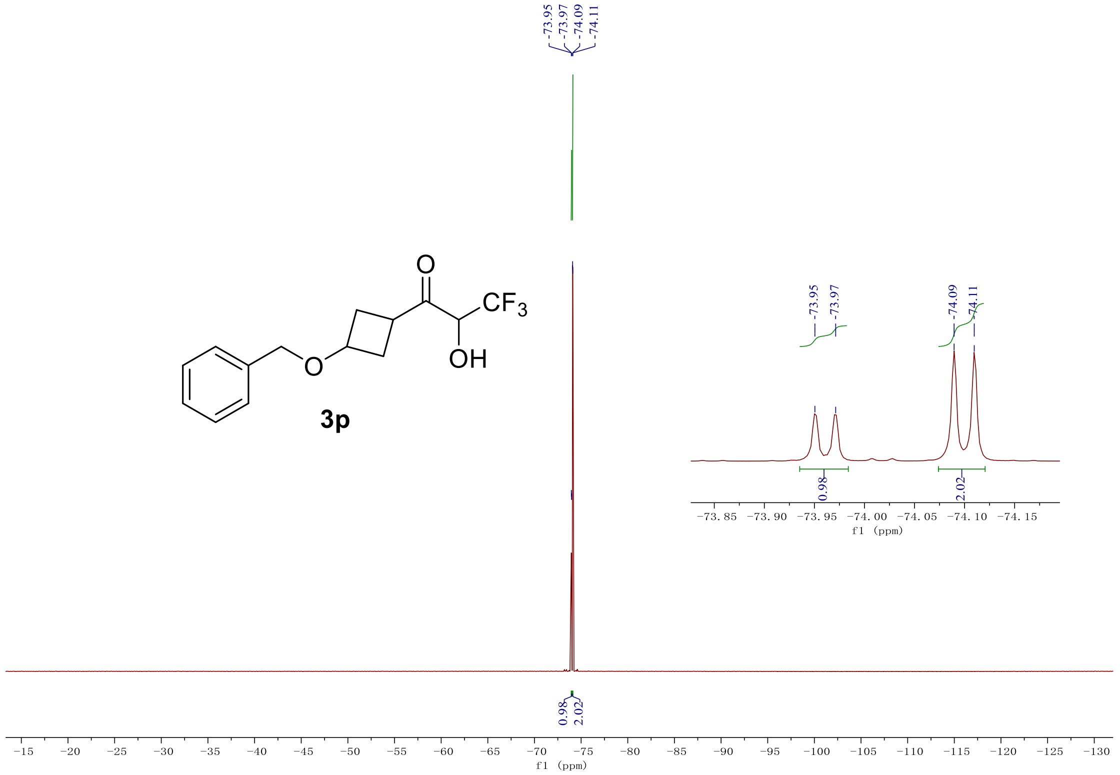

^{13}C NMR (101 MHz, $CDCl_3$)

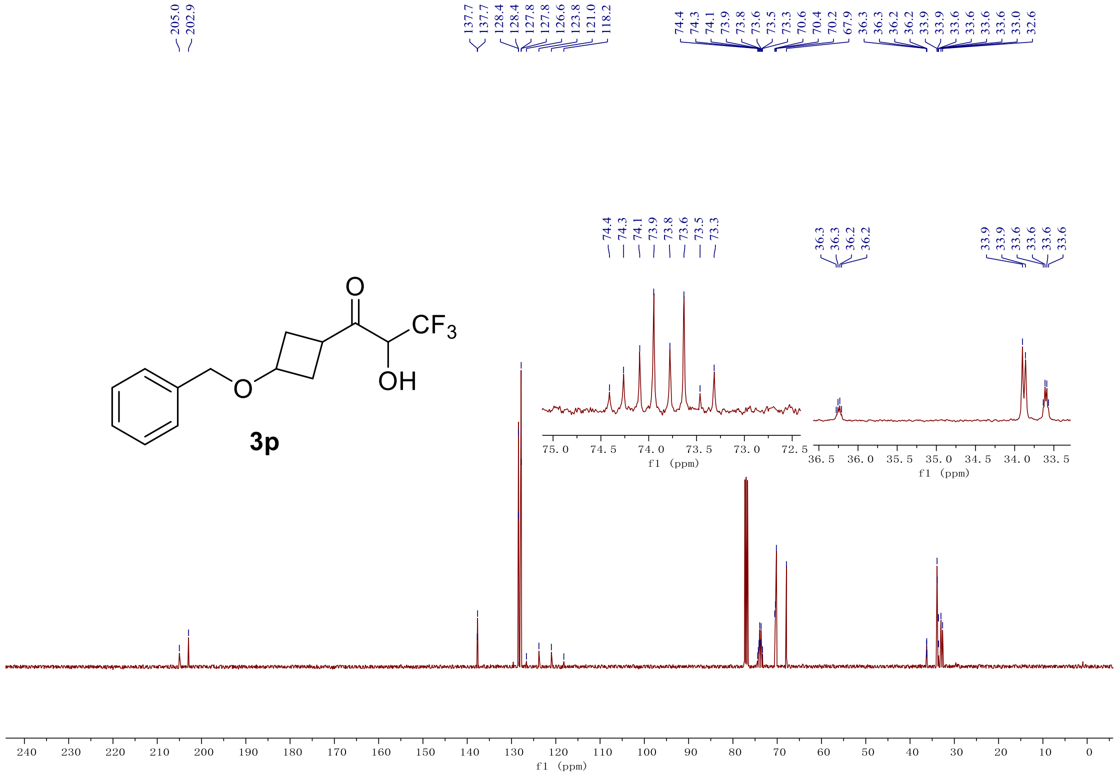

^1H NMR (400 MHz, $CDCl_3$)

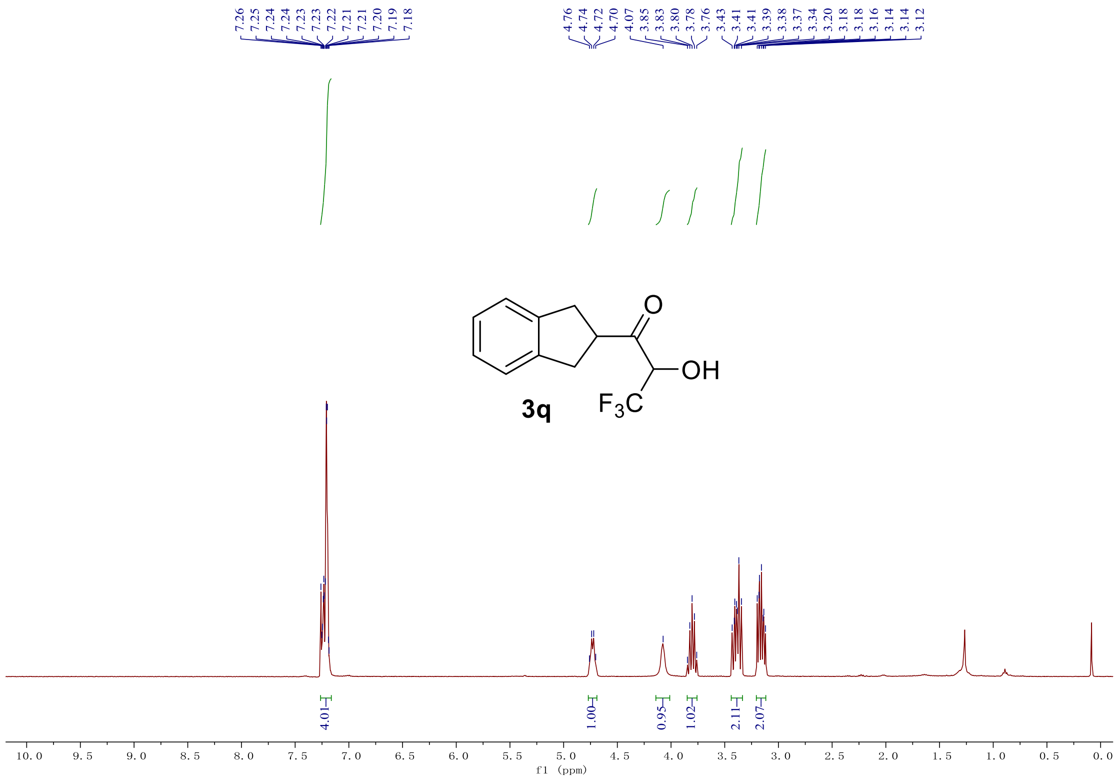

^{19}F NMR (376 MHz, $CDCl_3$)

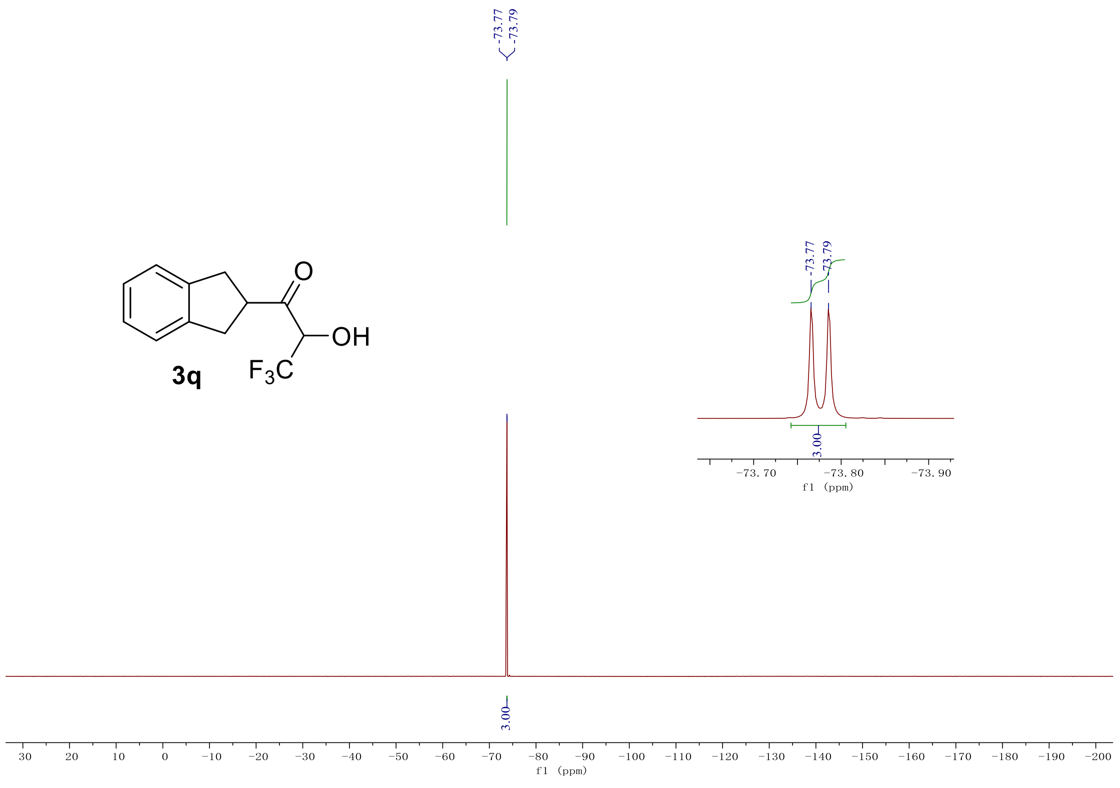

^{13}C NMR (101 MHz, $CDCl_3$)

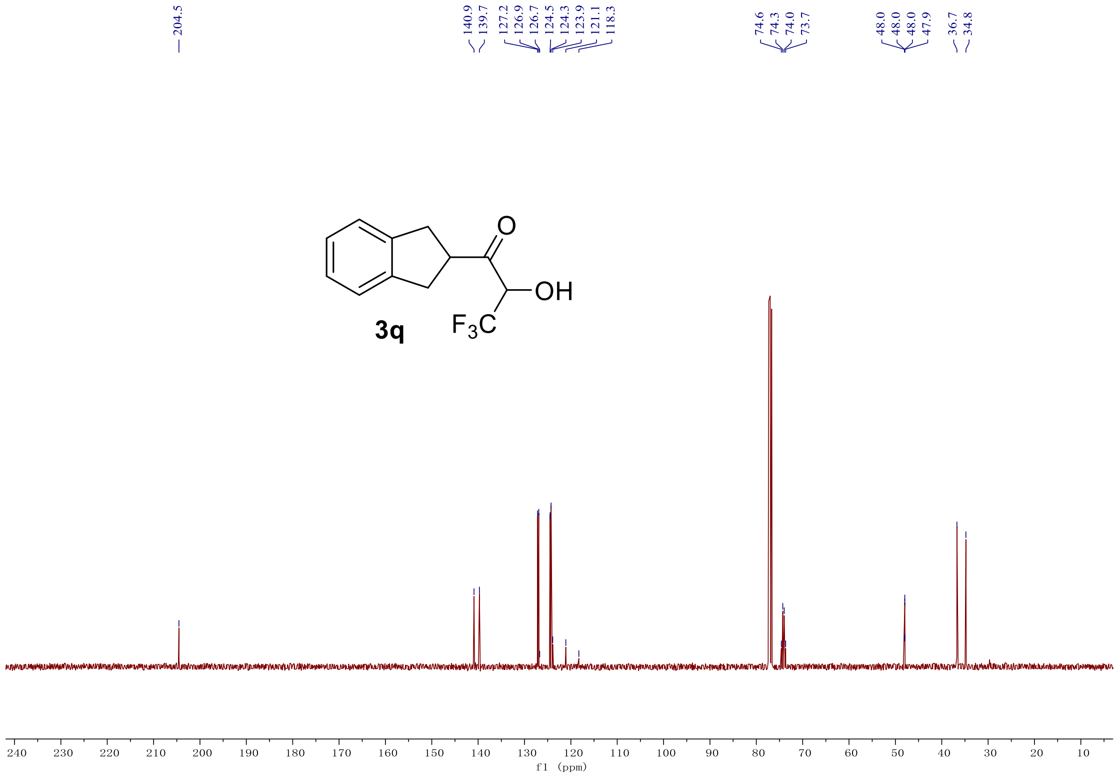

^{1}H NMR (400 MHz, $CDCl_3$)

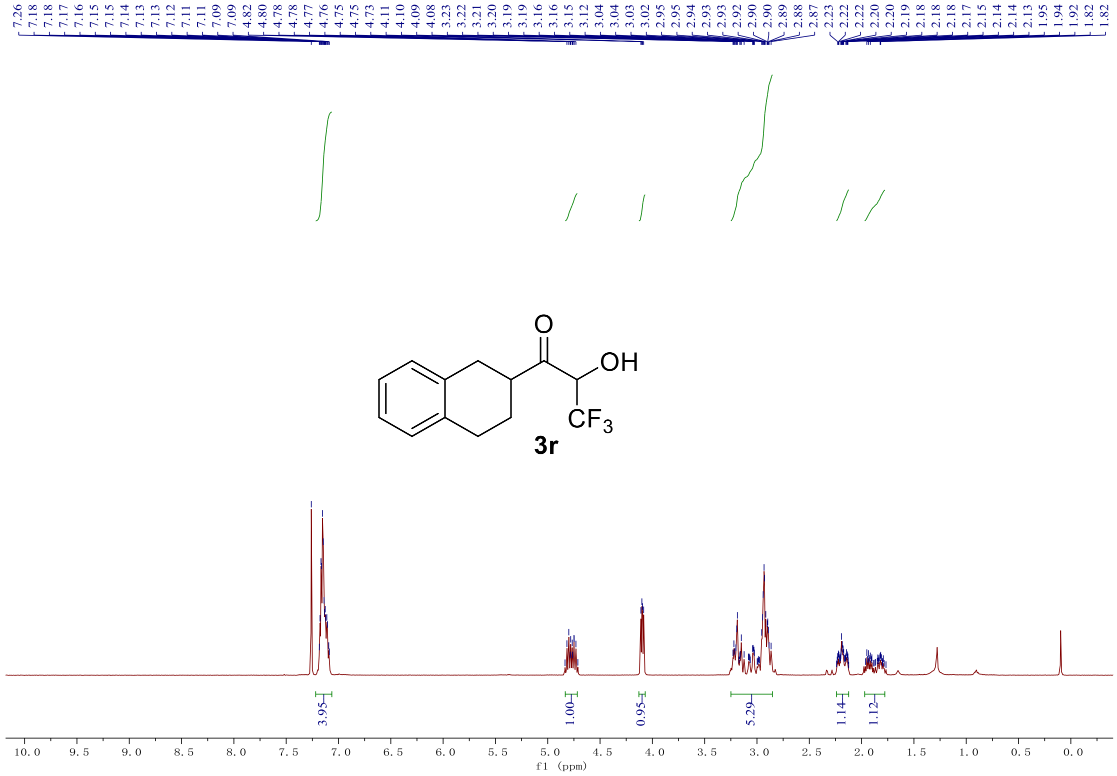

^{19}F NMR (376 MHz, $CDCl_3$)

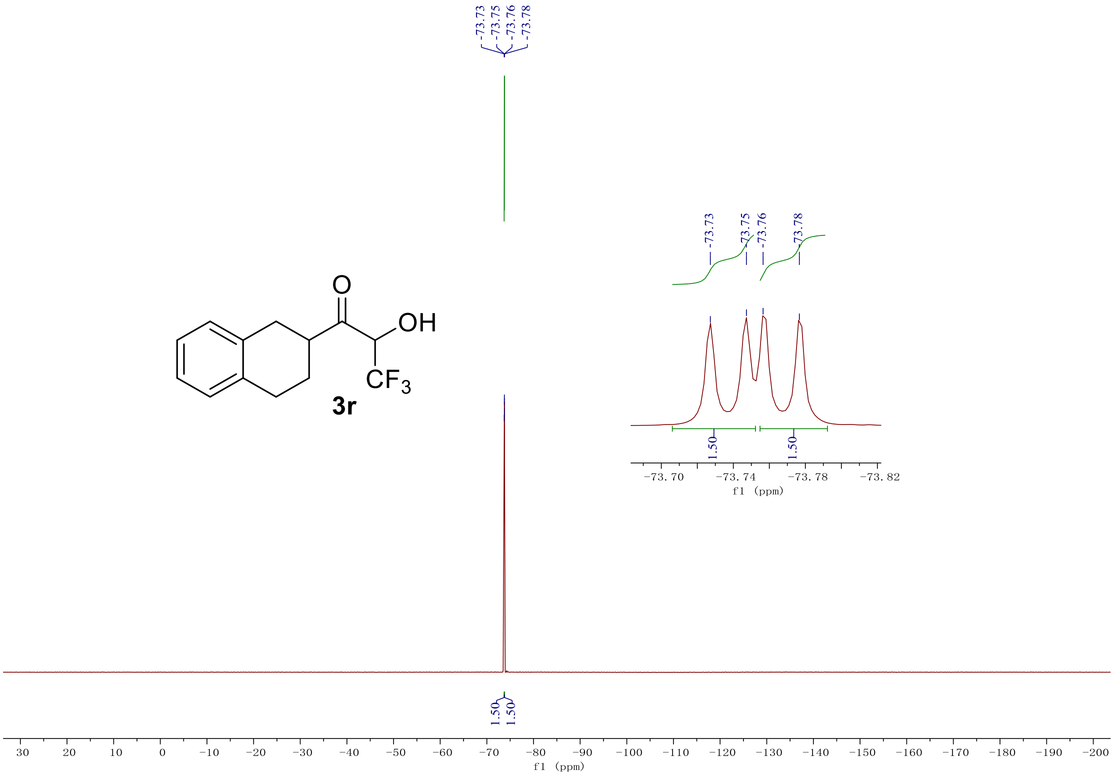

^{13}C NMR (101 MHz, $CDCl_3$)

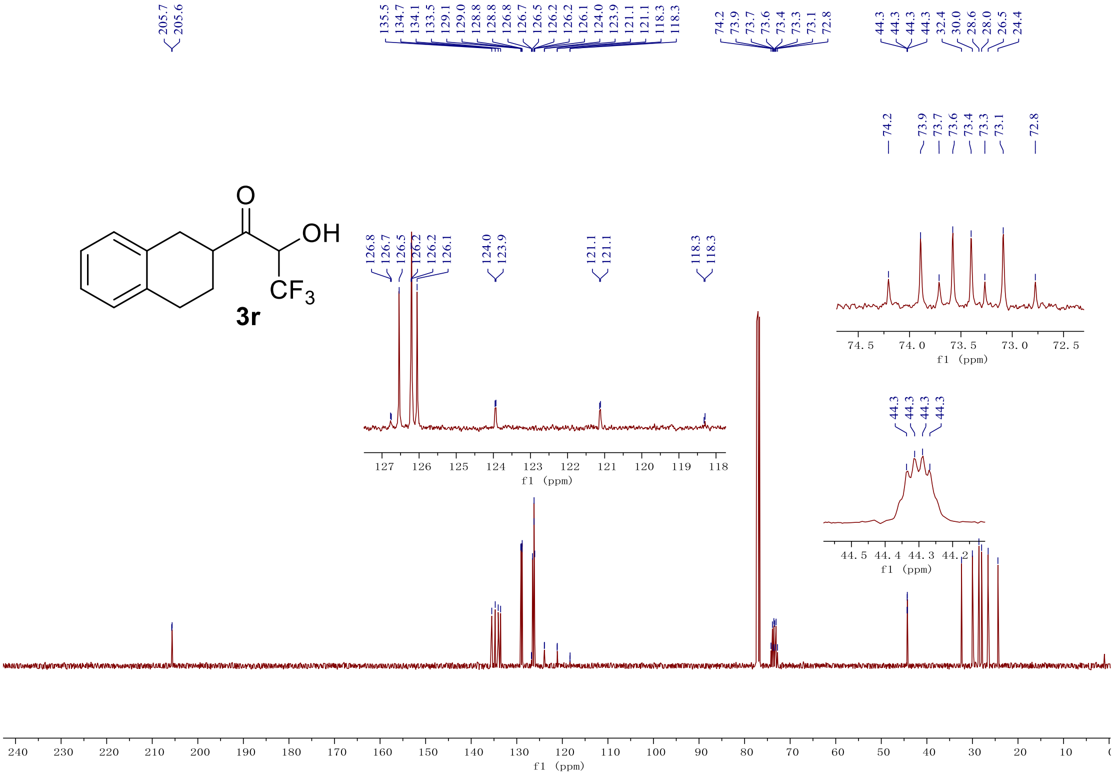

^{1}H NMR (400 MHz, CDCl₃)

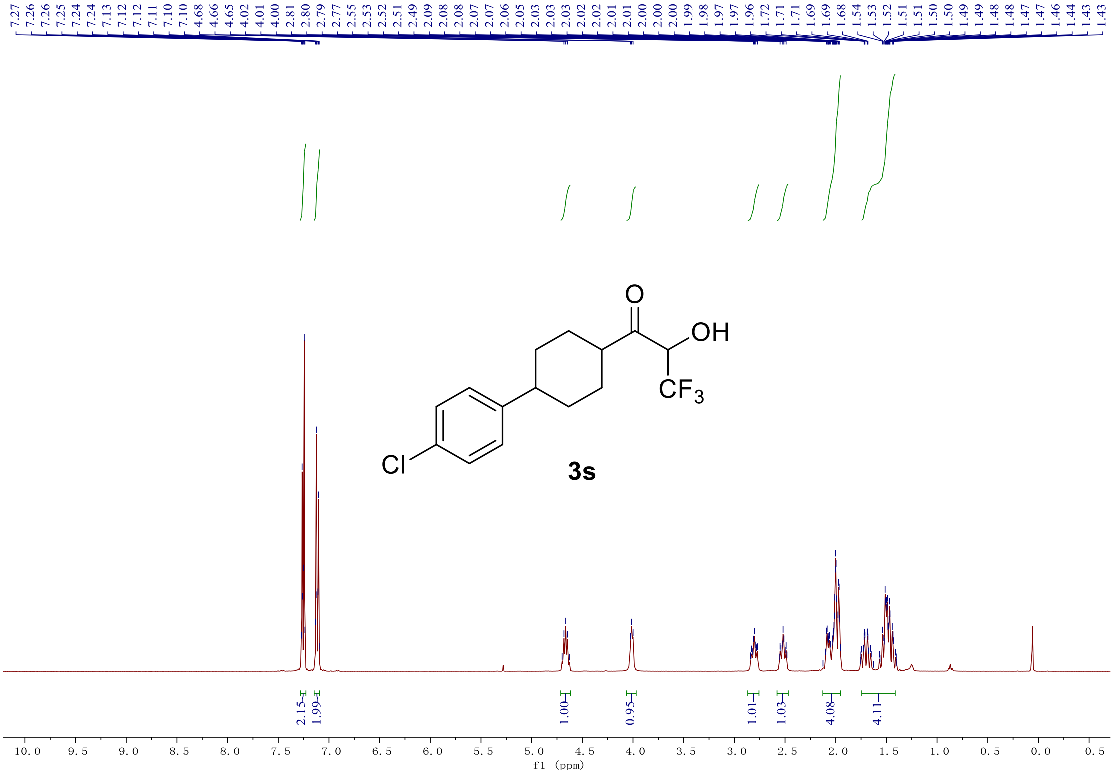

^{19}F NMR (376 MHz, CDCl₃)

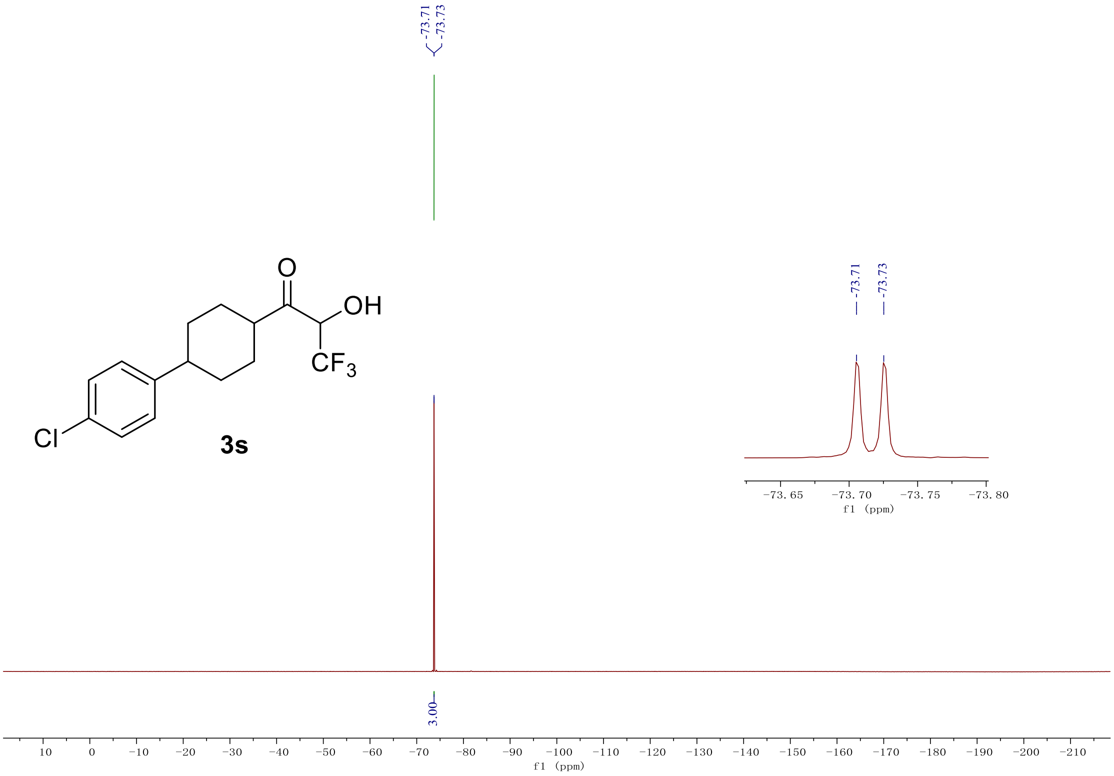

^{13}C *NMR (101 MHz, CDCl$_3$)*

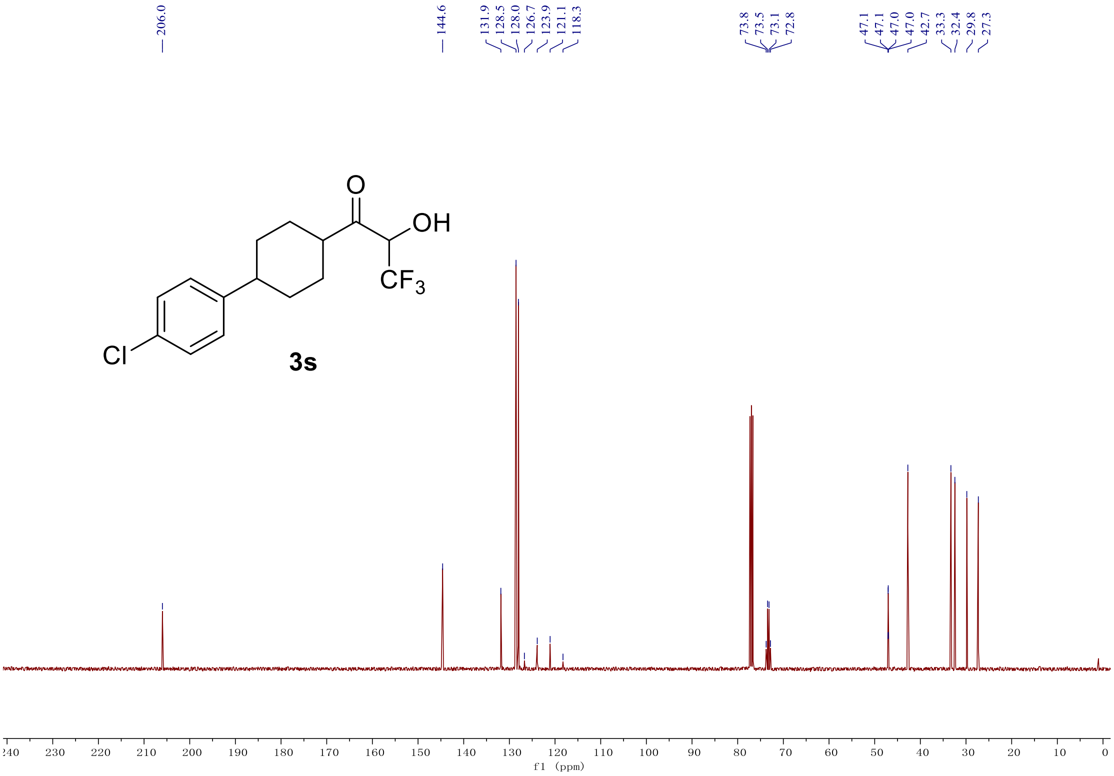

^{1}H *NMR (400 MHz, CDCl$_3$)*

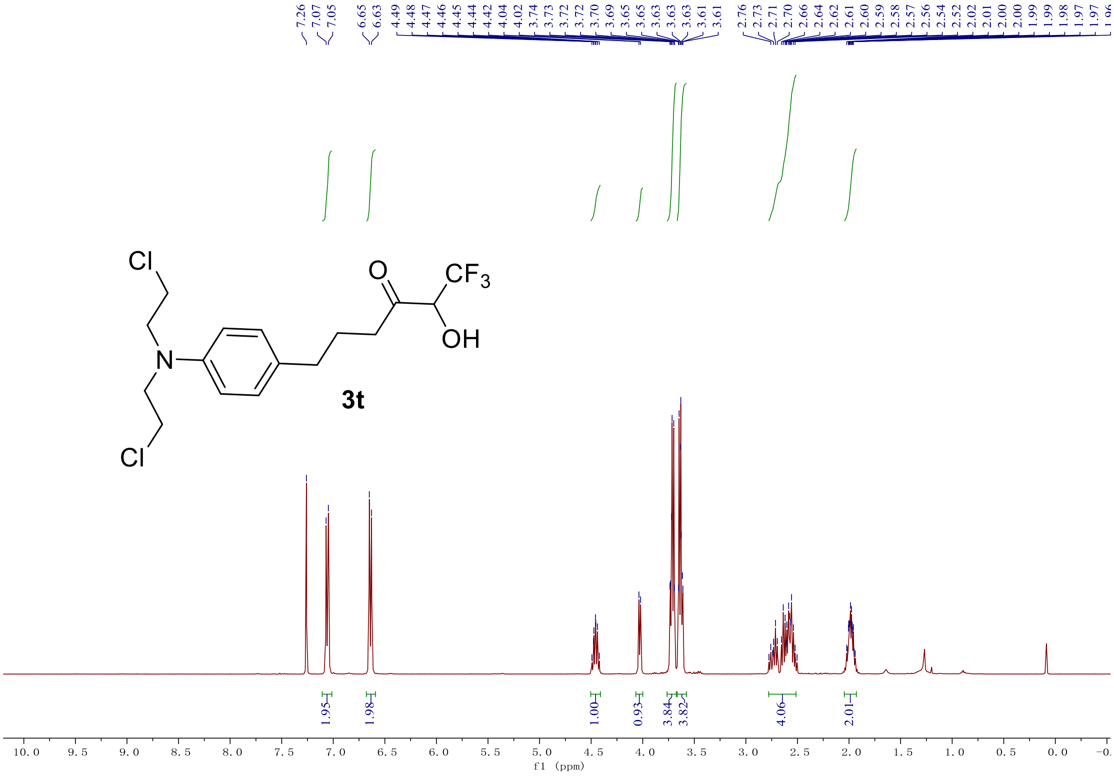

^{19}F NMR (376 MHz, $CDCl_3$)

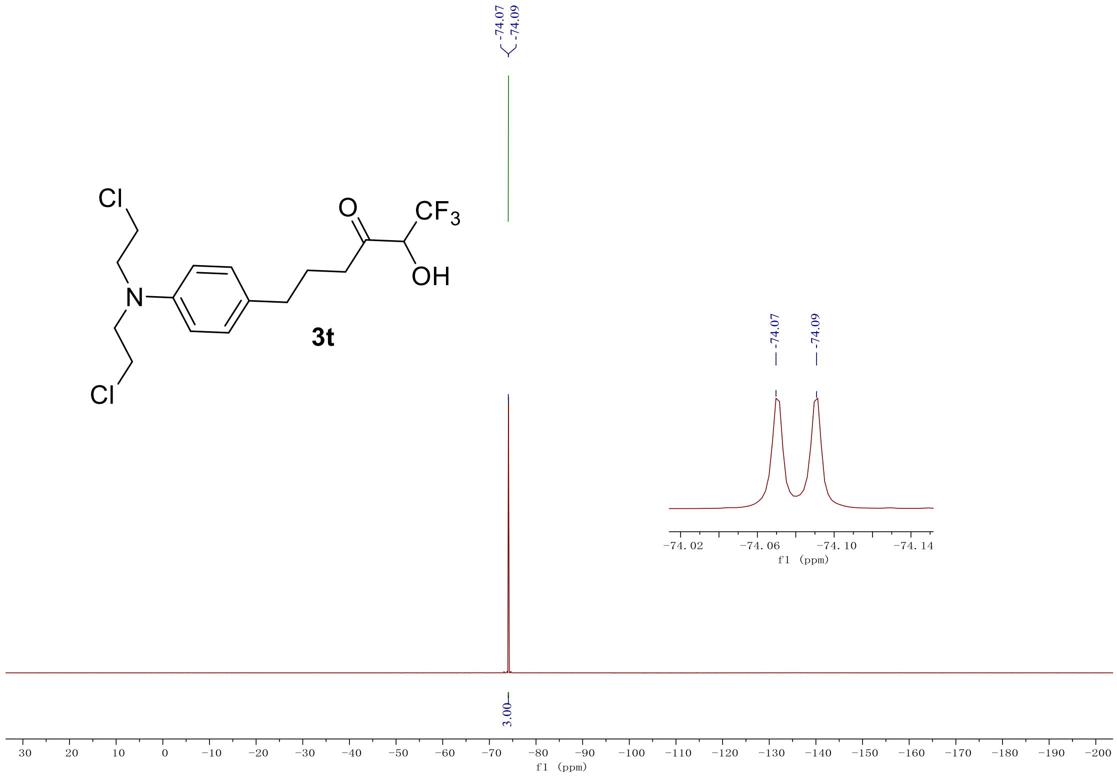

^{13}C NMR (101 MHz, $CDCl_3$)

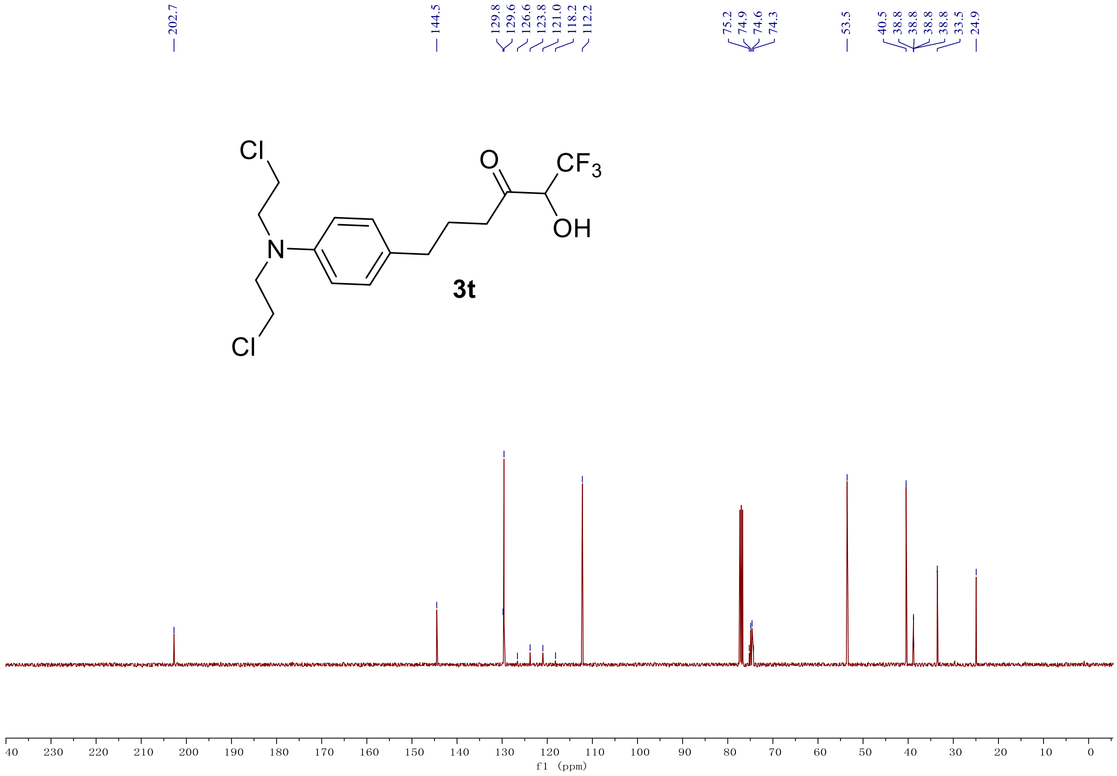

^{1}H NMR (400 MHz, $CDCl_3$)

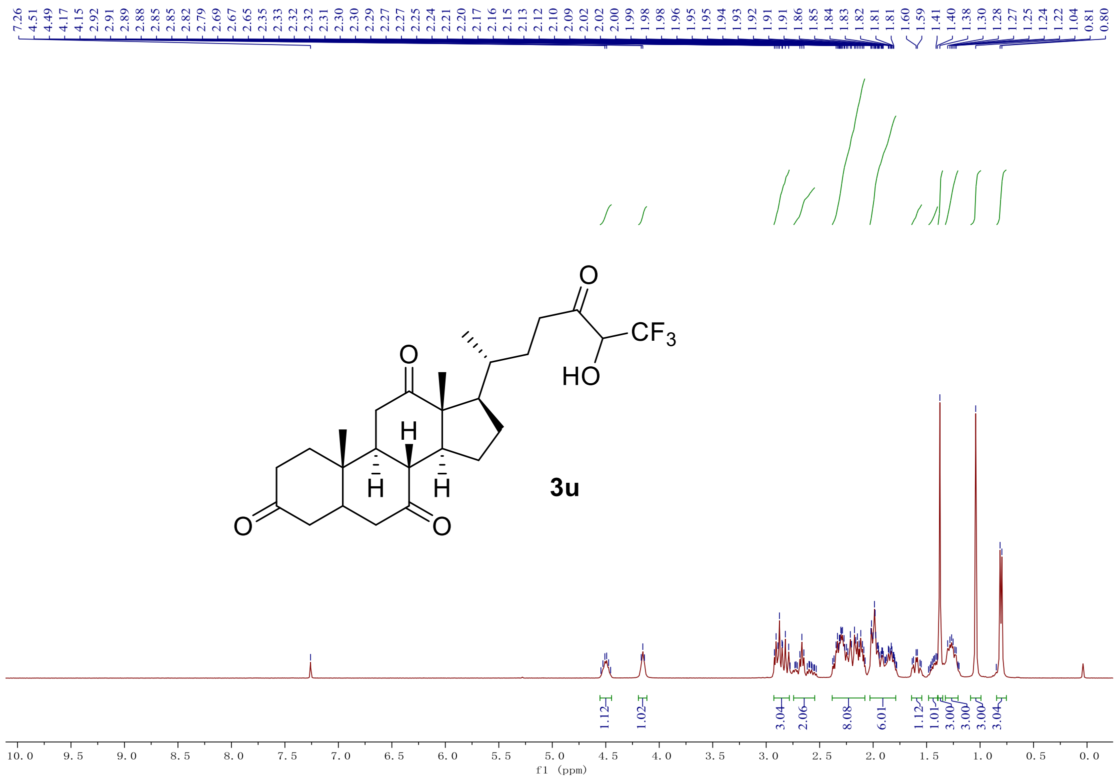

^{19}F NMR (376 MHz, $CDCl_3$)

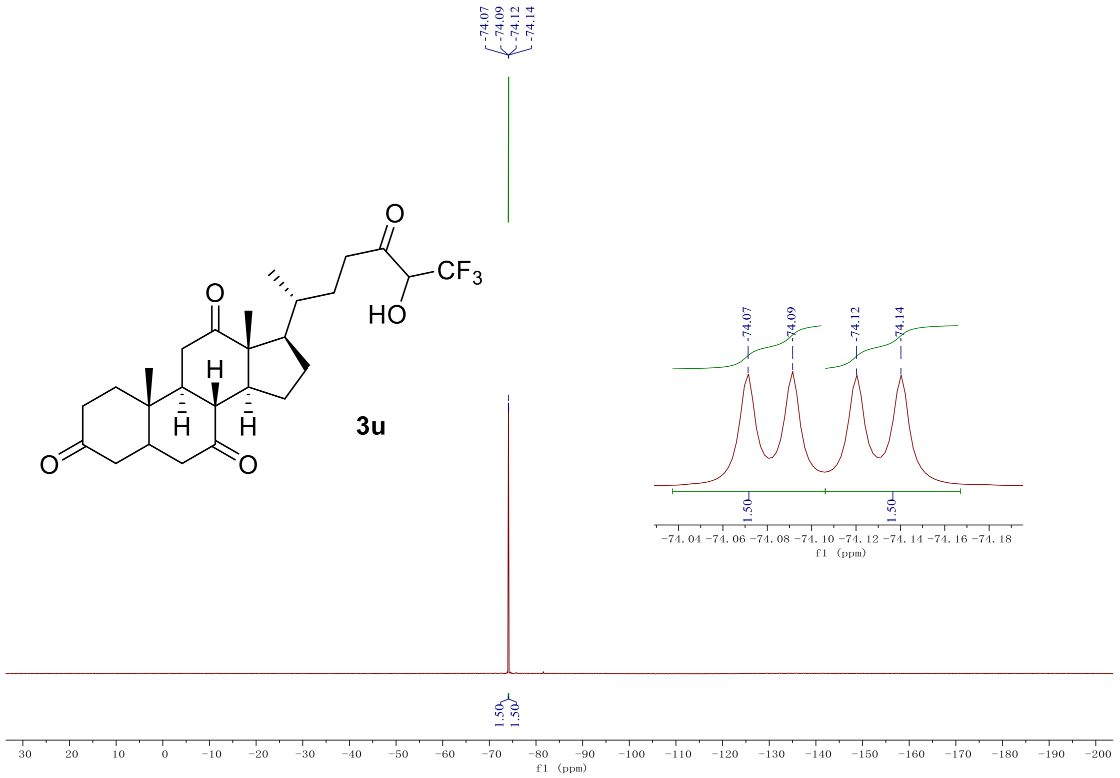

^{13}C NMR (101 MHz, $CDCl_3$)

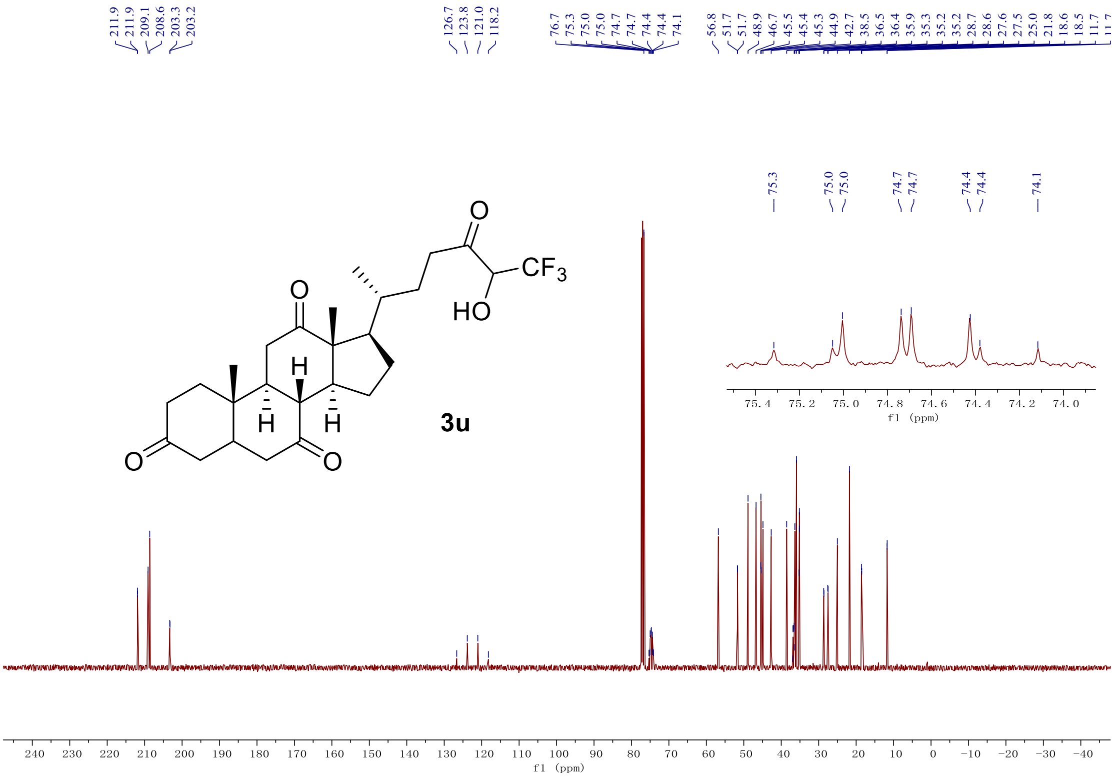

Supplement: File 1 — Experimental procedures, product characterization, and copies of NMR spectra. [file Beilstein_J_Org_Chem-19-1372-s001.pdf]
